# NNVAWI Abstracts

**DOI:** 10.1177/2333393620932494

**Published:** 2020-07-01

## The Enhanced Maternal and Child Health Nurse Home Visiting Program in Victoria—Nurses Working With Women and Children Experiencing Family Violence

### Catina Adams^1^, Angela Taft^1^, and Leesa Hooker^1^

^1^La Trobe University, Melbourne, Victoria, Australia

**Research Question:** How do Enhanced Maternal and Child Health (EMCH) nurses and their supervisors work with women and children experiencing family violence?

**Background:** The Victorian Royal Commission Into Family Violence has generated increased focus on the screening, identifying, and support of women and children experiencing family violence. A state-wide online survey of the EMCH nurse home visiting program in 2017 confirmed that 20% of EMCH clients are experiencing family violence. EMCH nurses have a key role in identifying and supporting families, but the nature of this role has not been comprehensively described.

**Study Design** (including sample and data collection approach, and analysis): Forty EMCH nurses and their supervisors have been consulted using semi-structured interviews. Preliminary thematic analysis of the transcribed interviews has identified barriers and enablers for nurses working with families experiencing family violence. A subset of analysis relates to the experience of EMCH nurses working in rural and remote areas.

**Results:** The nurse and nurse supervisor interviews have confirmed significant variation in practice when nurses are supporting women and children experiencing family violence. The roll-out of a new EMCH program in 2019 has highlighted these differences, as EMCH nurses attempt to modify their practice to the new model. Nurses spoke about feelings of increasing responsibility in family violence work, and a lack of role definition and boundaries between nurses and specialist services.

**Implications:** With a clearer insight into the work of EMCH nurses and their supervisors, we aim to identify family violence practices, professional boundaries, and supports, to ensure that the work of the EMCH program contributes effectively to the support of women and children experiencing family violence.

## Cultural Considerations and Recommendations for HIV Testing Services Among Hispanic or Latin American Victims of Intimate Partner Violence

### Oluwamuyiwa Winifred Adebayo^1^ and Jessica Williams^2^

^1^The Pennsylvania State University, State College, Pennsylvania, USA

^2^University of North Carolina at Chapel Hill, Chapel Hill, North Carolina, USA

**Problem Statement:** HIV continues to be a health burden to the Hispanic or Latin American communities in the United States. Victims of intimate partner violence (IPV) are at a heightened risk for HIV infection and experience worsened health outcomes. A crucial challenge to prevention is the low rates of HIV testing and diagnoses. Recent studies point to the important role of culture in seeking or obtaining an HIV test; however, there is still a dearth of knowledge on how to promote and offer culturally tailored HIV testing service to Hispanic or Latin American victims of IPV. The purpose of this study was to explore the cultural influences and considerations for HIV testing among Hispanic victims of IPV.

**Method:** A qualitative descriptive study was conducted with 17 key informants that included women with a history of IPV, HIV service providers, and IPV service providers. Study participants were recruited from IPV service agencies and HIV testing sites in South Florida. Data were collected using a demographic questionnaire and audio-recorded individual in-depth interviews. Data were analyzed using conventional content analysis.

**Results:** The findings from this study describe the influence of culture in the HIV testing decisions of victims of IPV. In addition, findings from this study elicited recommendations on how to provide culturally tailored HIV testing services. The supporting themes from this study include Ambivalence, Silence, Powerlessness, Diversity, Competence, and Familiarity.

**Implications:** To effectively address the burden of HIV infection among Hispanic or Latin American victims of IPV, it is vital to understand the cultural considerations that influence HIV testing decisions. The findings from this study can be used to provide culturally tailored and trauma-informed HIV testing services to Hispanic or Latin American people in the United States.

## Meaning, Context, and Indigenous Structures for the Management of Family Violence in a Yoruba Subethnic Community: A Qualitative Pilot Study

### Adekemi E. Olowokere^1^, Oluwasayo B. Ogunlade^1^, Ojo Agunbiade^1^, Aanuoluwapo O. Olajubu^1^, and Oyeyemi O. Oyelade^1^

^1^Obafemi Awolowo University, Ile-Ife, Nigeria

Documented interventions for the management of family violence are based majorly on law enforcement and the judicial referral system in developed countries. This is contrary to the societal belief that happenings within the family cycle should be kept secret in the Nigerian setting. This makes the victim of family violence to suffer in silence and has often resulted in a lot of psychological disturbance and its attendant’s problem among affected couples. It will be of utmost importance as a preliminary study to a larger study aiming at developing an indigenous intervention for management of family violence to understand family violence as perceived by this population. This study explored the meaning and context of family violence and the indigenous structure use for its management among a Yoruba subethnic group. This was an exploratory cross-sectional study in which 20 community stakeholders were recruited through purposive sampling. The data were collected via in-depth interview (IDI) and focus group discussion (FGD). Data analysis was done using Atlas.ti qualitative software. The result shows that family violence was seen as being synonymous to all forms of physical violence that occurs within a family relationship. There was no single word in the subgroup that could be used to represent family violence. While other forms of family violence (sexual, psychological, and economical) were seen as causes of Family violence. Religious leaders were identified as key indigenous structure for effective management of family violence among other indigenous structures which include extended family mediation and community/royal father interventions. The study showed that these groups of people are also affected by the male dominance belief in the society which has contributed to men perpetration of violence in the family. The study concluded that an indigenous intervention that will help control family violence must focus on strengthening community structures, most importantly, the religious institutions on how to instill mutual respect among couples and train them on conflict resolution skills as family violence in the group is seen as issues that should not be taken out of community structure.

## Hair Cortisol as a Biomarker of Chronic Stress Among Saudi Women Who Are Survivors of Abuse

### Eman Alhalal^1^ and Rawaih Falatah^1^

^1^King Saud University, Riyadh, Saudi Arabia

**Problem and Purpose:** The current evidence is still inconsistency regarding the negative consequences of intimate partner violence (IPV) on HPA (hypothalamic, pituitary, adrenal) axis activation. The previous studies have mainly relayed on salivary and blood sampling, which both reflect acute stress instead of chronic stress. A novel approach is recently emerged to retrospectively look to cortisol concentration as a biomarker of HPA axis activity, yet this approach has not been used with Saudi women. Examining the biological outcomes in the context of IPV is significant in understanding the HPA axis activity of women as a result of experiencing IPV. This understanding could help in increasing early recognition of abused women at risk of chronic illness and tailoring the required intervention programs for these women. Moreover, this study could substantiate the effects of IPV on Saudi women’s health and disease, which has received little recognition within the medical community in Saudi Arabia. The aims of this study were to (a) examine whether there is any distinction in cortisol levels between women exposed to IPV and women without abuse experiences, and (b) estimate whether cortisol secretion is associated with severity of IPV, mental health conditions (post-traumatic stress disorder [PTSD] and depression), and women resilience.

**Method:** A cross-sectional design was used. A convenience sample of 162 women was recruited with having two groups depending on exposure to IPV (IPV group and control group). The recruitment of women took place in primary health care units and outpatient clinics in Saudi Arabia. Hair cortisol concentration was measured in 6-cm-long hair strands. During the structured interview, women’s body mass index (BMI), IPV severity, PTSD and depressive symptoms as well as women’s resilience were also assessed.

**Results:** The findings will be available before June 2020.

## The Effects of an Intimate Partner Violence Educational Intervention on Nurses: A Quasi-Experimental Study

### Eman Alhalal^1^

^1^King Saud University, Riyadh, Saudi Arabia

**Problem and Purpose:** Intimate partner violence (IPV) is a health issue that affects women globally. Nurses have a unique role in responding to the needs of IPV survivors. Yet, nurses are not adequately prepared to take on their roles in dealing with IPV. Developing IPV educational interventions for nurses is a crucial step to equip them with the necessary knowledge and improve their attitudes and clinical practices. The aim is to assess the impacts of IPV educational intervention on nurses’ knowledge, attitudes, and behaviors.

**Method:** A quasi-experimental design was used. A convenience sample of nurses was recruited from two hospitals in Saudi Arabia. Nurses (*n* = 114) in intervention and control groups completed both the pre- and post-intervention surveys, namely, the Physician Readiness to Manage Intimate partner violence Survey (PREMIS). The intervention involved a 3-hour IPV educational program.

**Results:** There were no statistically significant differences between the intervention and control groups in terms of demographic characteristics and the baseline variables that were measured. An analysis of covariance showed a significant effect of the intervention on perceived IPV knowledge, perceived IPV preparation, actual knowledge, and IPV attitudes after controlling for pre-test scores. However, the intervention did not significantly affect nurses’ practices.

**Conclusion:** The IPV educational program was effective in improving nurses’ perceived preparedness, knowledge, and attitudes. An improvement in nurses’ practices was not achieved, and this might have been affected by the organizational system. Further research is needed to assess the long-term improvement in practice. The health care system should deal with IPV as a health issue. The World Health Organization has underscored the importance of training health care professionals to ensure they are able to assess IPV survivors and provide appropriate services to them. IPV educational programs need to be integrated into nursing curricula and in-service training.

## Sexual Violence on College Campuses: Opportunities for Improvement in Research and Practice

### Jocelyn C. Anderson^1^, RaeAnn E. Anderson^2^, and Candace Burton^3^

^1^The Pennsylvania State University, State College, Pennsylvania, USA

^2^University of North Dakota, Grand Forks, North Dakota, USA

^3^University of California–Irvine, Irvine, California, USA

Sexual violence (SV) continues to be prevalent on college campuses. Despite high rates of SV, most students do not seek formal services to address these experiences, leaving them open to a myriad of negative consequences. This symposia will present findings from three unique studies to highlight opportunities for improving our understanding of and response to SV on campuses. First, we examine how measurement affects our understanding of SV among those reporting partners as perpetrators of violence. Results from a mixed-methods study examining how women of color experience reporting on campus and a randomized controlled trial of a health center–based prevention and response intervention will provide insights into improving services for students.

Modifying the Sexual Experiences Survey to Assess Intimate Partner Sexual Violence Among College Women

RaeAnn Anderson, Samantha Holmes, Nicole Johnson, and Dawn Johnson

Research highlights the existing lack of precision in assessing experiences of SV. The assessment of intimate partner sexual violence (IPSV) typifies the challenges of measurement in two areas of violence research—intimate partner violence (IPV) and SV. The goal of this study was to evaluate strategies for assessing IPSV and how a modified version of the Sexual Experiences Survey–Short Form Victimization (SES-SFV) may improve measurement of IPSV. Two samples of college women were recruited. In Sample 1 (*N* = 236), we compared the number of IPSV cases identified by the Severity of Violence Against Women Scales (SVAWS) and a modified version of the SES-SFV. In Sample 2 (*N* = 207), participants completed the SVAWS and were randomly assigned to either a traditional SES-SFV or the modified SES-SFV. The rates of IPSV were nearly double when participants received the modified SES-SFV compared with the SVAWS. Results suggest that optimal measurement of IPSV needs to combine strategies currently used in the fields of IPV and SV.

Reporting Intimate Partner Violence and Sexual Violence: A Mixed-Methods Study of Concerns and Considerations Among College Women of Color

Candace Burton, Jeanine Guidry, and Jessica Cabrera

Little is known about how women of color evaluate the benefits of reporting IPV/SV to authorities but evidence suggests that they often choose not to report. The purpose of this mixed-methods study was to explore how university-affiliated women of color experienced structural stress in their daily lives and whether or not that stress influenced their thinking about the possibility of reporting IPV/SV to authorities. Participants identifying as Latinx/Hispanic or Black/African American reported the highest such stress, and felt that there was not always a potential gain in safety with reporting IPV/SV.

Giving Information for Trauma Support and Safety: A Campus Health Center Intervention to Address Sexual Violence

Elizabeth Miller, Kelley Jones, Heather McCauley, Jocelyn Anderson, Carla Chugani, Robert Coulter, Janine Talis, Dana Rofey, Duncan Clark, and Kalaeb Abebe

This cluster randomized controlled trial (RCT) collected data from 2,291 students on 28 college campuses. Intervention site providers received training and support in implementing the Giving Information for Trauma Support and Safety (GIFTSS) intervention; control site providers implemented a brief alcohol counseling intervention. Generalized linear mixed models accounting for within-college clustering were used to test for differences in change from baseline to follow-up by treatment arm. Students reported high rates of past 30-day alcohol use (74%), binge drinking (50%), and lifetime SV (55%). Intervention implementation varied widely by site (17%–91%). Overall, clinic staff found the intervention useful and agreed it could be implemented at the provider level. Primary implementation barriers were time and competing patient priorities. Providers noted variation in implementation based on patient and visit characteristics (e.g., patient gender). Clinic support—particularly in adopting strategies for universal dissemination of the GIFTSS card and prompts within the medical record—was seen as helpful.

## Strengthening Health Systems’ Response to Violence Against Women: Implementation Research Among Health Care Providers in Maharashtra, India

### Sanjida Arora^1^, Prachi Avalaskar^1^, Sangeeta Rege^1^, Padma Deosthali^1^, Sarah Meyer^2^, and Avni Amin^2^

^1^Centre for Enquiry Into Health and Allied Themes (CEHAT), Mumbai, India

^2^World Health Organization, Geneva, Switzerland

**Problem Statement:** In recognition of the unique role of health care providers in responding to violence against women, in 2013, the World Health Organization (WHO) published clinical and policy guidelines, “Responding to Intimate Partner Violence and Sexual Violence Against Women.” Little is known about adaptation of the Guidelines to specific contexts in low- and middle-income contexts.

**Research Objectives:** We conducted a mixed-methods implementation research study, including assessing needs and priorities of nurses in responding to violence against women; and adapting, implementing the training, and assessing improvements in nurses’ knowledge, attitudes, and practices (KAP). This paper focuses on nurses’ involvement and experiences in the study.

**Study Design:** To inform adaptation and implementation of training activities, we held a 2-day stakeholders meeting, including nurses from three tertiary care hospital in Miraj, Sanja, and Aurangabad, Maharashtra. A total of 105 nurses responded to a self-administered KAP survey immediately prior and after, and 6 months following training on responding to violence against women in the health system. Quantitative analysis, conducted in SPSS, included comparison of levels of KAP prior to, immediately after, and 6 months after training, and generalizing estimate equation (GEE) models to account for correlations between baseline and follow-up KAP levels.

**Results:** In the stakeholder meeting, nurses described barriers to responding to violence against women in health facilities, including lack of time, lack of adequate personnel, fear of backlash from family members, and lack of support from system. Descriptions of patient flow and roles and responsibilities of various cadres informed adaptation of the training. Results from the KAP survey of nurses indicated that knowledge regarding, attitudes toward, and clinical practices focused on response to violence against women improved.

**Implications:** Our implementation research study provides insights into an effective methodology for adapting, implementing, and evaluating a systems approach to improving health care response to violence against women. Moreover, it highlights the role of training of nurses to improve health care response to violence against women in a low-resource setting, as well as providing evidence as to replicable processes for implementing the WHO Guidelines in context-specific and sensitive ways.

## Structural Factors Influencing Experiences of Intimate Partner Violence for Indigenous Women in the United States

### Joyell Arscott^1^, Brittany Jock^1^, Meredith Bagwell-Gray^2^, Emily Loerzel^3^, Richelle Bolyard^1^, Teresa Brockie^1^, Victoria O’Keefe^4^, Catherine McKinley^5^, Jacquelyn Campbell^1^, and Gail Dana-Sacco^1^

^1^Johns Hopkins University School of Nursing, Baltimore, Maryland, USA

^2^The University of Kansas School of Social Welfare, Lawrence, Kansas, USA

^3^University of Washington, Seattle, Washington, USA

^4^Johns Hopkins Bloomberg School of Public Health, Baltimore, Maryland, USA

^5^Tulane University School of Social Work, New Orleans, Louisiana, USA

**Problem Statement:** Indigenous women in the United States experience higher rates of intimate partner violence (IPV), intimate partner homicide, and sexual assault, compared with women of other races and ethnicities. Previous research on IPV has not focused on the impact of structural racism and discrimination on Indigenous women in this context.

**Purpose:** Explore the influence of family, legal, and economic systems that affect the IPV experiences of Indigenous women.

**Study Design:** This was Phase 1 (qualitative) of a sequential mixed-methods design to develop and evaluate a culturally specific version of myPlan intervention for Indigenous abused women.

**Sample:** Participants were purposefully sampled using referrals from partner organizations serving Indigenous women in the Southeastern, Southwestern, and Northeastern regions of the United States from July 2016 to June 2017.

**Data Collection:** In-depth qualitative interviews with Indigenous women with IPV experience (*n* = 42) and in-depth interviews and focus groups with physical and mental health, and social service providers with experience serving Indigenous IPV survivors (*n* = 41).

**Analysis:** A Grounded Theory–informed methodology was used to identify emergent themes related to Indigenous women’s risk and protective factors relevant to IPV. The three prominent focused codes chosen for secondary analysis were the roles of family and community networks, legal systems, and economics.

**Results:** Participants described how both survivor and abuser’s family networks had a significant impact on Indigenous women’s ability to get support. Participants identified persistent individual and intergenerational challenges related to lack of access to the protective features of legal and public service systems due to jurisdictional complexities that interact with politics, privilege, geographic isolation, and lack of resources. Participants discussed how poor access to reliable transportation (public and private) had a direct effect on Indigenous families’ ability to find stable employment, safe housing, and financial security, creating multiple stressors, described as increasing risk of IPV for Indigenous women.

**Implications:** Understanding how social and structural factors increase Indigenous women’s vulnerability to IPV inform nursing’s approach to policy, advocacy, and translational research for IPV. Nursing research utilizing multilevel interventions also needs to be developed and evaluated to address IPV among Indigenous women.

## Methodological Innovation to Explore Obstetric Violence in Brazil by a Multiregional Critical Ethnography

### Margareth S. Zanchetta^1^, Sepali Guruge^1^, Oona St. Amant^1^, Ingryd C. Ventura Felipe^1^, Dakota Carrie^1^, Dorin d’Souza^1^, Vanessa Fofie^1^, Hilary Hwu^1^, Milena Oliva^1^, Hannah Stahl^1^, John Tadeo^1^, Walterlânia Silva Santos^2^, Hannah Argumedo-Stenner^1^, Francesca Aviv^1^, Henry Parada^1^, Karline Wilson-Mitchell^1^, Delmar Teixeira Gomes^3^, Zuleyce Lessa^3^, Jordan Tustin^1^, Erica Dumont^4^, Kleyde Ventura^4^, Waglânia Freitas^5^, and Ana Luiza de Oliveira Carvalho^6^

^1^Ryerson University, Toronto, Ontario, Canada

^2^University of Brasília, Brasília, Brazil

^3^Federal University of Juiz de Fora, Juiz de Fora, Brazil

^4^Federal University of Minas Gerais, Belo Horizonte, Brazil

^5^Federal University of Paraíba, João Pessoa, Brazil

^6^Federal University of Rio de Janeiro, Rio de Janeiro, Brazil

**Problem Statement:** Obstetric violence (OV) represents disrespect, abuse, and neglect by health care professionals (HCPs) during labor and birth. To address OV, Brazil has implemented the Humanization of Labour and Birth Policy (HLBP). The perspectives and opinions of Brazilian families and HCPs regarding OV are unknown and there remains a dearth of data and tools to explore OV and its effects on women in Brazil. Researchers from one Canadian university and eight Brazilian universities collaborated for a critical ethnography to design and pilot data collection tools designed to manage methodological and cultural nuances related to OV in Brazilian context.

**Purpose:** This paper reports on the design and implementation of online and paper-based structured questionnaires, semi-structured interview, and focus group guides to document/measure OV. Tools also document OV awareness among community and professional stakeholders with the intention of facilitating dialogue or recommendations for improved implementation of the HLBP.

**Design:** Using the conceptual perspectives and indicators of OV, Canadian nursing and social work undergraduate students conducted literature reviews, identified key themes, and proposed preliminary drafts of questions. Canadian faculty consulted with the students to confirm and validate interpretations, and refinement of proposed interview questions. Brazilian co-investigators, obstetric nurses, further reviewed and refined the tools, and ensured proper translation to Portuguese.

**Results:** Preliminary findings were from first 963 participant interviews (of 4,000 planned). We will describe the iterative process by which students and supervisors were challenged to paraphrase the questions into region-specific language and navigate semantic meanings. Respondents demonstrated better understanding of structured questions.

**Implications:** Research tools are grounded in local cultures of acknowledgment of OV and provoked discourse with all stakeholders. Findings will inform tools’ validation, translation to policy makers, educators, community members, and frontline workers in Brazil and beyond.

## Strengthening the Health System Response to Domestic Violence in Occupied Palestinian Territory

### Loraine J. Bacchus^1^, Abdulsalam Alkaiyat^2^, Amira Shaheen^2^, Ahmed Alkhayyat^2^, Hiba Od^2^, Rana Halaseh^2^, Gene Feder^3^, and Manuela Colombini^1^

^1^London School of Hygiene & Tropical Medicine, London, United Kingdom

^2^An-Najah National University, Nablus, Palestine

^3^University of Bristol, Bristol, United Kingdom

**Problem Statement:** There is a need to understand health system readiness and structural context when integrating and scaling up promising interventions to address violence against women.

**Method:** HERA (HEalthcare Responding to violence and Abuse) aimed to improve the readiness of primary health care providers and the health system to respond to women exposed to domestic violence in the West Bank, in occupied Palestinian Territory (oPT). HERA included two initial training sessions and reinforcement activities. A Clinic Case Manager was given additional training to be the first point of support for women and coordinate referrals to the external Ministry of Health–based Gender-Based Violence (GBV) Focal Point. Training was delivered by Juzoor, a local nongovernmental organization (NGO), together with a clinical co-trainer. Data collection included semi-structured interviews with women, trainers and health care providers, and key stakeholders; facility observations; and routine clinic data. Constructs from the implementation theory were used as sensitizing devices in the thematic analysis.

**Results:** Findings from the health system’s readiness assessment drew attention to specific preparedness deficiencies in the oPT health system and informed intervention adaptation and the evaluation. Evaluation findings reveal how the intervention interacted with political, sociocultural, and economic aspects of the context in oPT, creating unpredictability and uncertainty. Adaptive mechanisms used by providers and women to make the intervention workable included improvisation of practice and roles, controls over knowledge, and policing behaviors (of self and others). Referral of women to the external GBV Focal Points did not occur as anticipated and cases were maintained at clinic level. The Clinic Case Manager’s role evolved into one that involved alleviating women’s psychological distress.

**Implications:** Assessing health systems readiness assessment informed intervention adaptation and enhanced. The evaluation surfaced the different ways that participants negotiated the context to integrate the intervention components and make them workable (i.e., capabilities) and in turn, how this affected the capacity and potential of the system to accommodate implementation processes. More resources need to be allocated to the Clinic Case Manager role and management support to providers to alleviate fears about the threat of retaliation.

## What Now: Exploring The Emergency Health Care Response To Domestic Violence in Regional Hospitals

### Shannon Bakon^1^, Annabel Taylor^1^, Silke Meyer^2^, and Mark Scott^3^

^1^CQUniversity Brisbane, Brisbane, Queensland, Australia

^2^Monash University, Melbourne, Victoria, Australia

^3^Caboolture Hospital, Caboolture, Queensland, Australia

**Problem Statement:** Domestic violence is a form of gendered violence which has an unequal impact on the lives of women. This form of violence has become an international problem with high prevalence rates globally. Comprehensive services that prioritize the needs of victims and survivors of domestic violence should be provided by health care institutions. Frontline health care professionals such as doctors, nurses, and social workers within the emergency department are often the first point of contact for women experiencing domestic violence. Therefore, how these health care professionals respond to and provide care needs to be understood.

**Objective:** The purpose of this study was to explore emergency health care professional responses to patients experiencing domestic violence in regional Queensland hospitals within Australia.

**Study Design:** This qualitative study employed a Straussian grounded theory methodology.

**Sample:** The target participant population for this study were both male and female medical professionals, registered nurses, and social workers who were employed within the emergency department of two regional Queensland hospitals within Australia.

**Data Collection Approach:** The study collected data in two forms. First, demographic data were collected through anonymous paper-based surveys. Second, data on the care provided to patients experiencing domestic violence were collected through audio-recorded semi-structured interviews.

**Analysis:** The demographic data collected from the anonymous paper-based surveys were analyzed employing descriptive statistics. The audio-recorded semi-structured interviews was analyzed utilizing a Straussian Grounded Theory; a form of constant comparative analysis.

**Results:** This study highlights gaps in the emergency care provided to women experiencing domestic violence. It provides evidence and an overview of the care that is currently being provided. The experience of care provision and the different perspectives are drawn from the areas of medicine, nursing, and social work.

**Implications:** A designated domestic violence specialist role within the hospital setting and specifically within the emergency department may aid in increasing staff awareness and education. Saturation training of emergency health care professionals may also aid in reducing knowledge gaps and promoting the provision of patient-oriented health care. The development of a protocol to guide the provision of care may result in the standardization of care and increased referral to specialist support services.

## The Association Between Intimate Partner Violence and Functional Gastrointestinal Disorders and Symptoms Among Adult Women: A Systematic Review

### Ohud Banjar^1^, Marilyn Ford-Gilboe^1^, Deanna Befus^1^, and Bayan Alilyyani^1^

^1^Western University, London, Ontario, Canada

**Problem:** Functional gastrointestinal disorders (FGIDs) and symptoms have been identified as health consequences of intimate partner violence (IPV), with significant burdens for women. However, whether specific types of abuse (i.e., psychological, physical and sexual) affect the health of women living with FGIDs in different ways, and the mechanisms that explain these impacts on their health and quality of life are not well understood.

**Purpose:** This systematic review was conducted to (a) examine the association between types of IPV (i.e., physical, sexual, and psychological abuse) and risk of FGIDs and symptoms (such as chronic abdominal pain symptoms, irritable bowel syndrome (IBS), functional dyspepsia, frequent diarrhea, frequent constipation, and vomiting and nausea disorders) among adult women, (b) identify the mechanisms that mediate and/or moderate these health effects, and (c) assess the impact of FGIDs and symptoms on women’s quality of life.

**Method:** Using the PRISMA guideline, searches of selected electronic databases (PubMed, CINAHL, Cochrane Database of Systematic Reviews, ProQuest-Nursing & Allied Health, PsycINFO, Scopus, and Social Work Abstracts) were conducted for English language studies of adult women (18 years or older) who had experienced IPV and reported FGIDs and symptoms. Both quantitative descriptive (i.e., ecological, cross-sectional, cohort, and case-control studies) and qualitative studies were included, with no time frame for publication specified. Quality assessment of each included study was completed using published guidelines adapted from Hoya for quantitative studies and the Critical Skills Appraisal Program (CASP) tool for qualitative studies.

**Results:** In all, 1,444 unique records were initially identified. After Level 1 abstract screening by two reviewers, 1,393 records were excluded and 51 potentially relevant manuscripts remained. Level 2 full-text review yielded 15 included studies. Preliminary results suggest that there is an association between various types of IPV and FGIDs and symptoms and some factors, such as stress, appear to mediate and/or moderate this association. Results of this study may be useful in identifying the types of practice interventions that could mitigate the consequences of IPV on the health of women living with FGIDs. Final results will be available by January 2020.

## Considering Trauma- and Violence-Informed Care in the Canadian and Rwandan Contexts

### Helene Berman^1^, Nadine Wathen^1^, Vincent Sezibera^2^, Clementine Kanazayire^2^, and Aimee Utuza Josephine^1^

^1^University of Western Ontario, London, Ontario, Canada

^2^University of Rwanda, Kigali, Rwanda

**Overview of an Interdisciplinary Development Initiative:** Helene Berman

Globally it is estimated that one out of every three women continue to report some form of violence each year. The health effects are profound, effecting virtually every aspect of everyday life; yet, so often these effects are insidious, hidden from even the most astute health providers. In recent years, the notion of Trauma- and Violence-Informed Care (TVIC) has gained considerable traction, particularly within Canada. In contrast to the more commonly understood construct of Trauma-Informed Care, TVIC is broadly conceptualized to take into account multiple and intersecting forms and effects of structural, systemic, and interpersonal violence. The ultimate goal is the creation of safe environments and care interactions for clients and staff. In this Symposium, we describe an ongoing collaboration to address global health equity in general, and TVIC in particular, among colleagues in Canada and Rwanda.

**TVIC in the Canadian Context: The Gender, Trauma, & Violence Knowledge Incubator:** Nadine Wathen

Interest in TVIC in Canada continues to expand, even explode. Health and social service organizations from public health to policing and child welfare have sought training and resources to support trauma- and violence-informed policy and practice. In response, a group of community leaders, service providers, and researchers has come together as the Gender, Trauma, & Violence Knowledge Incubator (GTV Incubator). GTV Incubator members have delivered dozens of workshops, have developed capacity through student projects, and are designing online curriculum to spread the reach of these efforts. This presentation will provide an overview of the core principles of TVIC and highlight several research projects that are examining TVIC mobilization, uptake, and impact, including through health promotion to the public. We will also comment on our progress in developing appropriate metrics and methods for research and evaluation of TVIC initiatives.

**Why TVIC Makes Sense in the Rwanda Context:** Vincent Sezibera and Clementine Kanazayire

Violence against women, including physical, sexual, and emotional harm, is of great concern to humanity. In Rwanda, women’s lifetime experience of physical or sexual violence almost doubled from 34% in 2005 to 56% in 2010, placing this country among those with high prevalence domestic violence versus gender-based violence (DHS reports). Moreover, recent mental health reports in Rwanda have estimated that one third of the population presents with depression comorbid to the traumas related to post-traumatic stress disorder (PTSD) from the genocide. Both violence and the traumas from the genocide are assumed to be predictors of various mental and social difficulties, including drug and alcohol addictions, school drop-outs, poor academic and professional performance, and lowered life satisfaction. While TVIC is not a widely understood concept in Rwanda, we believe that it holds a great deal of relevance in the post-genocide context. We examine this idea and consider how TVIC can assist policy makers to elaborate strategies that mitigate adverse outcomes from violence and the genocide.

**The Potential Relevance of TVIC in Addressing Teen Pregnancy in Rwanda:** Aimee Utuza Josephine

More than 50% of the Rwandan population is less than 20 years old and the median age of the population is 22.7 years old. At the time of the 1994 Genocide against the Tutsi people of Rwanda, I was a young adolescent. Since that time, I have witnessed and experienced consequences of the genocide on the general population, and especially on women, children, and youth. The “Post” genocide reparation commitment from the government helped Rwanda to be recognized for its progress in achieving the Millennium Development Goals (MDGs), including the Promotion of Gender Equality and Empowerment of Women. However, unplanned pregnancies, particularly among adolescent girls, have remained a significant challenge. The negative physical and mental health sequelae are enormous, and it is clear that innovative solutions are needed. In this presentation, I consider the relevance of TVIC as a potential model to address the challenges of teen pregnancy in a manner that is holistic, empowering, and relevant.

## “ . . . But How Do I Ask That?” Adapting the Tag Team Simulation Methodology to Support Undergraduate Health Students’ Confidence, Understanding, and Skills When Working With Families Affected by Family Violence

### Laura Biggs^1^ and Stephen Guinea^1^

^1^Australian Catholic University, Fitzroy, Victoria, Australia

**Background:** In order for future health professionals to appropriately respond to domestic violence, there is a need for universities to provide effective, engaging and meaningful learning opportunities. Despite this, there are few examples available of domestic violence education for undergraduate or post-registration nurses and midwives. Most of the available education research relates to physicians, with less known regarding education for nurses and midwives working in hospital, clinic, and community settings. The need to provide meaningful learning opportunities is clear, with a large number of studies reporting that nurses and midwives feel unprepared to appropriately identify and respond to disclosures of family violence. In response to this, a team from Australian Catholic University has adapted the Tag Team Simulation (TTS) methodology to facilitate undergraduate health professional learning in relation to domestic violence. Informed by educational theory, TTS was originally designed to provide an engaging simulation activity which enables students to experience different approaches to a practice-based situation, and to witness the impact of each approach. Importantly, TTS has been adapted to help students develop the resilience and advanced communication skills required for complex and challenging situations. Evaluation of this methodology have shown high levels of participant satisfaction, and students report that they were able to learn how to respond to unexpected situations in a psychologically safe learning environment. The TTS methodology is easily adaptable to a range of professions and practice settings. Furthermore, it has been designed to provide large student numbers with an engaging and meaningful simulation experience, ensuring every student is an active participant in the simulation.

**Aims:** This symposium will be delivered as a hands-on workshop. The aims of the workshop are

- To introduce participants to the fundamental concepts and principles of TTS methodology;
- To provide attendees with an experience of the Tag Team methodology developed for preparing undergraduate students for working with families affected by gender-based violence;
- To discuss the techniques required for applying this TTS in participants’ own practice settings.

**Audience:** This workshop will be valuable for all attendees involved in health professional education and clinical facilitation.

## Adaptation of the Danger Assessment for Thai Women: A Delphi Study

### Tipparat Udmuangpia^1,2^, Supawadee Thaewpia^1^, Wacharee Amornrojawutthi^1^, Prapatsri Shawong^1^, Yaowaret Kamanat^3^, Pilin Nisatea^4^, and Tina Bloom^2^

^1^Boromarajonani College of Nursing, Khon Kaen, Thailand

^2^University of Missouri, Columbia, Missouri, USA

^3^Khon Kaen Hospital, Khon Kaen, Thailand

^4^Sakon Nakhon Hospital, Sakon Nakhon, Thailand

**Background:** The Danger Assessment (DA; www.dangerassessment.org) with 20 items is a tool to predict the risk of severe repeat or lethal violence, and help clinicians appropriately assist intimate partner violence (IPV) survivors in making risk-informed safety plans. However, the DA does not exist in Thailand. Therefore, the purpose of this study was to translate and construct the contents of the DA to be appropriate to Thai culture before rigorous determining of the predictive factors and psychometric properties in the next study.

**Method:** A three-round Delphi study was carried out by establishing a panel of Thai IPV experts. The DA was translated into Thai and back-translated into English by three experienced bilingual IPV experts. The experts were volunteers who work with Thai survivors for at least 2 years. After they agreed to participate in this study, the online statements of DA were sent to experts by private social media (Line app, email, and Facebook) in all rounds and 2 weeks were given for responding to each round. Responding of experts in each round was de-identified data in all rounds. Each question demonstrating ≥70% agreement on a 4-point Likert-type scale was determined to have reached consensus. Descriptive statistics were used. Qualitative feedback from experts in each round was also analyzed and discussed.

**Results:** A total of 27 experts responded and the majority of them were nurses and social workers with 10.9 years of average working experience with abused women. The average score of each item was between 3.29 and 3.49 in last round. There were eight items with nine subitems totally added. Examples of items that were added related to “addicted gambling, financial problem, cheating, and behaved sexually with children.”

**Conclusion:** This study will contribute to having the first-ever, validated Thai-language version of the tool for use in the Thai context, thereby contributing to better care and services for abusive population. Our next study will interview survivors about their experiences of abuse and ask them to review and provide feedback on the DA-Thailand; after that the DA-Thailand will be ready for a longitudinal study to validate its effectiveness in the Thai context.

## Screening for Intimate Partner Violence and Sexual Violence in a College Health Clinic

### Toni Boyajian^1^ and Claire Bode^1^

^1^University of Maryland–Baltimore, Baltimore, Maryland, USA

Intimate partner violence (IPV) and sexual violence (SV) are serious, preventable public health problems that affect millions of Americans.^1^ A national survey revealed a third of women and men experience IPV/SV in their lifetime.^2^ IPV/SV occurs across the lifespan and more often in certain life stages. More than half of individuals who have experienced violence report their first incident before 25 years old.^3^ A national college health survey revealed 9% of students experienced an abusive relationship. Nine percent report having been sexually touched without consent within the past 12 months.^4^ An opportunity exists for early detection and support services for IPV/SV within the young adult population and health clinic setting. Health care providers (HCPs) in college health clinics (CHCs) seldom screen for IPV/SV. Ninety percent of university students who had experienced IPV/SV reported not being asked at their most recent visit to their CHC.^5^ Only 15% of HCPs in CHCs reported screening for IPV/SV.^6^ At a mid-Atlantic public university, only screening for “forced or coerced sex” is routinely asked during specific visits. There is strong recommendation to screen universally for IPV/SV, particularly in CHCs.^7^ Routine and universal screening using the E-HITS screening tool^8^ will be implemented for 13 weeks in a CHC. The screening tool will be integrated into the patient preparation process and electronic health record. HCPs will be supported by trauma-informed training with a focus on IPV/SV and campus/community resources. Students will complete the screening in the exam room privately. A statement of confidentiality, limits of confidentiality, and reporting will be included and reviewed prior to completing the tool. The data collection will be the number of patients screened and the number of patients seen. In addition, data on the number of patients with positive screens who are referred for counseling and/or provided with resources will be collected as well. These data will be analyzed and findings will be discussed with the key stakeholders. Strategies for next steps will be discussed to allow the continuation of IPV/SV screening at this CHC.

## An Integrative Review of Community Nurse–Led Interventions to Identify and Respond to Domestic Abuse in the Postnatal Period

### Marie Boyle^1^

^1^Community Health Organisation, Limerick, Ireland

**Problem Statement:** Domestic abuse is a global issue and in Ireland, one in four women experience domestic abuse, resulting in detrimental long-lasting health and social impacts for both women and children. Professionals such as Public Health Nurses (PHNs), who have a mandate to see all postnatal mothers, are challenged to respond appropriately and compassionately, while ensuring the safety of women and children is central to ongoing service development.

**Research Purpose:** This integrative review, undertaken to support the development and implementation of a PHN-led domestic abuse initiative, examines community nurse–led domestic abuse interventions, to identify and respond to domestic abuse in the postnatal period.

**Method:** An integrative review, where six electronic databases and peer-reviewed journals were searched for research papers published between January 1, 2005, and March 1, 2019.

**Results:** Fifteen papers met the inclusion criteria. Outcomes of the short-term interventions included an increase in routine enquiry, documentation of alone status, and safety planning; however, referrals remained low. There was a reduction in victimization seen in intensive home visiting interventions, where there were multiple points of contact over a 2-year period. One study did report potential harm to mothers already experiencing domestic abuse prior to the intervention. Thematic analysis generated four themes: (a) benefits to women and nurses, (b) approaches to domestic abuse identification and response interventions, (c) implementation of community nurse–led domestic abuse interventions, and (d) the barriers and facilitators to implementation and sustainability.

**Implications:** The safety implications when dealing with domestic abuse are especially challenging in the community setting, with lone working and remote access to specialist services. However, PHNs are well placed to provide a proactive response, including safety planning, to postnatal women experiencing domestic abuse, provided the appropriate supports are in place such as

- Interagency training, including refresher updating, supervision, and mentorship;
- Clear guidelines, referral pathways, safety protocols, and safety planning guidance;
- Collaborative working, with the development of stronger links with domestic abuse services;
- Organizational support with enhanced resources; and
- Commitment at government level to the provision of domestic abuse services.

## The Experience of Trauma and Post-Traumatic Growth

### Hulda Saedis Bryngeirsdottir^1^

^1^University of Akureyri, Akureyri, Iceland

Research on the effects of trauma have largely focused on their negative consequences. The purpose of this study was to increase knowledge and deepen understanding of psychological trauma and how people achieve post-traumatic growth. Phenomenology was chosen as a research approach. Data were collected in 14 interviews with 12 individuals who had experienced psychological trauma and post-traumatic growth. Participants were aged 34 to 52 years, seven women and five men. The title of the study, “Like Going Down a Black Slope and Then Coming Up the Green Hillside,” metaphorically describes the participants’ experience of trauma and post-traumatic growth. It describes the difficult journey which started with the trauma. The participants felt powerless after the trauma but considered internal factors, such as perseverance, hardiness, and the courage to face their situation the most important in their processing of the trauma. They all experienced more traumas on the journey and expressed the need for support and caring in their situation. They also noted the positive impact of dealing with new projects. All participants felt that the onset of post-traumatic growth was due to an internal need for change. They experienced that their post-traumatic growth was characterized by improved and deeper relationships with others, increased personal development, positive living, increased self-knowledge and improved self-esteem. Participants described “heavy days” despite the post-traumatic growth, but they still felt like winners. Research findings indicate that the lived experience of trauma is a challenging life experience, but certain internal factors are prerequisites for post-traumatic growth. It is important that nurses and other professionals respond to their clients’ traumas through early detection and intervention, along with support, caring, and follow-up.

## Community Advisories: Research Partners or Research Facilitators in Research With Women Experiencing Violence?

### Vicky Bungay^1^, Linda Dewar^2^, and the STRENGTH Project CAC^2^

^1^University of British Columbia, Vancouver, British Columbia, Canada

^2^Inner City Women’s Initiatives, Vancouver, British Columbia, Canada

**Problem:** Community advisory committees (CACs) with women experiencing violence are a frequent aspect of participatory research design and implementation. CAC engagement produces results highly relevant to community with added benefits of fostering research capacity and shifting academic–community power dynamics. Despite the lauded benefits, there are numerous critiques of research practices; particularly ethical concerns of tokenism, exclusion, unrealistic demands, and in some instances re-traumatization and harm.

**Purpose:** In this session, we illustrate how an innovative approach to the development and implementation of a women’s CAC grew into an effective community–academic research partnership and reshaped research practices in strategic and important ways.

**Approach:** We specifically draw on our experiences from a 3-year, pilot study examining the implementation of an innovative model of outreach to foster engagement of highly marginalized women with essential life sustaining services. We detail how integrating trauma- and violence-informed (TVI) approaches that simultaneously seek to foster participation and decision-making authority of the CAC positively affected the quality of the intervention and the health and well-being of the women participating in this group (e.g., building employable skills, reducing emergency room visits). Drawing upon field notes and interviews with CAC and research team members, we describe the role of community leadership, the timing and sequencing of activities necessary for effective research relationships, and the support infrastructure needed for CAC members who continue to experience complex health and social concerns daily.

**Lessons:** Building effective research partnerships with CACs is challenging, time-consuming, and rewarding. It is essential that the process be inclusionary and therefore engages women regularly excluded from such roles particularly those with mental health concerns, problematic substance use, and engaged in illegal activity. It requires partnership with services trusted by women to help facilitate attendance and foster capacity building to undertake these roles. It also requires researchers be willing to reshape traditional power dynamics and be open to reconsidering study design and implementation.

**Implications:** The exclusion of women experiencing violence from decision-making processes concerning programs and practices that directly affect their lives has contributed to harmful research practices and interventions with limited effectiveness and/or sustainability. TVI approaches combined with principles of full, participatory inclusion offer promising results for research quality and women’s everyday lives.

## Changing Campus Climate: Engaging Students in Reducing Tolerance for Sexual Assault and Intimate Partner Violence

### Candace Burton^1^

^1^University of California–Irvine, Irvine, California, USA

**Problem Statement:** Up to 36% of U.S. college and university students state they experienced unwanted sexual contact or intimate partner abuse during their higher education. Many do not identify the experience as assault, nor do they report to authorities.

**Purpose and/or Questions:** The purpose of this study was to engage students in exploring the campus climate and creating a culture of safety with regard to sexual assault (SA) and intimate partner violence (IPV). The specific aims were to (a) assess attitudes toward SA and IPV, beliefs about gender roles, and perceptions of campus climate with regard to SA and/or IPV, and (b) pilot test a novel educational intervention targeting understanding of the dynamics of SA and IPV, consent, boundary-setting, and the role of gender therein.

**Study Design:** The intervention consists of weekly seminar discussions, self-reflective writing, martial arts-based mindfulness, and physical space negotiation, as well as collective examination of environmental factors influencing campus climate with regard to SA and IPV.

**Sample:** We recruited a convenience sample of 54 college-attending individuals, ages 18 to 35.

**Data Collection:** Participants completed a series of questionnaires on demographics, attitudes toward SA and IPV, social supports, and beliefs about campus climate regarding SA and IPV. Surveys were completed at the beginning and the end of participation. Participants completed self-reflective writing via an anonymous online journal.

**Analysis:** Descriptive and inferential statistics were computed for each measure for the entire sample and for relevant subsamples. Reflective journal text was downloaded from the secure online entry and uploaded verbatim into ATLAS.ti software for analysis using a naturalistic inquiry framework.

**Results:** We found changes in participant scores for acceptability of controlling and abusive behaviors, attitudes toward SA, and beliefs about gender stringency following the intervention. Insights into IPV and SA occurring on campus included awareness of how gender norms influence behaviors.

**Implications:** Elucidating how students understand SA and IPV in the campus community and consider their safety and that of others, and exploring what factors drive them toward or away from reporting can support a campus climate less tolerant of violence and more supportive of survivors.

## Social Media in Research and Education: Yes, You Need It!

### Candace W. Burton^1^, Jeanine D. Guidry^2^, and RaeAnn E. Anderson^3^

^1^University of California–Irvine, Irvine, California, USA

^2^Virginia Commonwealth University, Richmond, Virginia, USA

^3^University of North Dakota, Grand Forks, North Dakota, USA

**Overview:** Social media is not going away! It represents both a powerful scientific tool and a potential point of crisis for many in the health care professions. Social media platforms offer opportunities to discover a variety of perspectives, information, and influences on issues of interest—but can also create vulnerabilities that are important to consider. This symposium will explore potentials within social media, its uses in research and education, and some ways nurses can best make use thereof. The aims of the symposium are to

- Review the current state of the science on social media and health
- Explore options for conducting research on and with social media
- Explore strategies for using social media in nursing education
- Demonstrate some methodologies for analyzing topics of interest via social media and for utilizing social media as an educational tool.

**Format:** This will be an interactive session with opportunities for attendees to strategize about ways to work with social media in their own areas of expertise. The presenters will share specific examples from their own work, with detail about applicable analytic methods and educational scenarios. Special attention will be paid to the use of social media to discover the perceptions and knowledge of specific issues among users, support the development of a culturally competent nursing workforce, and improve patient and provider education at all levels. Other relevant topics may be discussed at the discretion of the group.

Part I: State of the science on social media and health (15 minutes)

This section will provide an overview of the current state of social media, including platforms, uses, and controversies. Included in this overview will be some of the advantages and risks associated with social media in the area of health, some of the considerations for nurses on social media, and some basic suggestions for using and interacting with social media.

Part II: Using social media in research and education (45 minutes)

This section will provide some background on doing research with and using social media to support learning among nurses and nursing students. For research purposes, this can include exploring public opinions or perspectives on topics including intimate partner violence/sexual assault, other types of violence against women, and mental health–related topics. In education, there are tremendous opportunities to explore social media to develop patient education as well as to support student learning about diverse and vulnerable groups. Examples from panelists’ own work will be reviewed.

Studies to be discussed include

- Intimate partner violence on Instagram and Pinterest
- Hashtag-focused studies including #WhyIdidntreport and #NotOkay
- Exploration of young adults’ exposure to rape myths online

Strategies for educational use include

- Using social media for cultural competence
- Expert engagement
- Applying social media to student activities

Part III: Discussion of methodologies for working with social media (30 minutes)

This section will take a deeper dive into methods for conducting research using social media, and for using social media in nursing education and training. Social media interactions can be powerful as well as risky. Many topics can create vulnerability for both post authors and for researchers and educators working with social media. Care must be taken to avoid creating any ethically problematic situations as well as to adequately assess the content of the platform in question. Professional policy and regulatory considerations when working with social media will also be discussed.

## Violence and Migrant Women—Challenges and Opportunities in the Care Encounter

### Ulrika Byrskog^1^, Pia Olsson^2^, Birgitta Essén^2^, and Marie Klingberg Allvin^1^

^1^Dalarna University, Falun, Sweden

^2^Uppsala University, Uppsala, Sweden

**Problem Statement:** During the latest decades, an increasing number of Somali-born women with experiences from long-lasting war followed by migration have encountered Swedish maternity care, where antenatal care midwives are assigned to ask questions about exposure to violence.

**Purpose:** To gain deeper understanding of Somali-born women’s and Swedish midwives’ perspectives related to violence and violence inquiry during the multiple transitions of migration and motherhood, with the antenatal care encounter in focus.

**Study Design:** Qualitative individual interviews were conducted with Somali-born women and Swedish antenatal care midwives, analyzed by thematic analysis.

**Results:** Political violence with broken societal structures contributed to up-rootedness, limited maternal health care access, and absent societal support to women subjected to violence, which reinforced reliance on social networks, own endurance, and faith in the pre-migration setting. After migration, a wish to move forward in life was together with social cohesion, faith, and motherhood sources for well-being. Lawful rights for women were appreciated but could concurrently risk create power-tensions in close relationships. The midwife was more associated with medical care than with overall well-being or violence, but new societal resources were gradually incorporated with already known resources. Midwives were guided by experience-based knowledge and collegial support and strived for woman-centered approaches beyond ethnicity and culture in care encounters, with language, social gaps, and divergent views on violence as potential barriers in violence inquiry.

**Implications:** Pragmatism, a focus on “moving on,” and support from family and social networks constitute tools when handling violence and migration-related stressors, but need to be balanced against potential unspoken needs in care encounters. With trustful relationships, optimized interaction, and networking with local Somali communities and across professions, the antenatal care midwife can have a “bridging-function” in balancing acts between dual societies and contribute to enhanced health in the transition process in the new society.

## Social Justice and Its Role in Education, Training, and Implications to Practice for Nurses Caring for Victims of Violence

### Jacqueline Callari Robinson^1^ and Trisha Sheridan^2^

^1^University of Wisconsin–Milwaukee, Milwaukee, Wisconsin, USA

^2^Emory University, Atlanta, Georgia, USA

Nurses interact with patients across the spectrum of class and ethnicity in the provision of nursing care. However, the lack of diversity within the nursing workforce, the range of educational standards, along with discrimination, bias, prejudice, and lack of exposure to the consequences and trauma of social injustice may affect not only the nurse–patient experience, but the actual outcome of nursing care. Even with education of cultural competency, many nurses are resistant to the conversation that they individually and/or organizationally may perpetuate oppressive racial and socioeconomic practices that contribute to health disparity and discrimination. Even within the specialized field of forensic nursing, where there is a specific emphasis on trauma-informed patient-centered care, the acceptance of social injustice as a critical component of nursing care is not widely accepted nor exemplified. The future of nursing care to victims of violence must include a workforce that is educated in the traumatic effects of social injustice. Creating space for this discussion in a safe and respectful learning environment is essential for the future of the nursing profession, especially for those who care for victims of violence. Initial and continuing education on the principles of social justice including how to remove implicit bias must be part of a comprehensive and holistic approach to nursing care. This is imperative especially when dealing with those patients who seek care after violence has been perpetrated against them. This discussion will explore how to create the environment for the conversation, and design educational modules, tools, and competency standards for nurses in a variety of practice settings.

## The weWomen Adaptation of myPlan for Abused Immigrant Women

### Jacquelyn Campbell^1^, Bushra Sabri^1^, Joyell Arscott^1^, Veronica Njie-Carr^2^, Jill Messing^3^, and Nancy Glass^1^

^1^Johns Hopkins University School of Nursing, Baltimore, Maryland, USA

^2^University of Maryland School of Nursing, Baltimore, Maryland, USA

^3^Arizona State University, Phoenix, Arizona, USA

In the United States and elsewhere where studied, immigrant women have an increased risk of intimate partner violence (IPV) and of homicide by an intimate partner or ex-partner. The myPlan online and app-based IPV intervention and its adaptations have been shown to be a useful intervention for abused women in many countries and contexts. The weWomen study adapted the myPlan intervention for U.S. immigrant women using formative data from focus groups of 62 IPV service providers and 83 in-depth interviews of immigrant women survivors (Asian = 30, Latina = 30, African = 23) to contextualize the intervention for immigrant women. The data were first thematically analyzed for risk and protective factors and for suggested safety strategies to be included in the intervention. For instance, an important theme was extended family (primarily hers) as a protective factor while his extended family for some survivors was identified as an important risk factor. Therefore, her extended family was added as a priority in the priority selection part of myPlan and if highly valued, strategies about getting assistance from her extended family in the plan section. As studies and career were seldom indicated as a protective (or risk) factor, that priority was removed in the weWomen adaptation of myPlan. We also incorporated many of the suggested strategies for staying safe while staying in the relationship into the intervention plans, as neither our survivors nor our immigrant IPV service providers found leaving an abusive partner a viable option in most cases of abuse among immigrant women. The planning section also emphasizes the total confidentiality (from Immigration and Customs Enforcement [ICE] and other governmental agencies) and many language access of the National DV hotline/chatline (www.thehotline.org). The version of the Danger Assessment incorporated into the weWomen intervention was the DA-I, the previously developed Danger Assessment for Immigrant Women based on data on abused immigrant in the United States (www.dangerassessment.org). Thus, this adaptation of a well-tested existing intervention based on data from immigrant women and key informants currently being randomized controlled trial (RCT) tested will hopefully provide individualized assistance to abused immigrant women and strategies for adapting interventions for marginalized groups.

## Talking About “In the Kitchen Jokes”: Exploring the Impact of Structural Violence on Young Women and Men

### Eugenia Canas^1^ and Helene Berman^1^

^1^Western University, London, Ontario, Canada

Structural violence has been variously conceptualized as the institutionally permitted sanctioning of micro-aggressive gestures and overt discrimination. Due to the embedded nature of structural violence, its examination requires supporting individuals in connecting their daily, even embodied, felt experience with institutional-level policies and values. A critical emancipatory approach of this nature holds particular challenges when working with youth populations, particularly if they have previously been excluded from knowledge-generation, advocacy, or policy-making processes. A space to foster critical awareness and language among young men and women is needed. This presentation draws from work conducted under a national, 5-year Canadian Institutes for Health Research (CIHR) grant titled “Promoting Health Through Collaborative Engagement With Youth: Overcoming, Resisting, and Preventing Structural Violence.” In the course of this research, young women and men from across Canada (ages 16–24) participated in group discussions about their everyday lives, to identify how structural violence affects their well-being. Their insights—derived through diverse processes of art-making, and individual and group reflections—underscored the ubiquitous and permitted nature of structural violence, as well as its ability to affect them in gendered ways through the lifespan. In this presentation, we describe these discussions among young men and women of immigrant, LGBTQ+ (lesbian, gay, bisexual, transgender, and queer), and First Nations backgrounds as a process of consciousness-raising. Seeing and talking about structural violence together served to validate the complexity of these youths’ individual experiences, and find places for resistance through artistic expression, advocacy, and solidarity. In describing this research, we outline steps taken throughout the national grant and its associated projects to link youths’ experiences to organizational and policy-making agendas. Implications for researchers, community organizations, and policy makers will be provided, with a focus on creating supportive spaces for resistance and action.

## Measuring Gender Role Attitudes Among Chinese Women: Comparing the Gender Role Egalitarian Attitude Test With an Instrument for Traditional Gender Roles

### Jiepin Cao^1^, Susan Silva^1,2^, and Rosa Gonzalez-Guarda^1^

^1^Duke University, Durham, North Carolina, USA

^2^Duke University Medical Center, Durham, North Carolina, USA

Gender role attitudes, embedded in specific social and cultural contexts, have been identified to be a crucial factor shaping individual’s behavior relevant to intimate partner violence (IPV), including IPV victimization and perpetration. Gender roles in China are influenced by a long history of Confucius tradition where females are viewed as subordinate to males. However, China has experienced rapid social and economic development in the past decades, resulting in significant improvements in women’s social status. This change could potentially contribute to the shift toward more flexible and egalitarian gender roles between males and females. Although measurement tools have been created to capture gender role attitudes, there are no widely accepted tools available to measure this concept that captures gender dynamics within Chinese society. While two scales have been culturally tailored to the Chinese population, there are no psychometric data available on these for Chinese women so far. Thus, the purpose of this study is to assess psychometric performances of these two measurement tools, namely, the Gender Role Egalitarian Attitude Test and an Instrument for Traditional Gender Roles, among Chinese women. A cross-sectional design using survey data will be used. A total number of 250 Chinese women will be recruited from the largest crowdsourcing platform in China named zbj.com with 19 million registered users. The recruitment information for this study will be posted on zbj.com and registered users with interests in this study will be directed to the eligibility screening. For eligible participants, an online survey that includes demographic questions and these two tools will be administered after their informed consent. For each tool, construct validity will be assessed using confirmatory factor analysis (CFA) and the internal consistency of the entire scale and any subscales identified by the CFA will be evaluated with Cronbach’s alphas. The psychometric properties of the two tools measuring gender role attitudes among Chinese women will be compared, providing crucial information about their relative utility. The findings will provide valuable methodological information regarding the measurement of gender role attitudes in Chinese women, further informing future evaluation of intervention or prevention programs aiming to shift gender role attitudes in China.

## What’s in a Name? How the Advertised Study Title Effects Sexual Assault Self-Reporting

### Emily M. Carstens Namie^1^, RaeAnn E. Anderson^1^, and Paige Michel^1^

^1^University of North Dakota, Grand Forks, North Dakota, USA

Sexual assault (SA) affects approximately one in four college students. Prior researchers have noted that the context of studying SA may affect reported rates of SA and its correlates. National surveys on crime tend to find lower rates of SA than those studying health and the survey context of “questions about crime” has been cited as one possible explanation for this finding. We advertised four different studies in the psychology subject pool which appear to be about the topics of SA, health, crime, and alcohol, respectively, but all included the same set of questionnaires. We hypothesized that reported rates of sexual assault victimization (SAV) and sexual assault perpetration (SAP) would vary by study condition. Participants were 792 university undergraduate students. SAV was measured with the Sexual Experiences Survey–Short Form Victimization (SES-SFV), Conflict Tactics Scale–2 (CTS2), and the Childhood Trauma Questionnaire (CTQ). SAP was measured with the Sexual Experiences Survey–Short Form Perpetration (SES-SFP), and CTS2. Analyses of SAV measures revealed no differences in reports of SAV among type of study advertised on the SES-SFV and the CTS2. However, when measuring SAV with the CTQ, there were significantly more reports when the study was advertised as a SA study than one pertaining to crime or health but not when advertised as one researching alcohol use, χ^2^(3) = 12.456, *p* = .006. Analyses of SAP measures revealed no differences in reports of SA among type of study advertised. Secondary analyses investigating related correlates including personality and attitudinal factors are ongoing. The preliminary results of this study suggest that researchers have significant leeway in how studies are advertised such that, as long as data are collected anonymously, it will not adversely affect reported rates of sexual violence, except possibly when using the CTQ. Furthermore, this has ethical implications in that researchers do not need to be concerned that transparency will decrease data quality.

## Fostering a Trauma- and Violence-Informed Community: Developing Strategies to Inform Public Education

### Jessica Carswell^1^, C. Nadine Wathen^1,2^, Victoria Smye^1^, and Susan Macphail^3^

^1^Western University, London, Ontario, Canada

^2^Centre for Research & Education on Violence Against Women & Children, London, Ontario, Canada

^3^TVIC Educator & Consultant

Trauma and violence are pervasive public health issues. Social and systemic barriers intensify the effects of such experiences and negatively affect access to health and social services. Equity-oriented care practices combat these challenges, supporting health care settings to attend to and reduce the poor health outcomes associated with these experiences. Trauma- and violence-informed care (TVIC) is a key element of equity-oriented care, promoting the emotional, physical, and cultural safety of those accessing services—TVIC can be thought of as a “universal precaution,” minimizing harm in the care process. Work is being done to incorporate TVIC principles into organizational policies and practices, but little research has been done to translate this knowledge into information that informs communities and individuals accessing services. Therefore, we describe an ongoing research study that seeks to extend work that incorporates TVIC principles into organizational policies and practices. In collaboration with a community-based mental health organization in London, Ontario, Canada, this research utilizes qualitative methods to investigate how to use TVIC principles to frame and share TVIC information with the general community and individuals accessing services, and what barriers and facilitators may affect this process. Semi-structured interviews with staff and a document review of policies and procedures regarding public education and TVIC-related practices will help determine actions, priorities, and other relevant, equity-promoting considerations that are central to this investigation. The Exploration Stage of the Active Implementation Frameworks will be used to guide the data analysis and co-produce with the community partner effective recommendations for action. We will provide findings from the analysis to date and share our learnings that will inform recommendations for action at the community level. This Canadian research project is a critical first step in externally focused efforts to support broader community awareness and action regarding trauma, violence, equity, and cultural safety. This is important because social norms and structures can perpetuate the trauma, violence, and inequities experienced by vulnerable and marginalized groups—and inequities affect individuals, communities, and society.

## Exposure to Intimate Partner Violence Over 10 Years and Child Health Outcomes at Age 10: Findings From the Maternal Health Study

### Laura Conway^1^, Deirdre Gartland^1,2^, Stephanie Brown^1,2,3^, Fiona Mensah^1,2,3^, Sheena Reilly^1,2,4^, Emma Sciberras^1,5^, Rebecca Giallo^1,2^; the Maternal Health Study Team

^1^Murdoch Children’s Research Institute, Parkville, Victoria, Australia

^2^The University of Melbourne, Melbourne, Victoria, Australia

^3^Royal Children’s Hospital, Parkville, Victoria, Australia

^4^Griffith University, Gold Coast, Queensland, Australia

^5^Deakin University, Melbourne, Victoria, Australia

**Problem Statement:** Childhood exposure to intimate partner violence (IPV) is highly prevalent and associated with poorer developmental outcomes in cross-sectional and refuge/shelter-based studies. Few studies have investigated the association in community samples, using comprehensive IPV measurement, across multiple domains of functioning, or by timing of IPV exposure.

**Purposes and/or Questions/Hypotheses:** To investigate the mental and physical health and development of children exposed to IPV in their first, fourth, and/or 10th year.

**Study Design:** Prospective pregnancy cohort of nulliparous women recruited at ≤24 weeks gestation, followed up to 10 years postpartum.

**Sample:** A total of 1,507 mothers and their first-born children.

**Data Collection Approach:** Mothers were recruited from six Australian public maternity hospitals, and completed questionnaires at multiple time-points, including the Composite Abuse Scale to measure IPV at 12 months, 4 years, and 10 years. At 10 years, 615 mothers and children completed child physical, cognitive, and mental health assessments in face-to-face interviews.

**Analysis:** Multivariable logistic regression was used to examine the association between exposure to IPV in the first 10 years and child 10 year outcomes. Regressions were repeated using a three-level IPV exposure variable to categorize no IPV, early life exposure (at 1 and/or 4 years), and current exposure (at 10 years ± early exposure).

**Results:** Children exposed to IPV in their first, fourth, and/or 10th year had higher odds of psychiatric diagnoses, emotional/behavioral difficulties, disordered language, asthma, and sleep problems than their peers. No differences were observed for cognitive functioning or weight. In some domains, differences were observed by timing of IPV exposure. Earlier exposure was associated with higher odds of disordered language (odds ratio [OR] = 2.5 [1.1, 5.3]) and asthma (OR = 2.9 [1.6, 5.1]). Current exposure was associated with higher odds of psychiatric diagnoses (OR = 2.5 [1.5, 4.2]), emotional/behavioral problems (OR = 2.3 [1.3, 4.6]), elevated blood pressure (OR = 2.4 [1.1, 5.4]), and sleep problems (OR = 3.0 [1.4, 6.6]).

**Implications:** The broad range of associations for IPV exposure during the first 10 years, and for current exposure in particular, demonstrates the urgency to improve identification and response to family violence for children.

## “Stealthing” and Other Forms of Coercive Condom Use Resistance: An Under-Examined Type of Violence Against Women

### Kelly Cue Davis^1^, Mitchell Kirwan^1^, Rachel Van Daalen^1^, and Nolan Eldridge^1^

^1^Arizona State University, Phoenix, Arizona, USA

Nonconsensual condom removal—also termed “stealthing”—has received increased international media attention recently. Stealthing—a situation in which a man agrees to use a condom, but then removes the condom before or during intercourse without his partner’s knowledge or consent—is a type of sexual assault in some countries. In addition, other forms of coercive condom use resistance, such as emotional manipulation, deception, condom sabotage, and physical force, have been identified but are under-examined. The present study used a cross-sectional online survey to investigate the factors associated with stealthing and other coercive condom use resistance tactics in a nationwide sample of 18- to 30-year-old men (*N* = 104) residing in the United States who reported a history of coercive condom use resistance. Participants completed survey measures assessing sexual aggression history, alcohol expectancies related to sexual aggression, and alcohol use during their most recent coercive condom use resistance event. More than 18% (*n* = 19) of the participants reported having engaged in stealthing at least once since the age of 14. Of the men who had engaged in stealthing, they reported engaging in this behavior an average of 3.95 times (*SD* = 4.50), with a range of 1 to 21 times (maximum possible). Logistic regression analyses indicated that after controlling for social desirability, men with a more severe sexual aggression history (odds ratio [OR] = 2.82) had significantly higher odds of having engaged in stealthing behavior; history of psychological and physical intimate partner violence was not significantly predictive of stealthing. Regarding all coercive condom use behaviors, participants with stronger beliefs that alcohol increases the likelihood of their perpetration of sexual aggression (OR = 2.20) were more likely to have consumed alcohol when using coercive condom use resistance, while participants with stronger beliefs that alcohol increases women’s vulnerability to sexual coercion (OR = 2.20) were more likely to have used coercive condom use resistance with partners who had been drinking alcohol. Findings suggest that prevention efforts focusing on stealthing and other forms of coercive condom use resistance could benefit from targeting sexually aggressive men and addressing their beliefs about alcohol use and sexual aggression.

## Testing the Acceptability and Feasibility of the Men With Conscience Sexual Violence Prevention Intervention in a Pilot-RCT at Two Universities in the Western Cape

### Tania de Villiers^1^ and Naeemah Abrahams^2^

^1^University of Cape Town, Cape Town, South Africa

^2^South African Medical Research Council, Cape Town, South Africa

**Background and Objectives:** Higher Education Institutions has been identified as communities at risk for sexual violence against women and girls globally. Prevention interventions in these settings are critical for addressing the key drivers of sexual violence. However, few prevention interventions targeting male students have been developed and tested in Sub-Sahara. The study aimed to test the acceptability and feasibility of the Men With Conscience (MWC) intervention in a pilot randomized controlled trial (RCT) at two university campuses in the Western Cape. This paper presents the findings of a pilot-RCT conducted to test the MWC intervention and provides evidence of its potential to address social norms and other key drivers of sexual violence, specifically in university settings.

**Method:** This study used an open pilot-RCT, which consisted of the following phases: Phase 1: Planning, preparation, and development of the pre- and post-intervention questionnaire; Phase 2: Implementation of the intervention; and Phase 3: Post-intervention assessment. A post-intervention questionnaire will be administered to both the control and intervention groups, to establish impact of the intervention after 6, and then after 12 months.

**Key Outcomes:** The following key outcomes for this study were anticipated:

1. The main outcome of this study is a South African–designed, context-specific sexual violence prevention intervention for use in South African university settings, ready to be tested in a fully powered RCT.
2. Research dissemination, that is, publications in peer-reviewed journals, report back to the university communities, and presentation at local and international conferences.
3. Opportunity for developing emerging researchers, that is, master’s and PhD studies.

**Conclusion:** The study shows evidence of initial change among the young men and the huge need to work within higher education settings. The MWC intervention is ready to be tested in a more rigorous, fully powered RCT.

## Rural–Urban Differences in Intimate Partner Violence–Related Emergency Department Visits: Implications for Prevention

### Nancy R. Downing^1^, Maria Perez-Patron^1^, Brandie D. Taylor^2^, and Nora Montalvo-Liendo^1^

^1^Texas A&M University, College Station, Texas, USA

^2^Temple University, Philadelphia, Pennsylvania, USA

**Problem Statement:** Rural communities’ lack of access to preventive services for persons experiencing intimate partner violence (IPV). Data regarding rural–urban disparities in IPV can inform policy recommendations aimed at improving health outcomes for persons exposed to IPV.

**Purpose:** The purpose of this study was to examine rural–urban differences in IPV-related emergency department (ED) visit prevalence in the United States. Differences by U.S. Census region, gender, and income were also analyzed.

**Study Design:** A retrospective analysis of National Emergency Department Sample (NEDS) data for 2009–2014 was conducted on prevalence of IPV-related ED visits. IPV-related visits were identified using the International Classification of Disease, Ninth Revision, Clinical Modification (ICD-9-CM) code E967.3 (“battering and other maltreatment by spouse or partner”).

**Sample:** Patients aged 15 to 64 with an E967.3 code were included in the analytic sample. Rurality was determined using the National Center for Health Statistics urban–rural classification scheme. Micropolitan and non-core areas were classified as rural. Large central metros, large fringe metros, and small metros were classified as non-rural. Census regions included Northeast, Midwest, South, and West.

**Analysis:** Frequencies and prevalence of IPV-related ED visits per 100,000 residents were calculated. Odds ratios for gender differences were calculated.

**Results:** There were 156,945 IPV-related ED visits, averaging 26,158 visits per year. Prevalence was higher in rural (13.8 and 16.7) versus non-rural areas (range = 9.5–13.2) in all regions except the Midwest. Prevalence was 13 times higher for women than men, although men had 2.5 higher odds (95% confidence interval [1.5, 4.2]) of dying in the hospital following an IPV-related ED visit. Sixty-four percent of patients resided in zip codes with median household income in the bottom half of distribution.

**Implications:** Lack of access to resources for persons experiencing IPV in rural areas can delay intervention. Violence may escalate until injuries are serious enough to require an ED visit. Lack of women’s health and mental health providers in rural areas to identify and address IPV might contribute to higher IPV-related ED visits. Males might be less likely to seek assistance for IPV until injuries are life-threatening. Findings support a need for resources to mitigate health impacts of IPV.

## Building an Intersectoral Network to Champion Supports for Trans Survivors of Sexual Assault: Survey Findings From Health and Community Leaders

### Janice Du Mont^1,2^, Sarah Daisy Kosa^1,3^, Megan Saad^1,3^, and Sheila Macdonald^1,3^

^1^Women’s College Hospital, Toronto, Ontario, Canada

^2^University of Toronto, Toronto, Ontario, Canada

^3^Ontario Network of Sexual Assault/Domestic Violence Treatment Centres, Toronto, Ontario, Canada

**Issue:** Sexual assault against transgender (trans) persons is a complex issue that requires the coordinated effort of health care and social service sectors to address. In collaboration with the Ontario Network of Sexual Assault/Domestic Violence Treatment Centres (SA/DVTCs), which comprise of hospital-based sexual violence treatment centers that provide comprehensive post-sexual assault care across Ontario, and Egale Canada, a national LGBTQ+ (lesbian, gay, bisexual, transgender, and queer) advocacy organization, we have been working to bring together health and community leaders in an Intersectoral Network of service providers to champion support services for trans survivors of sexual assault across Ontario, Canada’s most densely populated province.

**Method:** Guided by the Lifecycle Evolutionary Model for network development described by the National Collaborating Centre for Methods and Tools, we hosted meetings across Ontario with local trans and LGBTQ+ positive organizations and leaders from Ontario’s SA/DVTCs to discuss the potential of forming an Intersectoral Network. Informed by critical insights garnered at these meetings and working with an advisory committee of trans community members and their allies, we created and distributed an online survey to 30 leaders of local SA/DVTCs and 105 community representatives across Ontario (*n* = 105) who attended the aforementioned meetings.

**Results:** Results from the survey will identify priorities in the area of sexual violence against trans persons, help inform future cross-sector training initiatives, improve the understanding of barriers and facilitators to intersectoral collaboration in the field of sexual violence, and provide key insights into the motivations and expectations from health care and community leaders in forming longer-term partnerships across sectors for enhancing the continuum of care to trans survivors of violence.

**Implications:** Results from the survey will ultimately facilitate the formation of a province-wide Intersectoral Network that will ensure that organizations and service providers across Ontario offer up-to-date, appropriate, and sensitive care and support to trans survivors. Importantly, trans survivors of sexual assault will benefit from the enhanced response to their needs post-victimization. Finally, the Network holds the potential to enhance policy, research, advocacy, and professional practice around the topic of sexual violence against trans communities across Ontario.

## Results From a Novel Elder Abuse Nurse Examiner e-Learning Curriculum

### Janice Du Mont^1^, Sarah Daisy Kosa^1,2^, and Sheila Macdonald^1,2^

^1^Women’s College Hospital, Toronto, Ontario, Canada

^2^Ontario Network of Sexual Assault/Domestic Violence Treatment Centres, Toronto, Ontario, Canada

**Issue:** A Canadian population–based study estimated that almost one in 10 (8.2%) older adults in Canada experienced some form of abuse or neglect in 2014, which amounts to 766,247 older Canadians. Despite the prevalence of elder abuse, there is no standard provision of training to provide care for abused older adults at hospital-based violence treatment centers in Canada’s most densely populated province, Ontario. To address this gap, Dr. Janice Du Mont of Women’s College Research Institute together with the Ontario Network of Sexual Assault/Domestic Violence Treatment Centres (SA/DVTCs), developed an evidence-based in-person Elder Abuse Nurse Examiner Curriculum to build upon the skills and expertise of nurses who have undergone Sexual Assault Nurse Examiner (SANE) online training. Following a successful pilot, the training was adapted into an interactive online format, accessible to all nurses who have completed online SANE Training currently working across Ontario’s 35 SA/DVTCs.

**Purpose:** To share findings from an evaluation of this innovative digital educational initiative for SANEs working across sexual violence treatment centers focused on improving the care provided to older adults who have experienced abuse.

**Method:** The e-learning curriculum was developed using Storyline 360 by Articulate, an award-winning software for the design of interactive courses, and guided by research-based Principles of Multimedia Learning. Two external reviewers with extensive expertise in curriculum development and design reviewed the e-Learning Curriculum which was subsequently revised based on their feedback. The curriculum was launched on an online learning management system to approximately 300 SA/DVTC nurses across Ontario in June 2019.

**Evaluation:** The curriculum’s efficacy will be evaluated through a pre- and post-training questionnaire which will measure self-assessed changes in competence in providing elder abuse care before and after completing the e-Learning Curriculum. In addition, to more directly measure competence in a clinical setting, a clinical vignette was incorporated in the questionnaires. Supplementary feedback, through open-ended questions, will also be collected on the appropriateness of the e-Learning Curriculum and its delivery.

## An Evaluation of a Forensic Nursing e-Learning Curriculum on Trans-Affirming Care for Sexual Assault Survivors

### Janice Du Mont^1,2^, Sarah Daisy Kosa^1,3^, and Sheila Macdonald^3^

^1^Women’s College Hospital, Toronto, Ontario, Canada

^2^University of Toronto, Toronto, Ontario, Canada

^3^Ontario Network of Sexual Assault/Domestic Violence Treatment Centres, Toronto, Ontario, Canada

**Issue:** Across Ontario, Canada’s most densely populated province, there are 35 hospital-based Sexual Assault/Domestic Violence Treatment Centres (SA/DVTCs), which provide services, including emergency medical care, crisis intervention, documentation of injuries, collection of forensic evidence, medical follow-up, counseling and referral, and community referrals to those identifying as women and men, as well as children who have been sexually assaulted. In a recent survey of program managers and frontline nurses across all SA/DVTCs, a strong need was identified for further training in trans-specific care. Therefore, we collaborated with the Ontario Network of SA/DVTCs and Rainbow Health Ontario to develop, pilot, and evaluate a novel e-learning curriculum that builds upon and enhances an existing in-person training for Sexual Assault Nurse Examiners (SANEs) on providing appropriate care to transgender (trans) sexual assault survivors.

**Purpose:** To examine the effectiveness of an innovative e-learning educational tool in improving competence in the provision of care to trans survivors of sexual assault among SANEs working across Ontario’s sexual violence treatment centers.

**Method:** The e-learning curriculum enhances an existing in-person curriculum that was successfully piloted and evaluated with a small sample of nurses from across Ontario. The e-learning curriculum was informed by research-based Principles of Multimedia Learning described in the literature and developed using Storyline by Articulate 360, an innovative software program specially designed for the development of interactive digital training programs. The curriculum was reviewed by a Community Reference Group comprised of experts in trans health and violence. The curriculum was launched on an online learning management system to approximately 300 SA/DVTC nurses in August 2019.

**Evaluation:** Changes in level of knowledge and perceived competence on 31 skills-based competencies related to trans-affirming care will be evaluated using a pre- and post-training questionnaire design that includes the use of clinical vignettes. If the results suggest that the curriculum is effective, we will disseminate the online curriculum and evaluation findings among relevant academic, government, health, and community stakeholders.

## Sex Ratios at Birth in Australia According to Mother’s Country of Birth: A National Study of All 5,614,847 Live Births in 1997–2016

### Kristina Edvardsson^1^, Mary-Ann Davey^2^, Rhonda Powell^1^, and Anna Axmon^3^

^1^La Trobe University, Melbourne, Victoria, Australia

^2^Monash University, Melbourne, Victoria, Australia

^3^Lund University, Lund, Sweden

**Problem Statement:** Male-biased birth sex ratios may occur after prenatal sex selection due to son preference, and have been an issue in countries across Asia. In the last decade, evidence of male-biased sex ratios in some immigrant communities has emerged in Western high-income countries, including in the state of Victoria, Australia. However, it was not known whether sex ratios were male-biased throughout Australia or whether patterns were consistent across states and territories, and over time.

**Purpose:** To assess male-to-female (M/F) ratios at birth per mother’s country of birth for all live births in Australia 1997–2016, in total and by parity, and to investigate if any observed deviations were consistent across states and time periods.

**Study Design, Sample, and Data Collection Approach:** This population-based register study included all reported live births of at least 400 g birth weight or at least 20 weeks gestation in all of Australia’s eight states and mainland territories for the period 1997–2016 (*N* = 5,614,847).

**Analysis:** M/F ratios with 95% confidence intervals (CIs) were estimated using logistic regression with an intercept-only model. Ratios with a CI entirely outside the range of 1.04 to 1.06 were considered as different to the biological M/F ratio.

**Results:** M/F ratios were elevated for births to women born in China overall (M/F ratio 1.084, 95% CI [1.071, 1.097]) and at Parities 1 and 2 (P1: 1.086 [1.065, 1.108]; P2: 1.175 [1.120, 1.231]), and to women born in India at Parities 1 and 2 (P1: 1.086 [1.065, 1.107]; P2: 1.146 [1.090, 1.204]). M/F ratios were elevated for both groups in New South Wales and Victoria, for Chinese births in Australian Capital Territory, and Indian births in Western Australia, with M/F ratios in the range of 1.156 to 1.307. M/F ratios remained elevated at Parity 2 across time periods for Chinese births, while a decline was observed for Indian births in the last 5-year period.

**Implications:** These findings confirm sex imbalances at birth among infants of mothers who have migrated from countries where son preference is manifest and M/F ratios elevated at national levels, and may indicate an unrecognized aspect of discrimination against females in Australia.

## Exploring Authentic Empathy and Male Victim Stigma Management in the Practice Narratives of Victim Service Providers

### Chuka Nestor Emezue^1^

^1^University of Missouri, Columbia, Missouri, USA

In spite of hegemonic masculine notions that stereotype male victims of sexual violence are weak, unmanly, or deviants of heteronormativity, anecdotal, research, and media evidence indicate more men are disclosing sexual assault to trusted members of their social support system—including victim service providers (VSP). We identify VSPs as those who provide direct or indirect rehabilitative services (not limited to health, legal, criminal, and counseling services) for male victims of sexual violence. Few studies consider the roles VSPs play in the context of stigma management with male victims. The purpose of this study is to explore VSP stigma awareness, stigma discourse, and stigma management practices (i.e., stigmatization, non-stigmatization, or de-stigmatization).

**Method:** Utilizing narrative inquiry methods (reflexive and discursive collaboration between the author and narrator), 11 VSPs (8 females and 2 males, age = 29–53 years) across U.S. states, representing different disciplines were interviewed using face-to-face and online modalities that lasted 45 to 90 minutes. Interviews were conducted in English, recorded, transcribed verbatim, and analyzed using thematic analysis guided by a constructivist–interpretivist paradigm. Participants were consented and uncompensated. The University of Missouri Institutional Review Board (IRB) approved this study.

**Results:** First, regardless of discipline, VSPs converged on their narratives of male stigma typologies and stigma management processes by narrating (a) stigma in the discursive sense and (b) stigma in the material sense (sociocultural and social-structural systems). Second, VSPs diverged in their nomenclature and philosophy of care by using labels like victims, survivors, targets, patients, clients, warriors, and thrivers to describe those they work with. Third, this study introduces the sensitizing concept of Authentic Empathy (AE)—the aptitude to genuinely experience shared compassion, informed by past membership in a stigmatized group, or a well-earned involvement with members of the same group. This study highlights important implications; specifically, the need for stigma-informed care (in addition to current theoretical and paradigmatic models) for highly stigmatized groups, the place of AE in stigma management, and the role of the discursive-materialist conception of stigma in future research and praxis.

## Immigrant Male Batterers: A Systematic Review of Treatment Gaps, Therapeutic Disparities, and Theoretical Misfirings in Intervention Programs

### Chuka Emezue^1^

^1^University of Missouri, Columbia, Missouri, USA

Batterer Intervention Programs (BIPs) show minimal evidence of treatment efficacy in curbing post-intervention recidivism. These interventions offer even less significant treatment potential for Immigrant Male Batterers (IMB), who contend with pre- and post-migration barriers to prevention and treatment in BIPs. Accordingly, best practices and treatment components of BIPs with promising results among IMB are inconclusive. Vital treatment components of promising BIPs and entry points for treatment uptake are discussed. A comprehensive literature search for quantitative and qualitative outcomes/intervention studies was conducted in 10 electronic databases based on these inclusion criteria: published between 1990 and 2018, interventional and observational/non-interventional studies targeting IMB, compared the effects of a new or modified curricula with standard curricula, and studies with immigrant versus non-immigrant group comparison. Study participants were mostly Hispanic men in the United States. Low to moderate intimate partner and domestic violence (IPV/DV) was frequently reported. Study outcomes were highly heterogeneous. Culturally specific programs included modified BIP curricula, and involved IMB in the subsequent iterative design of interventions, allowing for negotiation in cultural expressions of masculinity produced promising short-term results in changing IPV positive attitudes and increasing accountability in dyadic violence. However, long-term IPV/DV reductive effects remain inconclusive. Findings will have implications for culture congruent prevention and intervention program designed to address immigrant IPV outcomes, establish intervention entry points and areas of treatment divergence, uncover emergent contexts of immigrant-specific intimate partner violence (IPV), and recommend improvements to develop culturally differentiated IPV intervention protocol.

## Rural Attitudes Toward Intimate Partner Violence

### Tracy A. Evanson^1^*, Shawnda Schroeder^1^, and Barbara Zust^2^*

^1^University of North Dakota, Grand Forks, North Dakota, USA

^2^Gustavus Adolphus College, St. Peter, Minnesota, USA

*Both Drs. Evanson and Zust are presenting authors at the conference.

**Background:** Although intimate partner violence (IPV) cuts across all demographic categories, including geographic residence, there are few studies that focus on rural populations. Studies indicate that rural women may be more vulnerable to abuse due to geographic isolation from resources. Many authors also suggest that residents in rural areas hold patriarchal attitudes and beliefs which favor both male domination and tolerance for IPV. However, little empirical evidence exists to confirm this.

**Purpose:** The purpose of this study is to explore the attitudes and beliefs about IPV among residents of rural communities in the United States, and compare these with non-rural residents.

**Method:** (This study will be conducted in Fall/Winter, 2019). Subsets of questions from the National Community Attitudes Towards Violence Against Women Survey (NCAS), will be adopted into an electronic survey. The NCAS is a nationwide telephone survey that has been used in Australia for many years and includes multiple subsets of questions on various types of violence against women. For the purposes of this study, the subsets of questions pertaining to “Domestic Violence” and “Gender Equality” will be adopted into an electronic survey. Snowball sampling will be used to distribute an invitation to participate and survey link, initially through various community organizations and churches in both rural and non-rural communities in two upper Midwest states in the United States, and participants will be asked to pass the survey link on to others they know. Data will be analyzed utilizing SPSS. Researchers will use descriptive statistics, cross tabulations, and comparison of means tests (*p* < .05) to measure variation between demographic categories and perceived attitudes toward violence against women.

**Implications:** The results of this study will provide valuable empirical information about the attitudes and beliefs toward IPV among rural residents. These results have the potential to inform both researchers and service providers about issues that rural victims of IPV may face in their communities, and may help provide direction for community-wide IPV education and stigma-reduction in rural communities.

## Definitions and Data on Femicide

### Cristina Fabre^1^ and Cathrine H. Bärtel^1^

^1^European Institute for Gender Equality (EIGE), Vilnius, Lithuania

The definition of femicide is often considered to be “the killing of women and girls because they are female.” What separates the killing of women from a case of femicide is the gender-based motivation. However, the definition of femicide continues to be discussed, and none of the European Union Member States (MS) has codified it. An additional obstacle to combat femicide is the lack of administrative data available. In Europe, only 17 MS collect data and provide insight into the killing of women by their intimate partners, which demonstrates a key issue of data collection and risk assessment on femicide. Collecting data related to the victim–perpetrator relationship in conjunction with homicide data including variables such as motive or context is lacking across Europe. Risk assessment procedures and risk management strategies could efficiently protect women victims of intimate partner violence and prevent their revictimization and even their killing. The European Institute for Gender Equality (EIGE) has developed a Risk Assessment Guide based on some overarching principles: victims-centered and victims’ safety approach, gender and intersectionality approach, and child sensitive. It identified the most relevant risk factors, which can include fear of the perpetrator, recent separation or estrangement, disputes on child custody, pregnancy, stalking, and coercive behavior. The purpose of the identification of risk factors is to feed into effective risk management strategies, specifically with a multi-agency approach including police as well as the health sector, as it is especially underrepresented when it comes to data collection on the issue, despite it, often, being the initial institutional body to get in contact with the victim. Currently, EIGE is working on a new study, on femicide and administrative data. The study will act as a reference tool for ensuring a standardized categorization of femicide and improve data collection on violence against women in the area. These standardizations will be based on an evidence-based report, which will include police and medical-forensic evidence that reveals the “gender-based motives” of femicide. With the study, EIGE seeks to strengthen MS responses to combating intimate partner violence and acquiring a deepened understanding on the issue of femicide.

## The Ethical Dilemma of Mandatory Reporting Laws in the United States: Observations of a Harm Reduction Outreach Nurse

### Sarah Febres-Cordero^1^ and Kylie Smith^1^

^1^Emory University, Atlanta, Georgia, USA

**Problem:** Providing care to women who inject drugs (WWID) and are pregnant without reporting fetal abuse is against the law in 25 states in the United States. In addition, many states consider prenatal drug use a form of neglect which requires reporting. Nurses who implement prenatal health care to WWID in these states are in jeopardy of losing their licenses and being charged with a misdemeanor offense if they do not report suspected maternal drug use. At the same time, WWID and their fetuses are at risk of multiple harms during and after pregnancy, such as trauma, withdrawal, and disenfranchised grief.

**Purpose:** To provide routine prenatal and postpartum care for WWID through outreach in the southeastern United States.

**Innovation:** Syringe exchange outreach programs are the most probable place in the United States for a nurse to encounter WWID during pregnancy. Working with linkage specialists, we have designed a tailored pregnancy packet for expectant women encountered by a wound care nurse during syringe exchange outreach. Nurses in outreach build trusting relationships that provide an ideal environment for providing resources for prenatal and postpartum care which could reduce harm to mothers and their fetuses.

**Implications:** Observing WWID in a southeastern state in an outreach setting, it is clear they are fearful of approaching nurses as they are aware of the nurse’s duty to report neonatal abuse. WWID voice their concern of nurses reporting to child protection agencies resulting in loss of their newborn. Nurses who choose to work in harm reduction settings in mandatory reporting states are faced with few options when providing care. Nurses must either knowingly violate the law or report injecting drug use during pregnancy and lose credibility and trust. By providing tailored resources, nurses are less conflicted about the ethical dilemma of duty to report as they feel they have fulfilled their role in promoting health and providing access to care. However, this situation demonstrates that there is no clear solution to this structural violence, which highlights the ethical dilemmas that nurses encounter when political agendas affect health care.

## Family Violence Reform in Victoria, Australia: How Mental Health Services Manage Systemic Change

### Sabin Fernbacher^1^

^1^Sabin Fernbacher Consulting

The Victorian Royal Commission Into Family Violence 2016 saw an unprecedented focus on violence against women and children in Australia. Accepting all 227 recommendations, the Victorian Government set a new agenda for sweeping reforms and progressed immediately to implementation. The commission’s report highlighted systemic gaps, and subsequent recommendations addressed those gaps through a variety of strategies from policy to new service types and shared responsibilities. Sweeping changes include a multi-agency risk assessment framework, establishment of multi-agency support, support and safety hubs, and legislative reform. Policy development, the need to align state government and organizational policies with the new risk assessment framework, practice guidance and workforce development are part of this far-reaching reform. The commission highlighted the unique position of health and mental health professionals to identify and respond to family violence. It recognized the need for mental health services to get better at identifying and responding to family violence. Victorian mental health services have lacked clarity about their role and responsibilities regarding family violence. The commission’s recommendations included the need for a Chief Psychiatrist Guideline on Family Violence. It recommended establishment of Family Violence Advisers for mental health services to increase the capabilities of this sector. The published chief psychiatrist guideline went beyond its remit and includes practice guidance and organizational change strategies, and clearly outlines organizational responsibilities for this change. It aimed to assist mental health services to align their organizations within this new environment and take clear responsibility for family violence. While the Chief Psychiatrist guideline and practice guide outlines what ought to occur, the advisers are in a position to support some of the changes toward increasing organizational capabilities. The author of the Chief Psychiatrist Guideline has engaged with those responsible for its implementation to seek feedback about its practical application, including challenges. This presentation will look at the translation of those new policies into practice and the role of the Family Violence Advisers. Gains and challenges regarding this implementation will also be discussed.

## Birth Outcomes in a Swedish Population of Women Reporting a History of Violence Including Domestic Violence During Pregnancy: A Longitudinal Cohort Study

### Hafrún Finnbogadóttir^1^, Kathleen Baird^2^, and Li Thies-Lagergren^3^

^1^Malmö University, Malmö, Sweden

^2^Griffith University, Gold Coast, Queensland, Australia

^3^Lund University, Lund, Sweden

**Problem Statement:** Victimization of women is encountered in all countries across the world; it damages the mental and physical health of women. During pregnancy and the postpartum period, women are at a greater risk of experiencing violence from an intimate partner. The purpose of this study was to explore childbirth outcomes in a Swedish population of women reporting a history of violence including domestic violence during pregnancy.

**Method:** A longitudinal cohort design was utilized. In total, 1,939 pregnant women ≥18 years were recruited to answer two questionnaires, during early and late pregnancy. The available dataset included birth records of 1,694 mothers who gave birth between June 2012 and April 2014. Statistical analyses included descriptive statistics, *t* test, and bivariate logistic regression.

**Results:** Of 1,694 mothers, 38.7% (*n* = 656) reported a history of violence and 2% (*n* = 34) also experienced domestic violence during pregnancy. Women who were single, were living apart from their partner, were unemployed, had financial distress, and were smokers were at a higher risk of experiencing violence (*p* = .001). They also had significant low scores on the SOC-scale and high EDS-scores ≥ 13 (*p* = .001) when compared with women without a history of violence (*p* = .001). Having a history of violence increased a woman’s risk of having cesarean section (odds ratio [OR] = 1.33, 95% confidence interval [CI] [1.02, 1.70]). Likewise, a history of emotional abuse significantly increased the risk of having a cesarean section irrespective of whether it was planned or an emergency cesarean section (OR 1.50, 95% CI [1.09, 2.06]). Infants born by a mother who reported a history of violence were at significant risk of being born premature < 37 weeks of gestation compared with infants born by mothers with no history of violence (*p* = .049).

**Conclusion/Implications:** A history of violence and exclusively a history of emotional abuse has a negative impact on childbirth outcomes, including cesarean section and premature birth. Therefore, it is crucial with early identification of a history of violence or ongoing abuse in pregnancy to provide with support, which may have positive impact on birth outcome.

## Midwives’ Experiences to Meet Pregnant Women Who Are Exposed to Intimate Partner Violence at Prenatal Ward

### Hafrún Finnbogadóttir^1^, Ella Torkelsson^2^, Cecilia Barna Christensen^2^, and Eva-Kristin Persson^3^

^1^Malmö University, Malmö, Sweden

^2^Malmö University Hospital, Malmö, Sweden

^3^Lund University, Lund, Sweden

**Problem Statement:** Worldwide every third woman is exposed to physical and/or sexual violence, and pregnancy is no safe period for the women. The aim was to elucidate midwives experience of “violence-exposed pregnant women” who are referred to the prenatal department and hospitalized.

**Method:** An inductive qualitative method was chosen. The data collection was performed with focus group interviews with 16 midwives divided into four groups. The inclusions criteria were midwives who had clinical experience at prenatal department for at least 1 year. The context was midwives working at one of four prenatal departments with different geographical areas in southern Sweden. The data analysis was performed using content analysis.

**Preliminary Results:** Three categories emerged from the data “Professional area of responsibility” were the midwives at prenatal department working at in-hospital care considered that it was the responsibility of the midwives working at antenatal care to “ask on routine” and disclose violence-exposed women. That a sign of help-seeking was based on the pregnant woman’s behavior by several visits to the health care. Also that the midwives suspicion of intimate partner violence was based on gut feeling which arose because of the woman’s or the partners behavior. “Conditions for support” comprehends that the midwives strived for supporting pregnant women who already were identified as violence exposed or if they had a suspicion that the pregnant woman was having a relationship where intimate partner violence occurred. “Barriers for giving support” were both the workplace conformation and routines that constituted a barrier. Also, the midwives’ own emotional state could affect the handling of the situation in the engagement with the violence-exposed woman.

**Conclusions/Implications:** The result showed that the midwives working in hospital care considered it was primarily the responsibility of midwives at antenatal health care to “ask on routine” and disclose violence-exposed pregnant women. The midwives had limited experience in dealing with violence-exposed pregnant women, but identified a number of signs and symptoms that could cause suspicion. They felt miserable in the situation and expressed need of both education and a plan of action.

## Burndawan: The Co-Design, Development, and Launch of an Online Indigenous Family Violence Resource

### Renee Fiolet^1^, Laura Tarzia^1,2^, Renee Owen^3,4^, Syd Fry^3^, Kaley Nicholson^3^, Corrina Eccles^3^, Jasmin Knox^3^, May Owen^3^, and Kelsey Hegarty^1,2^

^1^The University of Melbourne, Melbourne, Victoria, Australia

^2^The Royal Women’s Hospital, Melbourne, Victoria, Australia

^3^Wadawurrung Aboriginal Advisory Group

^4^Barwon Health, Geelong, Victoria, Australia

**Problem Statement:** Across the globe, Indigenous populations experience higher rates of family violence than non-Indigenous populations, yet are less likely to access support. Over recent years, there has been evidence to suggest that online resources to address violence within intimate relationships can be supportive and motivational. However, until now, no online resources addressing family violence have been co-designed with an Indigenous community solely for the purpose of it being meaningful and useful for their own peoples.

**Purpose:** To co-design an online family violence intervention with the Indigenous peoples of Wadawurrung Country (Australia) for use by their own peoples.

**Study Design:** This co-design study for a doctoral thesis involves qualitative methods that were guided by an Aboriginal community advisory group. These methods involved semi-structured interviews, focus groups, community verification methods, and pilot testing of the resource.

**Sample and Data Collection Approach:** Six Aboriginal community advisory group members guided the process of 23 interviews and five focus groups. Aboriginal advisory group consultations also included analysis of findings which informed content and design decision making.

**Analysis:** An Indigenous lens was applied to the thematic analysis through engagement of the Aboriginal Advisory Group.

**Results:** Community co-design led to three modules that the community felt would be necessary for an informative and supportive online resource. The first module “Assess” offers users the opportunity to assess their relationships and prioritize their needs. The second module “Act” encourages users to pull together a plan of action for managing their situation and provides access to helpful Indigenous and non-Indigenous resources. The third module is “heal” and incorporates elements that promote Indigenous social and emotional well-being such as connection to culture and mob.

**Implications:** Community co-design in the online resource has led to a resource which is relevant and culturally appropriate for the population intended.

## You’re a Whore, but You’re Also a Black Whore: Indigenous Experiences of Family Violence

### Renee Fiolet^1^, Laura Tarzia^1,2^, Renee Owen^3,4^, Syd Fry^3^, Kaley Nicholson^3^, Corrina Eccles^3^, Jasmin Knox^3^, May Owen^3^, and Kelsey Hegarty^1,2^

^1^The University of Melbourne, Melbourne, Victoria, Australia

^2^The Royal Women’s Hospital, Melbourne, Victoria, Australia

^3^Wadawurrung Aboriginal Advisory Group

^4^Barwon Health, Geelong, Victoria, Australia

**Problem Statement:** Indigenous peoples experience family violence at much higher rates than non-Indigenous peoples and the impact of the abuse is said to be significantly more burdensome. Despite this, there is currently limited understanding about how race affects the experience of family violence and the processing of abuse.

**Purpose:** To explore how race influences the experience and comprehension of family violence for Australia’s Indigenous peoples.

**Study Design:** Qualitative—semi-structured interviews and focus groups.

**Sample and Data Collection Approach:** Twenty-three interviews and five focus groups with Indigenous men and women from Wadawurrung Country, Australia.

**Analysis:** Initial coding and consultation with the Aboriginal community advisory group have led to the development of two preliminary themes.

**Results:** Two main preliminary themes were developed. The first, “You’re a whore, but you’re a black whore” describes how non-Indigenous perpetrators of family violence use race as a weapon in abuse. The second theme “A black fella thing” speaks to how racist community assumptions affect Indigenous peoples’ processing of family violence. Indigenous peoples felt that the violence in their families and community was only reproducing what society was telling them about Aboriginal culture.

**Implications:** Race can be used as a weapon in family violence and influences the experience of it. Further research into how service providers can address race in their response to family violence is required.

## Physical and Emotional Intimate Partner Violence and Women’s Mental, Physical, and Sexual Health After Childbirth: An Australian Pregnancy Cohort Study

### Kelly Fitzpatrick^1,2^, Stephanie Brown^1,2^, Kelsey Hegarty^2,3^, Fiona Mensah^1,2,4^, and Deirdre Gartland^1^

^1^Murdoch Children’s Research Institute, Melbourne, Victoria, Australia

^2^The University of Melbourne, Melbourne, Victoria, Australia

^3^The Royal Women’s Hospital, Melbourne, Victoria, Australia

^4^Royal Children’s Hospital, Melbourne, Victoria, Australia

**Problem:** Despite increasing recognition that women experience different types of intimate partner violence (IPV), much research has focused on physical IPV. There is limited evidence of the health impacts of different types of IPV.

**Purpose:** To explore women’s experiences of emotional, physical, or concurrent emotional and physical IPV, and to examine associations with women’s mental, physical, and sexual health in the first 12 months postpartum.

**Study Design:** Prospective pregnancy cohort study of nulliparous women recruited at ≤ 24 weeks gestation.

**Sample:** A total of 1,346 first-time mothers.

**Data Collection:** Women were recruited from public hospitals in Melbourne, Australia. Follow-up in pregnancy, 3, 6, 9, and 12 months postpartum. Past year IPV was measured with the Composite Abuse Scale at 12 months postpartum. Health outcomes assessed over the same period include depressive symptoms (Edinburgh Postnatal Depression Scale), anxiety, general health (Short Form Health Survey), incontinence, resumption of sex, body mass index, and body image.

**Analysis:** Multivariable logistic regression adjusting for maternal age and mode of birth.

**Results:** One in six women reported IPV in the first year postpartum (17.4%, *n* = 234). Emotional IPV alone was most common (9.5%, *n* = 128), followed by both physical and emotional IPV (5.7%, *n* = 76). Physical IPV alone was uncommon (2.2%, *n* = 30). Experience of physical and emotional IPV was most strongly associated with depressive symptoms (adjusted odds ratio [OR] = 4.6, 95% confidence interval [CI] [2.9, 7.1]) and self-reported anxiety (adjusted OR = 2.9, 95% CI [1.9, 4.4]). Experience of emotional IPV alone was associated with poor mental health as well as physical factors, including poor general physical health (adjusted OR = 1.9, 95% CI [1.2, 3.1]) and pain during sex (adjusted OR = 1.8, 95% CI [1.2, 2.7]). Women reporting emotional IPV alone and physical and emotional IPV also had increased odds of poor body image.

**Implications:** Frequent contact with primary health care in the perinatal period provides a unique opportunity to support women experiencing IPV. Our findings demonstrate the need for health care services to be aware of the prevalence of different types of IPV, and the range of health problems that are more common among women experiencing emotional IPV alone as well as women also experiencing physical IPV.

## Attending to Context and Complexity: Evolving a Promising Health Promotion Intervention for Women Separating From an Abusive Partner

### Marilyn Ford-Gilboe^1^, Colleen Varcoe^2^, and Kelly Scott Storey^3^; for the iHEAL Team

^1^Western University, London, Ontario, Canada

^2^University of British Columbia, Vancouver, British Columbia, Canada

^3^University of New Brunswick, Fredericton, New Brunswick, Canada

The negative effects of intimate partner violence (IPV) are broad, linked, and often continue after separation, affecting women’s safety, mental and physical health, social relationships, economic situation, and parenting. Women’s differing needs, priorities, resources, and living conditions affect how they seek help and the types of support that might be helpful. As such, comprehensive interventions that consider the context and complexity of women’s lives and are tailored to their unique circumstances, priorities, and needs are most likely to show benefits. These types of interventions are congruent with relational nursing practice approaches and can provide a way of operationalizing research evidence and theory, including concepts such as Trauma- and Violence-Informed Care (TVIC). Importantly, evaluations of “complex” interventions should retain a focus on complexity and context; that is, they should be designed to not only capture more than group differences on main outcomes, but also account for who benefits, how, and why. However, few such nursing interventions have been developed and tested, particularly in the context of separating from an abusive partner.

To address these gaps, we developed iHEAL, a comprehensive, trauma- and violence-informed intervention for women who are in the transition of separating from an abusive partner. Supported by a Clinical Supervisor, community health nurses, who have completed standardized iHEAL Education, work in partnership with women for ~6 month (10–18 sessions) to address a broad range of issues that affect women’s safety, health, and well-being. Tailoring the intervention to the individual woman’s priorities, needs, and context, and to the community in which she lives, is a key feature that could enhance successful integration into different service settings. iHEAL is woman-led, with a strong focus on complementing and extending, rather than duplicating, existing services. It is flexible enough to fit the needs of all women, with potential to reduce inequities.

In three separate studies (including one with Indigenous women), women found iHEAL safe and acceptable; substantial improvements in women’s health and quality of life were maintained 6 months after iHEAL ended. We are now examining the effectiveness and cost-effectiveness of a revised version of iHEAL in a randomized controlled trial of 331 Canadian women. We are also exploring who benefits from iHEAL and why, how iHEAL education and experience shape the nurses’ practice, and the conditions needed to support broader scale-up if effective. Intervention delivery ends in February 2020; 18-month follow-up assessments continue until March 2021.

**Aims:** In this session, we draw on over two decades of research, including baseline data from our current randomized controlled trial (RCT) to

- describe the theoretical and empirical foundations of iHEAL and its’ unique delivery model, summarizing key lessons from research to date
- illustrate the principles, strategies, and processes used to develop, test, adapt, and evolve iHEAL in ways that attend to complexity and context
- explore how insights from this research could strengthen interventions and services for women experiencing IPV in different contexts

**Content Outline:** This interactive session will include four brief presentations focused on (a) initial development of iHEAL and adaptation for Indigenous women, and key results and “lessons” from the three initial studies; (b) an overview of the current version of iHEAL, including expanded theoretical grounding to incorporate relational practice and TVIC; revised principles, components, and delivery model; and resources to support intervention delivery; (c) the design of the current RCT as an exemplar of benefits and challenges of adopting emerging principles for evaluating complex interventions; and (d) considerations for adapting complex IPV interventions to other contexts, drawing on current work to adapt iHEAL for Francophone women.

**Format:** Participants will be invited to consider and discuss how “lessons learned” apply to their own methodological and intervention work. Videoclips of iHEAL participants and nurses and examples of training and intervention materials will be used for illustration.

## Gender Matters: Testing the Composite Abuse Scale (Revised)–Short Form With a Sample of Canadian Adults

### Marilyn Ford-Gilboe^1^, Nadine Wathen^1^, Nancy Perrin^2^, Sue O’Donnell^3^, Kelly Scott-Storey^3^, Colleen Varcoe^4^, Jenn McGregor^1^, Alice Pearl Sedziafa^1^, and Joanne Hammerton^1^

^1^Western University, London, Ontario, Canada

^2^Johns Hopkins University School of Nursing, Baltimore, Maryland, USA

^3^University of New Brunswick, Fredericton, New Brunswick, Canada

^4^University of British Columbia, Vancouver, British Columbia, Canada

**Background:** Intimate partner violence (IPV) is a gendered experience, yet limited attention has been given to developing brief self-report measures capable of capturing gendered patterns of IPV. We developed the 15-item Composite Abuse Scale, revised–Short Form (CASR-SF) to measure severity and patterns of IPV in population surveys and general IPV studies. Using data from 6,278 Canadian women, the CASR-SF reliably captured different patterns of IPV that were variously associated with health and social outcomes. A survey of experts, and cognitive testing of the CASR-SF with 31 men who were experiencing IPV provided initial support for its fit and applicability for men. This study was conducted to further assess the psychometric properties of the CASR-SF among Canadian adults, with a focus on identifying gender-specific patterns of IPV captured by this measure.

**Method:** A community sample of 1,100 Canadian adults (500 men, 500 women, 100 people with non-binary identities) with histories of recent IPV was recruited using online advertisements. Participants completed an online survey hosted on a secure website comprised of the CASR-SF and measures selected to examine its concurrent validity: CESD-R (symptoms of depression), PCL-C (symptoms of post-traumatic stress), and WEB (experiences of coercive control). Data on gender identity of the victim and abusive (ex)partner(s) were collected, and perceptions of “fear” of partner explored.

**Results:** In all, 507 participants have completed the survey to date (completion is expected by December 2019). Preliminary results suggest that there are important gender differences at the item level and with respect to the reasons for reporting fear of partner. Statistical analyses will replicate those used in the original study to assess the factor structure, internal consistency, and concurrent validity of the scale with women but adopting a gendered approach. Patterns of IPV among people of different genders and in different types of partner relationships will be identified using cluster analysis.

**Conclusion:** The ability to accurately measure IPV among adults of all genders and to identify gendered patterns of abuse is needed as a foundation for developing a more nuanced understanding about the nature and impacts of IPV in different groups. This knowledge may ultimately inform the development of gender-sensitive services and policies that better support the needs of different groups of people living with IPV.

## Promoting Healthy Relationship Formation Among Young Adolescents in the Rio Grande Valley

### Nina Fredland^1^ and Elif Isik^1^

^1^Texas Woman’s University, Houston, Texas, USA

One in four women experience intimate partner violence (IPV) and 7 million children live in homes where IPV occurs. Such exposure interferes with healthy development and healthy relationship formation. These youth are at increased risk for negative outcomes because their mothers experienced IPV. There is evidence that arts-based interventions are enjoyed by youth and have a positive impact on attitudes and potentially can change behavior.

**Design:** This primary prevention program is designed to help a population of selected youth gain insight and skills to deal with difficult situations, while curbing unhealthy teasing/bullying and dating behaviors. Specifically, this study explores the feasibility of replicating an interactive theater intervention conducted with youth in Central Texas, with a selected sample of youth known to be exposed to IPV.

**Sample/Setting:** Nine Hispanic children, aged 11 to 15 living in the Rio Grande Valley, whose mothers sought assistance from a domestic violence advocacy group participated in 90-minute interactive performances over 4 consecutive days. Data were collected at baseline, immediately post intervention, and 3 months later.

**Analysis:** Spearman’s rho analyses examined the correlation among baseline outcomes. Wilcoxon signed rank and McNemar tests examined changes on variables from baseline to Time 2 and Time 3.

**Results:** At baseline, greater violence exposure was highly related to greater community violence; greater home/family violence was highly related to less knowledge; greater personal victimization was moderately highly related to greater teasing/bullying victimization; higher scores of total normative beliefs about aggression was highly related to higher coping. Youth reported significantly better knowledge, normative beliefs about aggression, fewer emotional and behavioral problems, and less teasing/bullying immediately after and 3 months post intervention.

**Implications and Lessons Learned:** It is feasible to network and partner with a local theater group in a geographically removed location to develop and implement an interactive theater intervention aimed at reducing negative health consequences for children exposed to IPV. Children who are in underserved areas with limited resources will benefit from such programs. Prevention programs strategically targeting selected populations may be more effective in breaking the intergenerational cycle of violence.

## Intimate Partner Violence Across the First 10 Years of Mothering and Associated Mental and Physical Health Outcomes

### Deirdre Gartland^1,2^, Kelly Fitzpatrick^1^, Fiona Mensah^1,2^, and Stephanie Brown^1,2^

^1^Murdoch Children’s Research Institute, Melbourne, Victoria, Australia

^2^The University of Melbourne, Melbourne, Victoria, Australia

**Purpose:** To investigate intimate partner violence (IPV) and women’s mental and physical health in the 10 years after having their first child.

**Study Design:** Prospective pregnancy cohort of nulliparous women recruited at ≤24 weeks gestation and followed up to 10 years postpartum.

**Sample:** A total of 1,507 mothers and their first-born children.

**Data Collection:** Women were recruited from public hospitals in Melbourne, Australia. Follow-up in pregnancy, and 1, 4, and 10 years. IPV assessed with the Composite Abuse Scale at 1, 4, and 10 years postpartum. Health outcomes at 10 years included PHYSICAL (SF-36, weight and body image, fecal and urinary incontinence [UI], and common morbidities) and MENTAL (Centre for Epidemiological Studies Depression Scale, Beck Anxiety Inventory, Post-Traumatic Stress Disorder [PTSD] Checklist, use of psychotropic medications).

**Analyses:** Multiple imputation of missing data to account for selective attrition. Logistic regression.

**Results:** One in three women reported IPV over the first 10 years of motherhood (34%). The prevalence of IPV was consistent across time with one in five women reporting IPV at 1 year (19%), 4 years (21%), and 10 years (18%). Women reporting IPV had raised odds of all mental health outcomes including depressive symptoms (odds ratio [OR] = 2.9, 95% confidence interval [CI] [2.2, 4.0]), anxiety symptoms (OR = 3.3, 95% CI [2.2, 5.0]), and PTSD (OR = 4.0, 95% CI [2.9, 5.6]). Over half the women reporting depression (54%), anxiety (60%) or PTSD (62%) at 10 years had experienced IPV. Women experiencing IPV also had raised odds of poor physical health including general health, UI, being overweight/obese and, migraines.

**Implications:** IPV is at least as common as maternal depression and is associated with significant physical and psychological morbidity. More than half of women with mental health symptoms at 10 years postpartum had experienced IPV. Primary care and psychological services must be alert to the high likelihood of co-occurring exposure to IPV and a range of mental and physical health symptoms, and the need to tailor responses accordingly.

## Engaging With Uncertainty and Complexity: Primary Care Responses to Intimate Partner Violence

### Claire Gear^1^, Jane Koziol-McLain^1^, and Elizabeth Eppel^2^

^1^AUT University, Auckland, New Zealand

^2^Victoria University of Wellington, Wellington, New Zealand

**Background:** Complex problems generate uncertainty. The number and diversity of interactions between different parts of the problem makes predicting an outcome very difficult. With complex problems, a small intervention may lead to big effects, or a big intervention may lead to small or no effects. An intervention can also have unintended or unexpected effects. In effort to reduce the uncertainty of intimate partner violence interventions, health care systems have sought to standardize responses by developing guidelines and protocols. This paper challenges the prescriptive approach and demonstrates how engaging with uncertainty can improve individual and system-level primary care professional responsiveness to intimate partner violence. We explore the concept of uncertainty and consider implications for practice.

**Method:** This paper draws on a complexity-led discourse analysis of 17 interviews with New Zealand primary care professionals on intimate partner violence as a health issue. The emergent Triple R Pathway articulated the interactions between participant understanding of intimate partner violence, their conceptualized response, and consequently, their responsiveness to someone experiencing violence. We review these Triple R Pathways, focusing on the manifestations of uncertainty, how it is managed, and the effect on patterns of responsiveness.

**Discussion:** This paper argues against prescriptive standardized health care responses to intimate partner violence. We distinguish between the inherent uncertainty involved in responding to intimate partner violence and the common manifestation of doubt when faced with someone experiencing violence. We provide examples of how avoiding uncertainty constrains primary care professional responsiveness and call attention to strategies professionals use to engage with uncertainty. We consider the implications for improving practice and for informing future policies and protocols that promote sustainable health system responses to intimate partner violence.

## When a Longitudinal Study Becomes Trauma-Informed Care: The Experiences of Field Nurses

### Heidi Gilroy^1^ and Angeles Nava^2^

^1^Children’s Memorial Hermann Hospital, Houston, Texas, USA

^2^Texas Woman’s University, Houston, Texas, USA

To determine the effectiveness of criminal justice and safe shelter interventions for women who had experienced intimate partner violence, a study was funded to document mental health and functioning outcomes in 300 women for 7 years. Three researchers recruited women and then visited them every 4 months for 7 years. The women had the same researcher throughout the program. The researchers used a scripted tool to collect information. No intervention was given to the women in the study; however, the women reported behavior change as a result of the relationship developed from the brief interaction in each visit. We do not believe that the study results were affected by the behavioral changes because all women had the same experience of an assigned researcher with a scripted tool. We do believe that some lessons learned from this experience might help inform future longitudinal studies as well as advocacy interventions for women who have experienced intimate partner violence. The purpose of this presentation is to share those lessons learned with researchers and advocates.

## Acculturation, Acculturation Stress, Adverse Childhood Experiences, and Intimate Partner Violence Among Latinx Immigrants in the United States

### Rosa M. Gonzalez-Guarda^1^, Brian E. McCabe^2^, Miriam Quizhpilema Rodriguez^1^, and Richard C. Cervantes^2^

^1^Duke University School of Nursing, Durham, North Carolina, USA

^2^Auburn University, Auburn, Alabama, USA

^3^Behavioral Assessment Inc., Beverly Hills, California, USA

Recent Latinx (gender inclusive term for individuals of Latin American origin) immigrants report less exposure to intimate partner violence (IPV) than their more established counterparts. Research suggests that years in the United States and acculturation to American culture are risk factors for IPV victimization in this population. Nevertheless, little is known about the relative contribution of acculturation versus the stress associated with this process (i.e., acculturation stress). In addition, research exploring the influence of acculturation on IPV risks among Latinx immigrants has seldom considered the influence of exposures to other stressful experiences on IPV risk such as childhood and immigration-related adversity. The purpose of this presentation is to examine the relationships among acculturation, acculturation stress, adverse childhood experiences, and IPV among Latinx immigrants. Baseline data from a longitudinal study of 385 community-dwelling Latinx immigrants between the ages of 18 and 44 in the research triangle area of North Carolina were collected. Bilingual and bicultural researchers collected data in participants’ homes using standardized measures for acculturation (Bidimensional Acculturation Scale), acculturation stress (Hispanic Stress Inventory 2–Immigrant Version), adverse childhood experiences (ACE–International Questionnaire), and IPV victimization (Conflict Tactics Scale), which had previously been validated with Latinx immigrants. The majority of the sample were female and emigrated from Mexico to be with family or for financial opportunities. Multiple logistic regression controlling for age, gender, age of immigration, and education showed that acculturation stress was related to IPV victimization, but acculturation and adverse childhood experiences were not. In fact, participants with high levels of acculturation stress had about twice the odds of reporting IPV victimization. It appears that acculturation stress plays a role in increasing risk for IPV among Latinx immigrants as they adapt to American life. Interventions are needed to help minimize exposure to stress and improve coping skills during this critical period and to prevent the potential toxic effects that this has on conflict and violence in intimate relationships. Additional research is also needed to examine these relationships among Latinx immigrant subgroups, including subgroups based on country of origin, gender identity and sexual orientation, and geographical regions in the United States, among others.

## Learning From a Domestic Violence and Advocacy Model Within Primary Care, the Story of Identification and Referral to Improve Safety

### Melanie Goodway^1^, Annie Howell^1^, and Medina Johnson^2^

^1^IRISi, United Kingdom

^2^IRISi, Honorary Contract University of Bristol, Bristol, United Kingdom

IRISi works to promote and improve the health care response to gender-based violence. IRIS (Identification and Referral to Improve Safety) is our innovative, evidence-based, domestic violence and abuse (DVA) training, support, and referral program for general practice. Research shows that 80% of women in a violent relationship seek help from health services, and these are often a woman’s first, or only, point of contact. Therefore, we know a dedicated training and support package is vital to address DVA, aid prevention, and provide appropriate responses. Tested and positively evaluated in a randomized controlled trial, the IRIS program is recognized as the gold standard for supporting clinicians in general practice to recognize and respond to their patients affected by DVA. Women in IRIS trained practices are six times more likely to be referred to specialist support and 22 times more likely to have a conversation about DVA with their clinician. After training, clinicians report improved knowledge and skills around DVA, increased confidence to deal with and respond to disclosures and can assess immediate risk, knowing then where to refer patients for support. This training is an advance in education for primary health care professionals who often have little prior knowledge of the dynamics of DVA, the health impacts, and how to respond appropriately and safely to disclosures. We know that clinicians, practice teams, and patients benefit greatly from the IRIS program and would recommend it, with patients feeling safer, more able to cope, and reporting they visited their general practice less frequently, Since 2010, IRIS teams have fully trained practice teams in more than 850 general practices and directly supported more than 14,000 women. We expect that many more women will have had a conversation about DVA with their primary health care clinician and that many of these women will subsequently seek support. Our session will delve further into the evidence base we have for the importance of a health care response to DVA, sharing lessons learned and best practice for working with health care professionals, specifically within general practice and for nursing teams.

## Speaking Out About #FGM: Messages and Visuals on Twitter

### Jeanine P. D. Guidry^1^, Candace W. Burton^2^, Liza Ngenye^3^, Iona Coman^4^, and Kellie E. Carlyle^1^

^1^Virginia Commonwealth University, Richmond, Virginia, USA

^2^University of California–Irvine, Irvine, California, USA

^3^La Sierra University, Riverside, California, USA

^4^Texas Tech University, Lubbock, Texas, USA

**Problem:** Female genital mutilation (FGM) remains unfortunately common, affecting more than 200 million living women and girls worldwide, and prevention efforts have resulted in only small decreases in prevalence. It is thus critical to understand how and/or why FGM is valued among different populations. Social media can provide important insights in this area. To date, there have been no studies analyzing discussion surrounding FGM on social media. This study analyzed FGM-related Twitter posts using the Social Ecological Model (SEM).

**Research Questions:** What are the ways FGM-related messages on social media platform Twitter reflect public health understandings of, and approaches to, prevention? How do Twitter users engage with these posts?

**Study Design and Analysis:** This study uses a quantitative content analysis of 1,000 tweets using hashtags #FGM, #FemaleGenitalMutilation, and #EndFGM. The sample was collected in August 2019, using netlytic.org. Coding categories include engagement variables, consequences of FGM, cultural coercion, and SEM-related variables.

**Results:** Preliminary analysis of a random subsample of 150 tweets showed that a majority (70.7%) included a visual, and 12.7% mentioned specific consequences of FGM; 17.3% mentioned attribution for the cause of FGM; of those, 73.1% included individual, 26.9% interpersonal, 42.3% community level, and 30.8% societal/policy attributions. In all, 70.0% mentioned attribution for preventing or stopping FGM with 53.3% mentioning individual attribution, 8.6% interpersonal, 45.7% community level, and 60.0% societal/policy. Only 4.7% mentioned cultural coercion related to FGM, and 8.7% female objectification.

**Implications:** Social media offers remarkable opportunities to explore perceptions and considerations of diverse populations with regard to issues such as FGM. Although the practice of FGM is known to have adverse effects on the health of women and girls, it has proven difficult to eradicate. The results of this study offer new insights into how FGM is perceived and understood by a wide range of individuals. These insights can inform both preventive campaigns and strategies for addressing this issue in the context of patient care.

## Self-Esteem in the Context of Intimate Partner Violence: A Concept Analysis

### Ayse Guler^1^ and Carolyn R. Smith^1^

^1^University of Cincinnati, Cincinnati, Ohio, USA

**Background:** Intimate partner violence (IPV) is defined as assaultive and coercive behaviors by a perpetrator to control a current or former intimate partner. Of women in the United States, an estimated 48% experience psychological IPV, 33% experience physical IPV, and 26% experience sexual IPV. Self-esteem of women who experience IPV is associated with the cycle of IPV and influences their responses to IPV.

**Purpose:** The purpose of this concept analysis was to clarify the concept of self-esteem of women in the context of IPV. This foundational work is a critical step for developing a conceptual framework to guide future research studies.

**Method:** Walker and Avant’s concept analysis method was used. A literature search was conducted using the keywords of self-esteem, violence against women, IPV, and domestic violence. The literature search was limited to online dictionaries, and research articles published between 2013 and 2019, written in English, peer-reviewed, and availability of full text. Online dictionaries examined were the Oxford Dictionary of English, Merriam-Webster dictionary, Wikipedia, and Dictionary.com. Databases searched to conduct the literature review included EBSCOhost, CINAHL, PubMed, Scopus, EMBASE, SocINDEX, and Google Scholar.

**Results:** The key attributes of self-esteem are self-concept, self- affirmation, and self-respect. Identified key antecedents are power imbalances, gender inequality, and low social support mechanisms. The key consequences of self-esteem are detrimental physical and mental health issues.

**Discussion:** Limited definitions of self-esteem of women in the context of IPV were found. In nursing literature, self-esteem is discussed as low or high in various contexts including family violence and IPV. Further research is needed to develop clear conceptual and operational definitions that will be useful for several disciplines including nursing. Self-esteem is an important concept for developing future conceptual frameworks to improve the quality of life for women who have experienced IPV. Moreover, defining attributes must be clearly identified to develop valid, credible, and reliable instruments for measuring women’s self-esteem within the context of IPV.

**Keywords**

concept analysis, self-esteem, violence against women, intimate partner violence or domestic violence or partner abuse

## The Influences of Psychosocial and Cultural Factors on Women’s Responses to Intimate Partner Violence

### Ayse Guler^1^ and Carolyn R. Smith^1^

^1^University of Cincinnati, Cincinnati, Ohio, USA

**Background:** Intimate partner violence (IPV) is defined by the U.S. Centers for Disease Control and Prevention as “physical, sexual, or psychological harm by a current or former partner or spouse” (n.d.). Researchers reported that 15% to 71% of 24,097 women from 10 countries have experienced physical or sexual violence. Exposure to IPV is associated with detrimental health consequences among women survivors such as sexually transmitted diseases, unintended pregnancies, miscarriages, abortions, and physical and mental health diseases. To reduce consequences of IPV exposure, especially among racially and ethnically diverse populations, it is important to understand the role that psychosocial and cultural factors play in women’s responses to IPV experiences.

**Purpose:** The purpose of this integrative review was to identify the known psychosocial and cultural factors that influence women’s responses to IPV from existing literature.

**Method:** The Whittemore and Knafl framework guided the conduct of this integrative review. The PRISMA flow diagram was used to demonstrate the study selection process. Evidence levels and quality of included studies were assessed using the John Hopkins Nursing Quality of Evidence Appraisal tool and Lincoln and Guba’s Evaluative Criteria. Main results of studies were assessed using thematic analysis approach.

**Results:** Identified key themes comprised four concepts: socioeconomic factors, sociocultural factors, psychological factors, and help-seeking factors.

**Discussion:** Most studies reviewed identified contextual factors, including community and individual perceptions of violence against women, gender role expectations, cultural norms, and patriarchal structures, as the most prominent factors that influence women’s responses to IPV. Few studies produced clear evidence about psychosocial factors. Additional research is needed to understand the psychosocial factors that affect women’s responses to IPV.

**Implication to Practice:** Due to increasing ethnocultural diversity in the United States, more research needs to be conducted on psychosocial and cultural factors that influence women’s responses to IPV. In addition, research needs to explore racially and ethnically diverse groups of women who have experienced IPV living in the United States.

**Keywords**

social factors, psychological factors, cultural factors, intimate partner violence or domestic violence or partner abuse; psychosocial factors

## Effect of Antenatal Screening for Sickle Cell Disease on Intimate Partner Violence in the Sickle Cell Belt in Central India

### Nafisa Halim^1^, Patricia L. Hibberd^1,2^, Archana Patel^3,4^, and Anuradha Shrikhande^3^

^1^Boston University School of Public Health, Boston, Massachusetts, USA

^2^Boston University School of Medicine, Boston, Massachusetts, USA

^3^Indira Gandhi Government Medical College, Nagpur, India

^4^Lata Medical Research Foundation, Nagpur, India

Autosomal recessive sickle cell disease (SCD) is on the rise globally. Screening for SCD can be misunderstood and can lead to intimate partner violence (IPV), abandonment, and adverse outcomes but the frequency and risk of these outcomes is unknown. India is home to 27% of the world’s SCD births. SCD symptoms appear later in life in India; pregnant women may not know their SCD status. The “solubility test” which cannot distinguish between SCD and asymptomatic sickle cell trait (SCT) is used to screen for SCD in pregnancy. A positive test may be misunderstood as the pregnant woman having SCD, and not SCT. Pregnant women with a positive solubility test are counseled to return for hemoglobin electrophoresis to determine whether they have SCD or SCT. They are asked to bring their partner for the same test. Women with a positive solubility test and their partner undergo further testing to determine the SCD risk in the unborn child. We hypothesize that a positive solubility test increases the risk of adverse pregnancy outcomes, due to IPV. Informing a partner of a positive solubility test may result in IPV in India. Men may react with threats of or actual physical, emotional, or economic IPV (forced exile from the home, refusal to buy food for the family), blaming women of deception (intentionally hiding her status at the time of marriage), or for poor health and quality of life of the unborn child, associating the family with a stigmatized disease. IPV further compounds adverse pregnancy outcomes in both those with SCD and SCT. We are conducting a study in Central India to address this hypothesis in which we will report rates of IPV in 91 pregnant women with negative and 91 with positive solubility tests (a) just before the women are informed of the solubility test result and (b) during pregnancy. We will also report pregnancy outcomes. Our data collection ends in December 2019, and data analysis in March 2020. Results will be ready for dissemination by June 2020, and will inform programs improving pregnancy and fetal outcomes in women with SCD/SCT in India and elsewhere.

## In the Claws of Death: Fundamental Aspects of Intimate Partner Violence From the Perspective of Female Survivors

### Sigridur Halldorsdottir^1^ and Sigrun Sigurdardottir^1^

^1^University of Akureyri, Akureyri, Iceland

The purpose of this phenomenological research was to study fundamental aspects of intimate partner violence (IPV) from the perspective of female survivors. In-depth interviews were conducted with nine survivors. The interviews were audiotaped, transcribed, and thematically analyzed. The results show that the violence started in “the beginning phase” of the women’ relationships. The men were very often angry and displeased, wanted to know and control all the women’s actions, and expected obedience. The women never knew what to expect. In “the silencing phase,” the men used verbal abuse and intense humiliation and little by little the women felt they never did or said anything right and they were silenced. The women hoped that violent attacks would not be repeated. The men cut the women’s human contacts and took everything they could from them. The women began to experience a great sense of helplessness and hopelessness and began to feel almost invisible and being not even there. Looking back, all the women felt that their very lives had been threatened. They felt lifeless in “the claws of death.” The worst was the feeling of intense sense of guilt that robbed the women of basic human dignity. All the women used metaphors when trying to describe how they felt in “the living death phase.” In “the awakening phase,” something or someone came to the women’s aid and helped them escape from the claws of death. The women’s hearts began to “melt” and they began to feel again. “The way back to health and healing phase” was long and arduous for all the women. The authors conclude that IPV is extremely dangerous, and all efforts must be made to empower female survivors’ help-seeking and to help women in the healing process after being in such a life-threatening situation.

## Intimate Partner Violence as a Human Rights Violation

### Sigridur Halldorsdottir^1^ and Sigrun Sigurdardottir^1^

^1^University of Akureyri, Akureyri, Iceland

**Background:** Intimate partner violence (IPV) negatively affects the lives and health of millions of women worldwide and it is one of the greatest public health problems in the world today. The problem is still increasing and has been likened to an epidemic.

**Methodology:** The purpose of the present study was to analyze and compare the results of two qualitative studies of women’s experience of IPV to human rights from a legal, political, and ethical perspective. The qualitative studies used involved descriptions of 21 Icelandic women of their personal experience of partner violence. Human rights as described in six articles in the UN Declaration of Human Rights were used in the comparison as well as human rights as seen from a political and ethical standpoint.

**Results:** The comparison shows that IPV is a violence of basic human rights; it violates women’s right to personal freedom; their right to self-determination; their right to freedom of speech; their right to live life in communion with others on equal grounds; their right to live their life with a sense of security; and it violates their right to live life in dignity and self-respect.

**Conclusion:** One of the conclusions of the authors is that the reason why violence by intimate partners is as disempowering for women, as it is, is that it represents a violation against their dignity as persons, their freedom, and their independence. The analysis supports the view that using women’s descriptions of IPV and its effects can be a useful way to describe such violence as a human rights violation. The lawyer Evan Stark, and others, have recommended this way of studying IPV as a human rights violation. However, few seem to have done it.

## A Multidisciplinary System-Level Approach to Intimate Partner Violence Screening Among Obstetric Patients: A Quality Improvement Initiative

### Casey Harrison^1^, Annmarie Zimmermann^2^, and Kathleen Shurpin^1^

^1^Stony Brook University, Stony Brook, New York, USA

^2^Universal Primary Care, Olean, New York, USA

**Problem Statement:** Intimate partner violence (IPV) is a serious public health concern with many consequences, high costs, and increased risk of poor outcomes for pregnant women and their fetuses. Literature on how to screen and referral processes is inconclusive, and there is a wide variation in screening and response to IPV in health care.

**Purpose:** The aim of this multidisciplinary quality improvement project is to review existing literature on valid/reliable tools used in screening for IPV, to create and implement a standardized process for IPV recognition and referral with screening rates for pregnant women increasing from 0% to 60% of women being screened at initiation of obstetric care, and successful interventions following disclosure.

**Process:** Utilizing the Plan-Do-Study-Act (PDSA) framework, a pilot population of new obstetric patients are utilized to test and critique the new screening process, to improve both detection of, and response to, IPV at the facility. The Hurt Insult Threaten Scream (HITS) tool is selected as the method for IPV screening. Data are obtained on number of women being screened and results of screen.

**Outcome:** From October 2017 to March 2018, percent of patients screened using HITS increased which resulted in greater identification of IPV. Overall screening increased, with an average of 76% of the target population being screened. Positive screened patients started at a baseline of 0 and increased to 12% of the pilot population being identified as having a positive screen.

**Discussion:** Implementation of IPV screening for the pregnant population with a multidisciplinary, collaborative, system-level approach led to improved screening and referral rates. This approach provided real-time evaluation of the screening program for adaption as the program is implemented on a larger scale within the facility.

## A Combined Behavioral Economics and Cognitive-Behavioral Therapy Intervention to Reduce Alcohol Use and Intimate Partner Violence Among Couples in Bengaluru, India: Results of a Pilot Study

### Miriam Hartmann^1^, Saugato Datta^2^, Erica N. Browne^1^, Prarthana Appiah^3^, Rachel Banay^2^, Vivien Caetano^2^, Rosii Floreak^2^, Hannah Spring^2^, Anurada Sreevasthsa^3^, Susan Thomas^3^, Sumithra Selvam^3^, and Krishnamachari Srinivasan^3^

^1^RTI International, San Francisco, California, USA

^2^ideas42, New York City, New York, USA

^3^St. John’s Research Institute, Bengaluru, India

Alcohol use and intimate partner violence (IPV) are interconnected issues, with evidence showing hazardous drinking is an important contributing factor to IPV occurrence and severity in both developed and developing country settings. Despite this, only a limited number of alcohol reduction interventions have been tested in low- and middle-income countries (LMIC) for their efficacy in reducing IPV. This pilot intervention study tested a 1-month combined behavioral economics and cognitive-behavioral therapy intervention to reduce hazardous alcohol use and IPV in Bengaluru, India. Sixty couples were randomized to one of three study arms to test the effect of incentives-only and incentives plus counseling interventions compared with a control condition. Male participants in all arms took regular breathalyzer tests over the course of the intervention with those in the incentive arms earning a reward for sobriety (breath alcohol content [BrAC] < 0.01 g/dl). Couples in the incentives plus counseling arm participated in four weekly counseling sessions. Violence experienced by female participants was measured using the Indian Family Violence and Control Scale. Results showed that alcohol use decreased in both intervention arms. The counseling arm had a greater proportion of negative BrAC samples compared with the control arm (0.96 vs. 0.76, *p* = .03). Violence also decreased in both intervention arms, with the reduction persisting; the estimated mean violence score for the counseling arm was 10.8 points lower than the control arm at 4-month follow-up visit (*p* = .02). This study contributes important evidence to the field of alcohol reduction and IPV prevention approaches in LMIC settings and adds to the evidence suggesting that alcohol reduction is a modifiable means of addressing IPV. Given implementation feasibility and acceptability, as well as a dearth of other high-impact IPV interventions, this study shows value in continuing to explore the mechanisms at play in violence reduction, and testing efficacy in other settings.

## Transforming the Health System to Address Domestic and Family Violence: How Do We Know We Are There?

### Kelsey Hegarty^1^, Jane Koziol-McLain^2^, Elizabeth McLindon^1^, and Jo Spangaro^3^

^1^The University of Melbourne, Melbourne, Victoria, Australia

^2^Centre for Interdisciplinary Trauma Research, Auckland, New Zealand

^3^University of Wollongong, Wollongong, New South Wales, Australia

**Problem:** Comprehensive programs to help hospitals to identify and respond to family violence have had limited evaluation globally. System audit tools have been promoted in New Zealand, United States, and more recently in Australia, but how the findings of these evaluations should be interpreted is less clear.

**Aims:** To discuss findings from system audits of hospital programs for family violence in New Zealand and Australia to promote discussion on how to improve such programs. Second, to inform the research and evaluation field on the best way to measure outcomes both quantitatively and qualitatively.

**Format:** The session will begin with an introduction of health system family violence response infrastructure audit tools (5 minutes). We will present our practice-based evidence in case studies documenting tool purpose, development, and implementation in three locations. First will be the New Zealand Delphi audit tool evaluating the national Violence Intervention Programme (10 minutes). Second will be the development and implementation of a System Audit Tool across 18 hospitals in Victoria, Australia (10 minutes). Finally, results of an in-depth Australian case study from two states are presented to highlight factors assisting implementation of hospital programs (10 minutes). Following the case studies, we will introduce an emergent model for implementation in the complex setting of hospitals aimed to assist embedding family violence programs. This will be followed by small group discussions (45 minutes) critically analyzing the underlying assumptions for measuring and monitoring the health care system family violence response infrastructure. We will finish with summary and reflections on the way forward (10 minutes).

**Details of Presentations:** First will be the New Zealand Delphi audit tool evaluating the national Violence Intervention Program. The tool was revised in 2017 and implemented across the 20 District Health Boards in 2018 and 2019. Strengths and limitations of the revised tool will be discussed alongside clinical practice data and the model for improvement PSDA cycles. Results will be problematized: Are we seeing system change that signals improvements, are we moving in the right direction for transformation? Are changes sustainable? Second, Australian System Audit tools development and pilot implementation across Victoria will highlight factors then enhance or impede implementation. Results of an in-depth Australian case study from two states—New South Wales where screening in antenatal care has been deployed state-wide for 15 years and Victoria which has recently introduced screening—are presented to highlight factors assisting implementation of hospital programs.

**Issues:** Discussion will include how participants think the system audit tools actually capture the different elements of hospital programs, how implementation of programs can be improved, and how variation across settings influences the findings. We will also discuss the issues/challenges of capturing different types of violence, that is, child abuse, elder abuse, and how we evaluate response to patients who use violence. Finally, we will reflect on what are our underlying assumptions in this work and what have we learned from our practice-based evidence of system change in health settings.

## Testing a Brief Online Engagement Tool to Promote Men’s Help-Seeking for Domestic Violence and Abuse

### Kelsey Hegarty^1^, Mohajer Hameed^1^, Laura Tarzia^1^, Simone Tassone^2^, Matt Addison^2^, and Adam Hasandedic^3^

^1^The University of Melbourne, Melbourne, Victoria, Australia

^2^No to Violence, Australia

^3^Nexus Primary Health, Melbourne, Victoria, Australia

**The Problem:** Domestic violence and abuse is a hidden public health epidemic in communities, yet men who use violence are not really identified or responded to in the health settings. In Australia, current paradigms in responding to men’s use of violence in intimate relationships have focused on justice responses, not early engagement online to encourage and motivate help-seeking.

**Purpose:** This presentation aims to present the findings of a trial of a brief online healthy relationship tool (called BETTER MAN) to motivate men to seek help early for domestic violence and abuse.

**Study Design:** This trial utilized a single group repeated-measures design with both quantitative and qualitative outcome measures to test the feasibility and efficacy of BETTER MAN. Participatory design principles and co-design approaches were utilized to develop BETTER MAN.

**Sample:** Using an online recruitment strategy, in a 3-week period, 162 men enrolled with a 78% retention rate. They were culturally diverse men (average age of 33 years, 33% born outside Australia, 19% identified as in same-sex relationship, and 2.2% as Aboriginal and/or Torres Strait Islander).

**Data Collection:** Surveys before, immediately after use of the website, and at 3-months follow-up were administered online. Process evaluation was conducted via semi-structured interviews with some male participants.

**Analysis:** Paired *t* tests and general linear model of repeated measures were used to assess change over time for continuous data and non-parametric tests (e.g., Wilcoxon matched pair) for categorical data. Qualitative data were analyzed using thematic synthesis method.

**Results:** Positive outcome findings included an increase in mean intention to contact the men’s confidential counseling service (before 3.7, after 5.1, 3 months 5.3) and increase in mean confidence in the ability to seek help (before 4.9, after 6.1, 3 months 7.2) *p* < .001. Triangulating these results with the qualitative comments, participants mentioned that BETTER MAN “made me think about just how dangerous I can be, to my kids and partner it’s more than just a heavy fight”; “made me take a 360-degree view of myself and my actions.”

**Implications:** Overall, BETTER MAN is appropriate for English-speaking men, meets user requirements, and is highly acceptable and feasible, yet further research is required to test effectiveness with robust research methodologies such as a randomized controlled trial.

## Baseline Findings From Safe Pregnancy—Promoting Safety Behaviors in Antenatal Care Among Norwegian, Pakistani, and Somali Pregnant Women: A Randomized Controlled Trial

### Lena Henriksen^1^, Eva Marie Flaaten^1^, Jeanette Angelshaug^1^, Milada Cvancarova Småstuen^1^, Lisa Garnweidner-Holme^1^, Josef Noll^2^, Berit Schei^3^, Angela Taft^4^, and Mirjam Lukasse^1^

^1^Oslo Metropolitan University, Oslo, Norway

^2^University of Oslo, Oslo, Norway

^3^The Norwegian University of Science and Technology, Trondheim, Norway

^4^La Trobe University, Melbourne, Victoria, Australia

**Background:** Intimate partner violence (IPV) around the time of pregnancy is a recognized global health problem with several negative consequences. Despite this, little is known about the effect of violence assessment and intervention during pregnancy. We hypothesize that routine enquiry about IPV during pregnancy, in combination with information about safety behaviors, has the potential of increasing help-seeking behavior and interrupting IPV.

**Method:** Safe Pregnancy is a randomized controlled trial (RCT) to test the effectiveness of a tablet-based intervention that promotes safety behaviors. Midwives include women who attend regular antenatal check-ups. The intervention consists of a screening tool for violence and information about violence and safety behaviors. Eligible women answer baseline questions on the tablet including Abuse Assessment Scale (AAS). Women who screen positive on the AAS are randomized to an intervention video that contains information about violence and safety behaviors and the control group to a video with general information about a safe pregnancy. All women get information about referral resources. Follow-up is at 3 months post-partum. Main outcome measures are adoption of safety behaviors and quality of life. Baseline data include previous and ongoing experiences of IPV, sociodemographic characteristics, and questions regarding health and pregnancy. Descriptive analysis will be performed to assess the prevalence of IPV. Pearson’s chi-square test will be used to study frequencies and percentages and assess differences of characteristics of women reporting IPV and not. Multiple logistic regression will be performed to investigate the relationship between IPV and associated factors.

**Results:** Eighteen public health stations participated. Baseline recruitment was completed in August 2019. In total, 1,945 women have answered the baseline questionnaire. We will be able to present prevalence and associated factors of IPV among women attending routine antenatal care at the conference.

**Conclusion:** The study will provide important knowledge about the prevalence of IPV and associated factors among pregnant women in Norway, including two minority populations.

## Training Health Care Providers to Respond to Intimate Partner Violence Against Women: A Cochrane Systematic Review

### Leesa Hooker^1^, Naira Kalra^2^, Sonia Reisenhofer^1^, Gian Luca Di Tanna^3^, and Claudia Garcia-Moreno^4^

^1^La Trobe University, Melbourne, Victoria, Australia

^2^Johns Hopkins Bloomberg School of Public Health, Baltimore, Maryland, USA

^3^The George Institute for Global Health, Newtown, New South Wales, Australia

^4^World Health Organization, Geneva, Switzerland

**Problem Statement:** Intimate partner violence (IPV) against women results in substantial health problems. Training health care providers (HCPs) on addressing IPV may improve health outcomes and the care of survivors. There is a need to assess the impact of training HCPs and to identify the characteristics of successful training interventions.

**Purpose:** To assess the effectiveness of IPV training programs that seek to improve HCP identification and response to abused women.

**Study Design:** A Cochrane Systematic Review was undertaken during 2018–2019. Ten databases, relevant gray literature, and the reference lists of all included studies/systematic reviews were checked.

**Selection Criteria and Data Collection:** All randomized/quasi-randomized controlled trials comparing IPV training for HCP with no training, waitlist, training as usual, or placebo. We included studies with no training, waitlist, and so on with an additional component, but only if the additional component was implemented in both arms of the study.

**Results:** We included 17 trials involving 1,402 participants (mostly medical and nursing staff/students), predominantly from high-income countries. Studies varied across design, training content, pedagogy, and time to follow-up (immediately post training to 15 months post). As determined by the risk of bias assessment, study quality overall was rated low to very low, with studies often reporting on perceived, rather than actual practices. Meta-analyses compared seven trials of IPV training versus no training. IPV training improves provider attitudes toward survivors of IPV (SMD 0.676, 95% confidence interval [CI] [0.333, 1.018]), and their self-perceived readiness to respond (SMD 1.585, 95% CI [1.053, 2.117]). IPV knowledge also improves (SMD 1.095, 95% CI [0.431, 1.759]) yet evidence quality is low. Based on one study, training may improve HCP response (validating, safety planning) to survivors of IPV (odds ratio [OR] = 4.44, 95% CI [1.04, 19.00]), although the CI is wide and evidence quality very low. No effects were seen from IPV training on HCP referrals, or adverse outcomes reported.

**Implications:** In future, higher-quality trials are required with greater clarity and objective measurement of outcomes. Longer follow-up times would allow assessment of outcome sustainability.

## Talking to Fathers Who Have Abused: Enhancing the Child Focus in Social Child Welfare Investigations

### Ole Hultmann^1^, Ulf Axberg¹, Anders Broberg^2^, Maria Eriksson^3^, and Clara Iversen^4^

^1^VID Specialized University, Stavanger, Norway

^2^University of Gothenburg, Gothenburg, Sweden

^3^Ersta Sköndal Bräcke University College, Stockholm, Sweden

^4^Uppsala University, Uppsala, Sweden

Existing structured interviews and risk assessments with perpetrators of partner violence focus on the violence itself and the risk of repeated violence. These approaches lack relevant aspects of parenting, such as feelings of remorse, how the parent understands the child’s and the adult victim’s reactions to the violence, and whether perpetrators can identify problems that affect their relationship with the child. This study is conducted within the context of the Swedish iRiSk project (Insatser och risk-/skyddsbedömningar för våldsutsatta barn), which aims to develop structured risk and safety interviews in child welfare investigations. This paper presents a study that focuses on fathers as perpetrators. It examines (a) the feasibility of a structured interview, as assessed by professionals; (b) to what extent violent fathers are able to reflect on their violent behavior and its effect on parenting during the structured interview; and (c) to what extent violent fathers can provide relevant information to a child welfare investigation. The goal of the project is to improve the quality of risk and safety assessments and the protection of vulnerable children by enhancing the child focus in child welfare investigations about intimate partner violence and child abuse. The structured interview will be tested by professionals within child welfare investigations and with fathers who are in contact with a crisis center. Participants in the project are recruited through the Swedish iRiSk project funded by the Swedish National Board of Health and Welfare. Recorded interviews with the fathers will be analyzed according to scoring principles of the Parent Development Interview. The structured interview and the outline of the project will be presented as well as preliminary feedback from participating units and analyses of interview transcript.

## Symptom Cluster Pattern Recognition in Formerly Abused Women: A Pilot Feasibility Study

### Dori Steinberg^1^, Jiepin Cao^1^, and Qing Yang^1^

^1^Duke University School of Nursing, Durham, North Carolina, USA

**Problem Statement:** Women who have experienced intimate partner violence (IPV) suffer an array of symptoms that persist long after the abuse has ended. While symptoms have been shown to co-occur and even exacerbate one another, the nature of these relationships is poorly understood. Pattern recognition is a branch of machine learning focused on finding the patterns and regularities in data. One advantage of pattern recognition approaches to research is that significant quantities of data can be gathered from a relatively small number of individuals to discern previously indeterminant patterns. However, as an initial step, it is necessary to determine the feasibility of collecting such data.

**Purpose:** The purpose of this research is to address the following in a community-based sample of women who have previously experienced IPV:

**Aim 1:** To test the feasibility of exploring the patterns/clusters of symptoms of chronic pain, sleep disturbance, depression, and anxiety.

**Aim 2:** To test the feasibility of exploring to what extent diet, exercise, and sleep reduce symptoms and improve quality of life (QOL).

**Study Design:** The research uses an exploratory longitudinal design (4 months) with mixed methods to determine the feasibility of the study methods.

**Sample:** Thirty formerly abused, English-speaking women are being recruited.

**Data Collection Approach:** Participants are recruited via advertisements, primary care provider referral, and snowball sampling. After telephone screening, all study questionnaires are entered directly into REDCap and participants are instructed in the use of the Fitbit Charge 3. Participants receive emails/texts with reminders to submit bi-weekly data and meet monthly follow-up with the researchers.

**Analysis:** Descriptive and inferential statistics are being used to describe the sample. Although the sample size may not be sufficient for formal modeling, we will attempt to build latent growth curve models to identify different subgroups of participants who have distinct symptom trajectories over time.

**Results:** Data collection is underway and a full report of the findings is expected by Summer 2020.

**Implications:** Unsupervised approaches to pattern recognition require three steps: data acquisition, data analysis, and classification. This first step attempts to test the feasibility of innovative means of identifying patterns and trajectories in formerly abused women.

## Qualitative Re-Appraisal of Perspectives, Prevalence, and Management of Family Violence Among the Yoruba People—A Study of Cohorts From Ile-Ife, Nigeria

### Omolola O. Irinoye^1^ and Oluwasayo B. Ogunlade^1^

^1^Obafemi Awolowo University, Ile-Ife, Nigeria

**Problem:** The changing perspectives of violating behaviors globally are challenging cultural norms and contexts of tolerance of family violence. There is dearth of data to inform culturally appropriate interventions that will most reduce the occurrences of family violence in the study population. Data are needed to inform nature, contents, and methods of intervention for efficiency and effectiveness by nurses in family violence control.

**Study Design:** Exploratory design was adopted to collect qualitative data using focus group discussions. Fifty-five participants were purposively sampled among six categories of males and females across three generations of people in the age categories of 18 to 24 years, 25 to 45 years, and others above 45 years. Seven open-ended questions analyzed around the themes of conceptualization, perspectives, prevalent forms, persons mostly affected, perpetrators, management strategies, and the perceived effectiveness of such strategies among Yoruba people.

Results showed multiple words and descriptions of abuse and violence with no single word or conceptualization in the local Yoruba language to capture the concepts. Common forms of family violence identified were verbal assaults from parents to children, among couples, siblings; physical assault of children by parents, physical assault of parents by children; neglect of children, parents, spouses (mostly wives); sexual harassment; and sexual violence. Neglect of wives and children was perceived to be increasing, estimated to occur in one of four houses. Sexual harassment and sexual violence were perceived to be rare in family relationships in cultural context, but the latter was not considered an issue in marital context. A variant of sexual “touching” of young girls by young men was said to be tolerated among unmarried young men of a subethnic group. Age and gender were dominant factors in the use of common forms of violence. Informal approaches were common and more culturally acceptable in managing family violence but perceived inadequate.

The study concluded that family violence is a common phenomenon among the study population. The average nurse, irrespective of place of practice, needs to be appropriately trained to be sensitized to the burden of tolerated family violence in cultural context, to engage in risk assessment, family education and advocacy, and active intervention.

## Intimate Partner Violence Against Women and the Nordic Paradox: An Intersectional and Multilevel Analysis in the European Union

### Anna Karin Ivert^1,2^, Maria Wemrell^1,3^, Enrique Gracia^4^, Marisol Lila^4^, and Juan Merlo^1,5^

^1^Lund University, Malmö, Sweden

^2^Malmö University, Malmö, Sweden

^3^Lund University, Lund, Sweden

^4^University of Valencia, Valencia, Spain

^5^Center for Primary Health Care Research, Region Skåne, Malmö, Sweden

**Problem Statement:** The Nordic paradox refers to the high prevalence of intimate partner violence against women (IPVAW) in countries with highly rated levels of gender equality. Despite this paradox being one of the most puzzling issues in the field, this is a research question rarely asked, and one that remains unanswered.

**Purposes:** Our purpose is to explore the Nordic paradox by (a) analyzing whether this paradox reflects true differences in IPVAW prevalence, or whether the differences are the expression of confounding or information bias; (b) conducting multilevel analyses to understand the individual risk of IPVAW, as well as within and between country variations; and (c) conducting qualitative research in two countries exemplifying the Nordic paradox: Sweden and Spain.

**Study Design:** Data from the European Union Agency for Fundamental Rights survey on IPVAW for all European Union (EU) countries, and other surveys and international databases are used for quantitative analysis. In-depth interviews and focus group discussions, with different types of informants, are conducted in Sweden and Spain. In addition, review studies of previous research are made.

**Results:** Findings from the first studies indicate that the higher prevalence of physical and sexual IPVAW in Sweden than in Spain are not the result of measurement bias, and thus support the idea of the Nordic paradox. However, multilevel analysis shows that while significant differences in country averages exist with regard to IPVAW, country of residence does not discriminate very well with regard to individual experiences of IPVAW in cross-national comparisons. The relationship between experiences of IPVAW and country-level gender equality is weak and heterogeneous. Meanwhile, review studies of qualitative Nordic research on IPVAW, and of other research on IPVAW in Sweden, point not only to advancements made but also to remaining limitations and challenges in prevention and addressing of IPVAW in Sweden.

**Implications:** We expect that the scale of the project, the novelty of the research questions, and the methodological approaches used will allow for a better understanding of the Nordic paradox, and advance the existing knowledge on IPVAW to better inform future prevention initiatives both at the country and the EU levels.

## Nurse Home Visitors’ Efforts to Prevent, Recognize, and Respond to Child Maltreatment

### Susan Jack^1^, Andrea Gonzalez^1^, Lil Tonmyr^2^, Lenora Marcellus^3^, Colleen Varcoe^4^, Charlotte Waddell^5^, and Harriet MacMillan^1^

^1^McMaster University, Hamilton, Ontario, Canada

^2^Public Health Agency of Canada, Ottawa, Ontario, Canada

^3^University of Victoria, Victoria, British Columbia, Canada

^4^University of British Columbia, Vancouver, British Columbia, Canada

^5^Simon Fraser University, Vancouver, British Columbia, Canada

**Background:** Many families enrolled in nurse home visitation programs parent within social and economic conditions that may influence parenting capacity and put infants at risk for maltreatment, including exposure to intimate partner violence. The purpose of this study was to understand and describe nurses’ efforts and perceptions in preventing and addressing suspected child maltreatment within the context of nurse home visiting and legal reporting requirements.

**Method:** The principles of interpretive description guided decisions on purposeful sampling, qualitative data generation, and analysis. Forty-seven public health nurses in British Columbia, Canada, were interviewed. From these interviews, 49 unique case descriptions of how nurses attempted to prevent, recognize, and respond to suspected child maltreatment in their home visiting practices were analyzed using reflexive thematic analysis.

**Findings:** Initiating home visits early in pregnancy provided opportunities to develop a therapeutic alliance and implement a multi-faceted intervention plan to prevent child maltreatment. Following the infant’s birth, nurses described a process for managing mandated reporting within the context of a nurse–client relationship. This process included (a) “laying the groundwork” and providing anticipatory guidance, and then if potential indicators of maltreatment were recognized; (b) “walking the line” by considering whether to suspect child maltreatment; (c) “making sure” by consulting their supervisors as well as child protection social workers, and if maltreatment was suspected; (d) “making the call” to child protection services while (e) “striving to maintain the integrity of the nurse–client relationship”; and finally (f) “staying connected” as a source of support following child protection involvement.

**Implications:** Nurse home visitors play an important role in the primary prevention of child maltreatment. When indicators of potential child maltreatment were present, examination of experiential practice reveals that nurses are aware of the reporting legislation and used clinical judgment to consider whether maltreatment was suspected, and then if suspected, developed processes to report to child protection services in ways that maximized child safety, highlighted maternal strengths, and created opportunities to maintain the nurse–client relationship. Even with child protection involvement, nurses have a central role in continuing to work with families to support the development of safe and competent parenting.

## Critical Lessons Learned About Adapting, Implementing, and Evaluating Intimate Partner Violence Innovations for Home Visiting in International Practice Contexts

### Susan Jack^1^, Shauna Conway^2^, Tine Gammelgaard Aaserud^3^, Emma Larkin^2^, Sarah Tyndall^4^, Deirdre Webb^2^, and Ann Rowe^5^

^1^McMaster University, Hamilton, Ontario, Canada

^2^Public Health Agency, Belfast, Northern Ireland

^3^Nurse–Family Partnership, Regional Centre for Child and Adolescent Mental Health, Eastern and Southern Norway

^4^Family Nurse Partnership National Unit, London, United Kingdom

^5^Nurse–Family Partnership International Program, Denver, Colorado, USA

Considerable resources are invested in the development and evaluation of new practice innovations to support nurses and midwives identify and respond to intimate partner violence (IPV). In the United States, work has been invested to develop, pilot, evaluate, and scale an IPV innovation for the Nurse–Family Partnership (NFP) home visitation program. Internationally, six NFP programs (Australia, Canada, England, Northern Ireland, Norway, and Scotland) subsequently adopted and adapted the NFP IPV innovation. The objective of this symposium is to discuss critical lessons learned about adapting, implementing, and evaluating existing IPV innovations into new international contexts. The adoption of the NFP IPV intervention will be used as a case example. International leads from four countries will describe their local initiatives and then provide practical recommendations for researchers and decision makers committed to introducing and evaluating IPV innovations.

1. NFP IPV Innovation (5 minutes)

An overview of the five intervention components, including the clinical pathway and nursing curriculum.

2. Leveraging the Power of Collaboration and Committed Partnerships (15 minutes)

The benefits and challenges of multiple countries working simultaneously to adapt the same innovation yet tailor it to different contexts will be identified and discussed. The importance of central coordination, collaboration, and identification of similarities and differences in contexts will be considered.

3. Adapting existing interventions to reflect local needs and contexts (15 minutes)

The process of adaptation for a new context (Norway) will be explored, including identification of types of individual, team, organizational, community, and cultural adaptations required. Issues of language and translation, “fit” with local policy and service configurations as well as the balance between innovation and building on good nursing practice will be discussed.

4. Implementing new innovations in practice (15 minutes)

Changes to existing clinical practices require a committed local champion, leadership, support, and facilitated guidance. Examples from a checklist to support organizational readiness to adopt a new innovation will be summarized.

5. Practice-based evaluation (15 minutes)

It is good practice to evaluate adapted program innovations for local acceptability and feasibility within a new context. Findings of the Northern Ireland IPV innovation service evaluation will be shared. Reflections on the integration of the IPV innovation into a context of integrated health and social care services and the Northern Ireland policy context in relation to safeguarding and domestic violence risk assessment will be discussed. Considerations for existing nurse–client relationships will also be addressed.

6. How research methodology can support implementation (15 minutes)

A description of rapid cycle testing methodology and how this was used to support implementation of the innovation in England will be provided. Reflections on the benefits and drawbacks of short testing cycles and how these were used to combine quantitative data feedback with practitioner consultation will be discussed. These frequent conversations with teams around practice experience and reflection on practitioner-generated quantitative data created a dynamic which supported timely responses to challenges in the nurse experience of delivering the innovation and enabled rich learning to inform ongoing adaptation of this innovation into a new context.

Remaining time will be dedicated to discussion with attendees.

## The PATH to Knowledge Mobilization: Expanding Our Reach Using the ABELE Method

### Kimberley T. Jackson^1^, Tara Mantler^1^, and Sheila O’Keefe-McCarthy^2^

^1^Western University, London, Ontario, Canada

^2^Brock University, St. Catharines, Ontario, Canada

**Problem Statement:** Despite growing recognition of the intersection of pregnancy and intimate partner violence (IPV) that threaten women’s health and well-being, interventions designed to support those belonging to this social location remain largely underexplored. Promoting Attachment Through Healing (PATH) is a mixed-methods study evaluating the effect of cognitive-behavioral therapy (CBT) using a trauma- and violence-informed lens (TVICBT) among pregnant women with a history of IPV on infant–maternal attachment and maternal mental health. Case study findings suggest that TVICBT during pregnancy may enhance maternal coping and the development of positive maternal–infant attachment.

**Purpose:** Illuminating the influence of tailored interventions such as TVICBT on maternal and child outcomes may contribute to the enhancement of care for women. As such, novel and diverse approaches to mobilize key findings from this research may assist in raising awareness of the unique challenges faced by pregnant women with histories of violence, while stimulating attention of key stakeholders and decision makers. Our project aims to do just this. The PATH to Knowledge Mobilization presents science and art together in what promises to be a unique, transformative, and embodied glimpse into the lives of pregnant women who live(d) with IPV.

**Study Design, Data Collection, and Analysis:** We employed an arts-based creative research analysis—the ABELE method (Arts-Based Embodied Layered Exploration) to translate the journeys of three women through their pregnancy and postpartum care while receiving TVICBT. Women’s experiences informed four works of poetry reflecting the themes of (a) black deep corners, (b) triggering my thoughts, (c) breaking through the brokenness, and (d) perfectly imperfect. From here, volunteer artists recreated the poetry in works of art through a variety of mediums, to symbolically represent the women’s journeys.

**Results/Implications:** The works of poetry and art will be shared at an upcoming event, where artistic interpretations of the women’s experiences with TVICBT will be on display. It is hoped that this arts-informed research dissemination will provide an empathic and embodied introspection and reflection of the women’s experiences. We hope this has the power to engage attendees to help these stories be heard, visualized, felt, and contemplated—to raise awareness and understanding.

## EMBRACE: Engaging Mothers in a Breastfeeding Intervention to Promote Relational Attachment, Child Health, and Maternal Empowerment

### Kimberley T. Jackson^1^, Tara Mantler^1^, Brenna Velker^1^, and Shauna Burke^1^

^1^Western University, London, Ontario, Canada

**Problem Statement:** Breastfeeding promotion is a global public health priority. At-risk women, operationalized herein as having low income, limited social support, histories of intimate partner violence (IPV), and/or having adverse childhood experiences, often lack support and routine medical care from a primary care physician. For at-risk postpartum women, this may be particularly problematic when faced with breastfeeding challenges. A group of community-based Ontario family physicians have collaborated to create a clinic for at-risk postpartum women using trauma- and violence-informed care (TVIC). However, the effectiveness of this novel approach has not been evaluated.

**Purpose:** Efforts to provide community-based primary health care in the early postpartum period may be a powerful way to support at-risk mothers who choose to breastfeed their infants. As such, the purpose of this study is to evaluate the impact of a novel community-based family practitioner team providing TVI-postpartum care in the first days postpartum on breastfeeding outcomes, maternal bonding and infant attachment, and maternal mental health among at-risk women without a primary care provider.

**Study Design:** A mixed-methods sequential exploratory approach will be undertaken.

**Sample:** At-risk eligible women will be at least 18 years, speak/read English, be breastfeeding, and have access to the internet. Fifty women will receive the intervention, including TVI-postpartum care and support based upon breastfeeding goals. Fifty women will receive standard postpartum care from their obstetrician.

**Data Collection:** Data will be collected at baseline and 12 weeks postpartum via an online survey. Twelve women receiving the intervention will be invited to attend a 1-hour interview to explore their experiences with the clinic and its impact on breastfeeding. Quantitative data collected will include breastfeeding duration (in weeks) and exclusivity, breastfeeding self-efficacy, maternal feeding attitudes, infant attachment, and postpartum depression.

**Analysis:** Measures of central tendency and descriptive statistics will be computed. It is uncertain if this exploratory study will be powered to detect statistical differences between groups. A descriptive-interpretive approach will be used to inform/contextualize quantitative data.

**Results/Implications:** The study has just begun recruitment. We hope that the findings from this study will inform policy/decision makers, health care providers, and other key knowledge users.

## Validity and Reliability of Sullivan’s Quality of Life Scale Among Women With Histories of Intimate Partner Violence

### Diana A. Jaradat^1^, Marilyn Ford-Gilboe^2^, Carol Wong^2^, and Helene Berman^2^

^1^Jordan University of Science and Technology, Irbid, Jordan

^2^Western University, London, Ontario, Canada

**Background and Problem Statement:** Quality Of Life (QOL) measures have become a required part of life satisfaction evaluation among various populations. For populations with chronic stressors, measurement of QOL provides a meaningful way to determine the impact of life stressors such as previous abusive relationship on women’s lives in general. There is supporting evidence that women who have experienced intimate partner violence (IPV) have poorer overall QOL. There is a gap in researching QOL among women with histories of IPV due to the confusion in QOL concept and the lack of reliable and valid measures of QOL specific to this population. In this presentation, we describe the process of assessing the reliability and validity of Sullivan’s QOL scale among a community sample of women with histories of IPV.

**Purpose and Approach:** Advance the measurement of QOL by evaluating the psychometric properties of Sullivan’s QOL scale using a community sample of women with histories of IPV.

**Method:** Use data collected from women who had separated from an abusive partner and participated in the Women’s Health Effects Study. Exploratory and confirmatory factor analyses has been conducted to evaluate construct validity of QOL concept.

**Results:** The current paper provides evidence that the nine-item Sullivan’s QOL scale is both reliable and valid to measure QOL among women with histories of IPV. Our results supported a one-factor model that has been proposed by Sullivan. Based on our analysis results, all fit indices were acceptable. No items were identified for deletion based on the factor loadings, which were all greater than .4. Items 9 (how you spend your spare time) and 4 (fun and enjoyment) were correlated.

**Implications:** The psychometric evaluation of Sullivan’s QOL scale is an important step when determining to use it in future research. The results of this analysis provide further evidence for the use of Sullivan’s QOL scale among women with histories of IPV. In addition, the availability of the QOL Scale could enhance evaluations of the effects of programs and interventions that may help women with histories of IPV to improve their life quality.

## Organizational Implementation of Trauma- and Violence-Informed Care: A Multiple Case Study Analysis

### Tanaz Javan^1^ and Nadine Wathen^1^

^1^Western University, London, Ontario, Canada

Health and social inequities are increasing, especially for those already marginalized by systemic barriers such as poverty, and who face discrimination and racism. Many people across the socioeconomic spectrum have experienced various forms of trauma and violence; for those facing structural barriers and marginalization, these exposures, and their consequences, are often worse, making it even more difficult to access health and social services. There is a need to explicitly integrate equity-oriented care to overcome barriers and improve outcomes by addressing both individual and social/structural determinants of health. A core aspect of equity-oriented care is attention to the effects of trauma and violence and a commitment to minimizing harm by adopting what we call trauma- and violence-informed care (TVIC). This study is a multiple case study examining the implementation processes of TVIC in select organizations in London, Ontario.

## “You Need More Understanding”: Perinatal and Motherhood Experiences of Icelandic Mothers Who Are Survivors of Childhood Sexual Abuse

### Inga Vala Jónsdóttir^1,2,3^, Sigrún Sigurðardóttir^4^, Sigríður Halldórsdóttir^4^, and Sigríður Sía Jónsdóttir^4^

^1^Akureyri Hospital, Akureyri, Iceland

^2^Health Care Institution of North Iceland (Akureyri Health Clinic), Akureyri, Iceland

^3^Home Delivery and Home Service Midwife

^4^University of Akureyri, Akureyri, Iceland

**Background:** It is known that childhood sexual abuse (CSA) has long-lasting and profound consequences for womenʼs health but there remains a need for increased knowledge and deeper understanding of survivor’s experiences of the perinatal period and motherhood.

**Purpose:** To increase knowledge and deepen understanding of mothers, who are CSA survivors and how it can affect their perinatal and motherhood experiences.

**Method:** Qualitative analysis were conducted at the Vancouver School of Doing Phenomenology. Nine mothers, all CSA survivors, were interviewed once or twice, 16 interviews in total.

**Results:** All the mothers interviewed had experienced difficulties and trauma: sometimes during pregnancy and/or during birth, sometimes postpartum, or during their childrenʼs early life. Most of them reported many deviations from a normal perinatal period. The majority had experienced consequences of the abuse on their health and used health care services frequently. The overarching theme of the results is “You need more understanding.” This indicates a lack of awareness and knowledge among the public and health care professionals of the long-term impact of CSA on survivor mothers’ experiences of the perinatal period and motherhood. Three main subthemes appeared: (a) “There is always something wrong with me” relating to the effects of CSA on physical and psychological health, in general and during pregnancy. (b) “You’re most vulnerable during birth” relating to the effects on pregnancy, birth, feelings, and experiences of perinatal services, particularly midwifery services. (c) “Painful growth” influencing the maternal role, challenges, well-being, the need for support, and emotional processing.

**Conclusion:** Midwives, other health care professionals, and the public need more knowledge to deepen understanding of this phenomenon, to enhance midwifery and health care services, and mothersʼ experiences of the perinatal period and motherhood. This need could be met with a consulting or specialist midwife on the subject.

**Keywords**

childhood sexual abuse, survivor, perinatal period, motherhood, midwife, womenʼs health, phenomenology, interviews

## Explore the Ethical Challenges for a Research on the Effect of Group Therapy in Restoring the Psychological Well-Being of Women Survivors Raped During Genocide Against Tutsis

### Clémentine Kanazayire^1^, Jeanne Marie Ntete^1^, and Germaine Tuyisenge^2^

^1^University of Rwanda, Kigali, Rwanda

^2^Simon Fraser University, Vancouver, British Columbia, Canada

**Introduction:** Conducting research may be accompanied by a number of ethical challenges that researchers need to take into consideration at each stage of research. These challenges are often observed for the case of studies concerning different forms of abuse and other traumatic histories. In this context, little is known about the ethical issues related to conducting studies on victims of violence occurred during the genocide against Tutsis in Rwanda.

**Objective:** The aim of the present study is to explore the ethical challenges resulting from conducting and participating in a qualitative research that investigated the role of group therapy for women survivors raped during genocide against Tutsis.

**Methodology:** This article is based upon a study that included in-depth interviews conducted with women who had been raped during the genocide against Tutsis, 12 weeks after participating in the survey on the role of group therapy in restoring their psychological well-being, for exploring related ethical challenges.

**Results:** The majority of participants benefited financially and psychologically from participating in research. Some of the participants had reported feeling guilty, remorse, anxiety, and fear after completion of survey and didn’t need professional help. Others manifested symptoms of trauma and depression and consulted a counselor. One of the research assistants for the study manifested symptom of secondary trauma stress. This resulted in the researcher facing ethical distress, because participants had asked him to be silent on her condition and refused to go to seek help from health professional.

**Conclusion:** Based on the findings, it is crucial to include in research protocols an appropriate safe place where vulnerable participants receive support when needed after completing interview. In addition, researchers have to keep in mind to what extent the research can actually bring the intended change and, where applicable, they should avoid involving participants who have been previously part of studies on similar sensitive topics in case the involvement could cause harm to them.

**Keywords**

raped women, ethical challenge, genocide against Tutsis

## Developing Transnational Training for Nurses and Midwives to Support Survivors/Victims of Violence and Abuse at the Point of Access to Health Care Services

### June Keeling^1^, Wilf McSherry^2^, Vaiva Hendrixson^3^, Gloria Macassa^4^, Vasiliki Deligianni-Kouimtzi^5^, Jutta Lindert^6^, Freydis Freysteinsdóttir^7^, Marie Chollier^8^, and Ausra Motiejūnienė^9^

^1^Keele University, Staffordshire, United Kingdom

^2^Staffordshire University, Staffordshire, United Kingdom

^3^Vilnius University, Vilnius, Lithuania

^4^University of Gävle, Gävle, Sweden

^5^Aristotle University of Thessaloniki, Thessaloniki, Greece

^6^University of Applied Sciences Emden/Leer, Emden, Germany

^7^University of Iceland, Reykjavik, Iceland

^8^Centre Hospitalier Regional De Marseille Assistance Publique-Hoptaux, Marseille, France

^9^VUL Santara Clinics, Vilnius, Lithuania

Globally, almost a third of women experience some form of violence, coercion, or control that may significantly affect both their mental and physical health. Tackling and ending gender-based violence has been recognized by the European Commission, and is also supported by the European Union (2017) in protecting and supporting victims. The health service is one area that almost every women and girl will access at some time in their life, and this makes it an ideal forum through which to recognize victims of this violence, and provides an ideal opportunity to engage with them. To achieve more positive outcomes for survivors/victims, nurses and midwives need to be able to engage with them, recognize and assess risk, and offer constructive support. While many health care providers including nurses and midwives receive training, it is country specific, and may be irregular or incomplete. Violence against women is prevalent across all countries, and migration might result in a victim trying to access support in several countries. There is currently no standard transnational training focused on nurses and midwives. This presentation will explore the challenges, implications, strengths, and approaches of working with colleagues across Europe to develop and deliver the first transnational training program for nurses and midwives in the recognition and education in violence, abuse, and neglect. This innovative educational opportunity represents an original approach of joining forces across Europe to deliver this training.

## Adapting a mHealth Intervention for Teens in Unhealthy Relationships

### Rachel Kennedy^1^, Amber Clough^1^, Rachael Turner^1^, and Nancy Glass^1^

^1^Johns Hopkins University School of Nursing, Baltimore, Maryland, USA

Teen dating violence (TDV) is highly prevalent and poses a serious threat to adolescent health, well-being, and safety. Recently, the first report on U.S. estimates of teen homicides due to dating violence found that 7% of teens killed were killed by a current or former partner. Prevention and response efforts are challenging to develop and implement effectively as teens have different relationship norms than adults and are less likely to access services such as hotlines or domestic violence advocates. Technology is a critical tool to reach teens and expand their access to personalized relationship health and safety information. One innovative tech-based approach is the use of chatbots. Chatbots are computer programmed “conversational agents” that mimic a conversation with a real person and are an emerging technology to leverage in delivering health interventions. We will present on our team’s development of a chatbot for teens in unhealthy relationships. We will discuss the adaption of an effective intervention for adult survivors (the myPlan App) for teens ages 15 to 17 to assess their relationship health and safety and receive tailored safety information and resources. Collaboration with youth advisors, findings from qualitative interviews with teens, advocates, and providers on how best to adapt the intervention into the chatbot platform, and implications for future development of adolescent interventions using human-centered design principles will be discussed.

## Trajectories of Adolescent Cyber Dating Abuse Experiences

### Rachel Kennedy^1^, Yu Lu^2^, Jacquelyn Campbell^1^, and Jeff Temple^2^

^1^Johns Hopkins University School of Nursing, Baltimore, Maryland, USA

^2^University of Texas Medical Branch, Galveston, Texas, USA

Adolescent populations access the internet every day, allowing online spaces to shape not only their daily activity and communication but also their social and dating norms. Despite these significant trends in internet usage, available evidence around experiences of online or cyber dating abuse, harassment, or stalking has been remarkably limited and largely formative in nature. This exploration of trends in cyber dating abuse experiences will therefore help to inform these gaps in understanding and help to identify potential opportunities for intervention. The current study aims to answer the following three questions: (a) What are the trajectories of cyber dating abuse experiences among adolescents over time? (b) Do these trajectories differ by gender? (c) Are experiences of cyber dating abuse associated with dating history, amount of time spent online, or in-person dating violence experiences? This secondary data analysis is taken from the dataset of the Dating It Safe longitudinal study among seven public high schools in southeast Texas. A total of 1,042 high school students participated in the study over five time-points at 12-month intervals between 2013 and 2017, and the current analysis looks at three of these years. Data collection occurred in person during school hours or online, depending on participant school enrollment status. For the first study question in this analysis, growth mixture modeling was performed to characterize the development of cyber dating abuse over time. Multinomial regression and conditional models were then used to assess how covariates predict class membership. Findings reveal statistically significant and non-significant associations with cyber dating abuse experiences for young people, specifically highlighting the important role of partner gender, online behavior, and in-person violence use and experience in these patterns. Implications for future development of public health interventions for adolescent experiences of cyber dating abuse will be discussed.

## The Influence of Peer-Based Polyvictimization on Disordered Eating Behavior: Findings From a Representative Survey of Adolescents

### Melissa Kimber^1^, Masako Tanaka^1^, Andrea Gonzalez^1^, Jennifer Couturier^1^, and Harriet L. MacMillan^1^

^1^McMaster University, Hamilton, Ontario, Canada

**Problem Statement:** Emerging literature details the cumulative impacts of exposure to multiple forms of violence, that is, “polyvictimization,” on adolescent well-being. However, there is a dearth of studies examining the impacts of exposure to multiple forms of peer-based violence on disordered eating behavior (DEB) among youth.

**Purpose:** This study examines (a) the association between teen dating violence, peer-based bullying, and DEB among a representative sample of youth living in Ontario, Canada, and (b) the extent to which, if at all, sex modifies these associations.

**Study Design:** We conducted a secondary analysis of data from the 2014 Ontario Child Health Study, a province-wide representative survey of families with children in Ontario, Canada. Youth aged 14 to 17 years (*n* = 2,396) completed a computer-assisted, self-administered questionnaire to assess experiences of peer-based bullying, teen dating violence, DEB, and associated correlates, including child maltreatment.

**Data Analysis:** Pooled estimates via linear regression and bootstrapped standard errors was used to address our research objectives. Missing data were addressed using multivariate multiple imputation by chained equations (MICE).

**Results:** Adolescents who reported exposure to peer-based bullying reported significantly greater DEB compared with their non-bullied peers, even after adjusting for a history of exposure to teen dating violence and child maltreatment. A significant sex by teen dating violence interaction effect was found; sex-stratified models indicated that teen dating violence showed a significant association with DEB among females only.

**Implications:** Regardless of sex, adolescents exposed to peer-based bullying reported significantly greater DEB. The current study revealed increased risk for DEB among females exposed to teen dating violence as compared with male adolescents. These findings lend support for the need to develop and evaluate targeted preventive interventions specifically tailored to female and male adolescents with independent and intersecting exposures to different forms of peer-based victimization. The broad range of associations for IPV exposure during the first 10 years, and for current exposure in particular, demonstrates the urgency to improve identification and response to family violence for children.

## Recognize and Respond to Family Violence: Implementation and Evaluation of VEGA

### Melissa Kimber^1^, Donna Stewart^2,3^, Meredith Vanstone^1^, Delphine Collin-Vezina^4^, Gina Dimitropoulos^5^, Harriet L. MacMillan^1^

^1^McMaster University, Hamilton, Ontario, Canada

^2^University Health Network, Toronto, Ontario, Canada

^3^University of Toronto, Toronto, Ontario, Canada

^4^McGill University, Montreal, Quebec, Canada

^5^University of Calgary, Calgary, Alberta, Canada

**Problem Statement:** A low-cost, scalable, and efficacious educational intervention for improving practitioner knowledge, attitudes, skills, and behavior (KASB) related to recognizing and responding to family violence has not previously been identified. The newly developed Violence, Evidence, Guidance, Action (VEGA) intervention is the only transdisciplinary educational intervention that has been developed to improve trainee and practitioner KASB related to safely recognizing and responding to family violence in Canada.

**Purpose:** VEGA is an online educational intervention that includes didactic instruction, “how to” video clips, gaming technology, as well as formative feedback to enhance practitioner KASB. Informal feedback from learners who have completed the VEGA modules suggests that these individuals perceive improvements related to KASB competencies itemized in the VEGA Competency Framework. However, a formal implementation and evaluation effort is needed. This presentation will provide an overview of the VEGA educational intervention, present some of the VEGA learning tools, as well as detail the proposed protocol for its formal implementation and evaluation among trainees and licensed professionals belonging to eight national professional organizations in Canada.

**Study Design:** We will use a multiphase mixed-method research design that incorporates the collection of quantitative and qualitative data via three conceptually and methodologically linked research projects. All phases and components of this project will be informed by a novel application of the Active Implementation Framework (AIF). The AIF is an evidence-based synthesis of frameworks derived through a systematic review of implementation successes and failures in the areas of education, nursing, mental health, social services (e.g., child welfare), as well as others.

**Implications:** The Recognize and Respond Project will initiate a rigorous implementation and evaluation program that will provide important information about the value and impact of VEGA in improving trainee and practitioner recognition and response to family violence. Secondary implications are the generation of implementation strategies that support uptake and sustainability of VEGA among health care and social service providers in Canada. The extent to which VEGA leads to improvements in the well-being of individual and families who have experienced and who are at risk of experiencing family violence will be evaluated in a future project.

## Journey to Trauma Integration: Re-Trusting, Re-Building, and Re-Embracing Selfhood, Life, and the World

### Sachiko Kita^1^

^1^The University of Tokyo, Tokyo, Japan

For women victims of gender-based violence (GBV), accepting and integrating trauma into their selfhood, life, and world is a long and challenging journey. We conducted a Clinical Ethnographic Narrative Interview (CENI) with 23 Japanese survivors of domestic violence to identify the phases of trauma integration and factors promoting it. We then identified a subset of 11 women who described that they had achieved integration and used grounded theory approach to discover the processes that characterized their integration journey. The results revealed six phases: “confusion,” “overwhelmed,” “awareness,” “fighting,” “overlooking,” and “integration.” In addition to these phases, other critical recovery tasks were revealed, including “rebuilding the boundary between myself and others” and “trusting others and seeking their help again.” These recovery tasks helped protect their feelings and autonomy, and enhanced their understanding and acceptance of trauma and its impacts. Implications for practice include understanding the complexity of the trauma integration processes, the skills necessary to achieve it, and possible cultural differences.

## Prevalence and Covariates of Traumatic Brain Injury–Related Violence Among a Sample of College Women Experiencing Relationship Violence

### Kathryn Laughon^1^, Nancy Perrin^2^, Amber Clough^2^, Jamie Barnes-Hoyt^2^, Racheal Turner^2^, Karen Grace^2^, Sherry Kausch^2^, and Nancy Glass^2^

^1^University of Virginia, Charlottesville, Virginia, USA

^2^Johns Hopkins University School of Nursing, Baltimore, Maryland, USA

Traumatic brain injury (TBI) is a concern for women experiencing intimate partner violence (IPV). Young women ages 18 to 24 are at high risk for IPV and many young women in this age range are enrolled in higher education. Therefore, the purpose of this study was to describe individual and relationship factors associated with TBI-related IPV among young women enrolled in colleges in two U.S. states.

**Method:** This study examines baseline data from the myPlan study. Students enrolled in college in Oregon or Maryland, 18 to 24 years old, had access to safe email and a safe device with internet access, identified as a woman, and self-reported experience of dating/partner violence in the past 6 months were eligible to participate (*N* = 355). TBI was measured by women’s report of violence associated with TBI in the last 6 months (drowning, smothering/suffocating, “choking,” and/or blows to the head.)

**Findings:** Nearly 28% (*n* = 99) of college women reported at least one form of TBI-related violence in the last 6 months, with attempted strangulation most common (23.7%, *n* = 84). Women reporting TBI-related violence were slightly older (21.38 vs. 20.73 years, *p* = .020), had been in the abusive relationship for longer (28.36 vs. 22.58 months, *p* = .023), and are more likely to be African American (27.3% vs. 16.3%, *p* = .020) than their abused peers who had not experienced TBI-related IPV. Women with TBI-related IPV reported higher scores on the Composite Abuse Scale (41.89 vs. 23.39, *p* < .001). Women with TBI-related IPV also reported more reproductive coercion (37.4% vs. 19.2%, *p* < .001), had higher depression scores (37.41 vs. 29.03, *p* < .001), and were substantially more likely to have missed class because of the abusive relationship (68.8% vs. 42.5%, *p* < .001) than their abused peers who did not report TBI-related IPV.

**Discussion:** Findings from this study align with findings from other studies of strangulation and TBI-related IPV. Women who experience TBI-related IPV experience more serious overall violence, including reproductive coercion. TBI-related IPV in college women also associated with potential for academic difficulties secondary to depression and missing more classes. These findings should be used to inform campus-based physical, mental, and reproductive health, academic, and advocacy education and services.

## Enhancing the Foundational Validity of Forensic Findings in Strangulation Examinations

### Kathryn Laughon^1^, Andrea Cimino^2^, and Sherry Kausch^1^

^1^University of Virginia, Charlottesville, Virginia, USA

^2^Johns Hopkins University School of Nursing, Baltimore, Maryland, USA

**Problem and Significance:** Nonlethal strangulation of women poses a significant threat to public safety but is difficult to prosecute because it leaves little external physical evidence when not assessed using careful forensic examination protocols. Many strangulation injuries are non-visible and easily overlooked, such as loss of memory, involuntary urination, and breathing changes. Other injuries, such as petechiae and bruising throughout the face and neck, can be non-specific and may be attributable to other causes, including underlying disease, medication, and other assault-related injuries. The current state of strangulation science confines expert testimony to merely describing injuries attributed to strangulation “based on the expert’s experience and training,” thereby leaving testimony open to critique by the defense. Expert testimony that is able to quantify to the likelihood that observed injuries were attributable solely to strangulation (vs. other assault-related injuries or disease processes) may drastically improve conviction rates, ultimately reducing homicide risk and increasing public safety.

**Purpose of the Session:** This session will describe participant characteristics of a recently funded study that examines forensic data on assaults against women with and without reported strangulation with the purpose of quantifying the likelihood that observed injuries were attributable solely to strangulation. We currently have begun analysis of a database of approximately 20,124 patients, aged 13 and greater, of which 6,388 reported strangulation and 13,736 reported assaults other than strangulation.

**Approach/Innovation:** Our description of the proposed study includes (a) the development of a repository of forensic strangulation data, (b) our approach to use probabilistic modeling—including exploring use of machine learning—to quantify the certainty/uncertainty that a constellation of injury patterns is suggestive of strangulation by making data-based comparisons of assaults against women with and without reported strangulation, and (c) the intention to develop and distribute written guidelines on these findings to clinicians and prosecutors and defense attorneys.

**Lessons and Implications:** More rigorous research differentiating strangulation injuries from other injuries with probabilistic metrics is needed to hold offenders accountable while ensuring that incorrect interpretation of injury findings will not lead to wrongful convictions.

## Trauma Inquiry Using Trauma-Informed Approaches

### Annie Lewis-O’Connor^1^

^1^Brigham and Women’s Hospital, Boston, Massachusetts, USA

Within the context of longitudinal medical care for adults, health care providers have a unique opportunity to inquire and respond to the traumatic life experiences affecting the health of their patients, as well as a responsibility to minimize retraumatizing these patients during medical encounters. While there is literature on screening women for intimate partner violence, and there is emerging data on pediatric screening for adverse life experiences, there is sparse literature on inquiry of broader trauma histories in adult medical settings. This lack of research on trauma inquiry results in an absence of guidelines for best practices, in turn making it challenging for policy makers, health care providers, and researchers to mitigate the adverse health outcomes caused by traumatic experiences and to provide equitable care to populations that experience a disproportionate burden of trauma. This presentation will describe best practices for trauma inquiry within an anchoring framework of trauma-informed care principles, which includes tiered screening starting with broad trauma inquiry, proceeding to risk and safety assessment, and emphasizes the importance of focusing on strength and resilience. Best practices for trauma inquiry which include tiered screening starting with broad trauma inquiry, proceeding to risk and safety assessment as indicated, and ending with connection to interventions. The focus is not on disclosure rather on what happened to you and how did that experience affect your heath.

## Low Commitment to Partners and Precursors of Intimate Partner Violence Among Pregnant Women in a Home Visitation Program

### Qing Li^1^, Jacquelyn Campbell^2^, and Vincent J. Palusci^3^

^1^San Diego State University, San Diego, California, USA

^2^Johns Hopkins University School of Nursing, Baltimore, Maryland, USA

^3^New York University, New York City, New York, USA

**Objective:** We aimed to examine low commitment to partners and intimate partner violence (IPV) among pregnant women to optimize home visitation programs. A published randomized controlled trial (RCT) included preventive, relationship-focused interventions for IPV and reported moderation effects among mothers without IPV baseline experience. We moved upstream and early to measure weak bonds as precursors of IPV. According to Travis Hirschi’s social bond/social control theory, social bonds are “internalization of accepted norms, awareness, and sensitivity to the needs of others which promote conformity in society.” Each dimension of the bonds—attachment, commitment, involvement, and belief—ties partners to societal rules, thus theoretically informally controlling and preventing violence. This study investigates the role of relationship commitment and IPV during pregnancy.

**Study Design:** We performed secondary analyses of baseline data from an RCT of an IPV preventive intervention embedded within the Nurse–Family Partnership (NFP) program in the Multnomah County, Oregon. First-time pregnant mothers were randomized to either intervention or control (NFP only) condition, and 238 were consented and interviewed at baseline, and 1- and 2-year follow-up. The intervention included structured screening for IPV, the brochure-driven intervention, and adaptation of the Within My Reach Curriculum. Physical, sexual, and psychological IPV in the past 12 months were measured with the Conflict Tactics Scale (Revised). A higher level of commitment was operationalized as (a) being married or engaged, (b) planned pregnancy together, and (c) dedication to the partner. Their associations with IPV and 95% confidence intervals were assessed in logistic regressions.

**Results:** Three commitment indicators were associated with a lower prevalence of physical IPV victimization: (a) 0.51 [0.26, 1.00], (b) 0.44 [0.22, 0.86], and (c) 0.27 [0.15, 0.50], and perpetration: (a) 0.50 [0.26, 0.95], (b) 0.34 [0.17, 0.66], and (c) 0.33 [0.18, 0.58]. Only planned pregnancy together was associated with a lower prevalence of psychological IPV victimization (0.37 [0.21, 0.65]) and perpetration (0.38 [0.21, 0.67]).

**Implications:** Higher commitment to the partner was associated with a lower prevalence of physical IPV at baseline. Further steps include to assess their changes over times and explore the other dimension as well as relationship commitment as a mediator or moderator to prevent IPV in home visitation programs.

## Dating Violence Among College Students in China: A Cross-Sectional Study

### Fuqin Liu^1^, Haiying Han^2^, and Judith McFarlane^1^

^1^Texas Woman’s University, Houston, Texas, USA

^2^Zhejiang University City College, Hangzhou, China

**Problem Statement:** Violence in dating relationships is a widespread problem on college campuses. Previous studies suggest that attitude toward relationship violence in Chinese societies has been shaped by a history of Confucian patriarchy.

**Purpose:** The purpose of this study is to investigate beliefs about dating violence among college students in modern China.

**Study Design:** This was a cross-sectional study design.

**Sample:** A sample of 2,262 college students in a large university in Southeast China, recruited in June 2018, responded to questionnaires containing questions about dating violence.

**Data Collection Approach:** Data collection was performed in June 2018. Freshman, sophomore, junior, and senior college students were informed about the study. Students who volunteered to participate were asked to fill out the study questionnaires during evening self-study time. Informed consent was obtained before data collection. Students self-administered the demographic questionnaire, the Attitude Justifying Violence Questionnaire, and the Dating Violence Scale. The total amount of time needed to fill out the questionnaires was about 20 to 30 minutes. A total of 2,588 students participated. Of the returned questionnaire, 326 were not complete, resulting in the final sample of 2,262. The total sample was comprised of 1,022 male and 1,240 female students.

**Analysis:** This study employed the SPSS to analyze data, and conducted correlation and multiple regression analyses to examine the relationships among attitudes justifying violence, and dating violence experience.

**Results:** The results showed that 988 (43.7%) participants had experienced a dating relationship, and 75% had experienced dating violence. A total of 951 (42.1%) students agreed one’s sex has something to do with dating violence, while 1,267 (56.1%) thought age is a factor. A total of 923 (40.1%) students thought it has an impact on dating violence. The demographic data of the sample show 1,416 (62.7%) students were without siblings. Based on analysis, the female victimization was more common and male victimization is acceptable.

**Implications:** Our findings indicate that dating violence among Chinese college students is common. Factors contributing to the high incidence of dating violence warrants further investigation. Culturally tailored violence prevention programs are needed for college students.

## Subjected to Sexual Abuse During Childhood—How Is the Oral Health Experienced?

### Sarah Månsson^1^, Louise Larsson^1^, and Eva Wolf^1^

^1^Malmö University, Malmö, Sweden

**Problem Statement:** Sexual abuse leads to serious, short- and long-term personal consequences for mental and physical health, including oral health and dental fear. Compared with patients without known dental fear, those suffering are reported to have more missing teeth, more dental caries, and more inflammatory oral conditions. The victims of abuse might therefore be considered deprived of the potential for a satisfying oral health. How victims of sexual abuse experience oral health is, however, limited.

**Purpose:** To study how individuals who have been subjected to sexual abuse as a child experience their oral health.

**Method:** Eleven participants (10 women), 19 to 56 years of age, who experienced sexual abuse as a child were purposively selected and in-depth interviewed. The participants were encouraged to tell in their own words and in as much detail as possible about the perception of their oral health. To provide for empirical variation, some strategic selection criteria were applied: sex, age, and class. The interviews were recorded digitally and transcribed verbatim. The collected material was analyzed according to Qualitative Content Analysis.

**Results:** All participants reported dental fear to some degree, which had prevented them from visiting dental care regularly. Oral health was associated with strong emotions, including shame, guilt, and anxiety but also occasionally pride. The preliminary results indicate difficulties among the participants to perform daily oral hygiene procedures and, in general, they wished for an improved oral health.

**Implications:** Knowledge of how patients who have been subjected to sexual abuse perceive their oral health might be a contribution to an improved patient-centered dental treatment and care.

## Sharing Personal Experiences of Accessibility and Knowledge of Violence in a Rural Context

### Tara Mantler^1*^, Kimberley T. Jackson^1^, Edmund J. Walsh^1^, and Selma Tobah^1^

^1^Western University, London, Ontario, Canada

Women who experience intimate partner violence (IPV) in a rural context face unique barriers and inhibiting social structures compared with their urban counterparts. The purpose of this study was to explore the intersection of women’s experiences of rural health care and domestic violence services within the context of IPV in Southwestern Ontario, Canada. An interpretive case study was used consisting of in-depth qualitative interviews with eight participants who had used both health services and a rural shelter in the past 6 months. The analysis was positioned within a critical feminist intersectional lens, which allowed for exploration of larger social structures and their potentially oppressive influence on women’s experiences, as well as the complexity of lived experiences as women hold multiple roles in society. The following three themes were uncovered: (a) Strengths (What I Have)—which highlights the power of positive interactions with frontline workers in shelters and health care settings; (b) Challenges Related to Structural Violence Through Policy (What I Need)—which underscores the systemic barriers in accessing social services such as housing and receiving appropriate health care within the context of shelter curfews and policies in walk-in clinics (i.e., one issue per visit); and (c) Systems and Stigma (What Does Not Exist)—which brings to light the discomfort of neighbors close to the shelter and lack of understanding of the long-term effects of violence by health care providers. The implications of this study support the need for improved education, system-level integration, and the need to examine how policies across sectors are interacting in intended and unintended ways.

## Hopes and Experiences of Women Survivors of Intimate Partner Violence When Seeing Psychologists

### Sally Marsden^1^, Kelsey Hegarty^1,2^, and Cathy Humphreys^1^

^1^The University of Melbourne, Melbourne, Victoria, Australia

^2^The Royal Women’s Hospital, Melbourne, Victoria, Australia

**Problem Statement:** Intimate partner violence (IPV) often has long-lasting mental health effects on survivors, yet we know little about women survivors’ hopes and experiences when seeing psychologists after IPV. Women’s voices about this topic are largely absent in the literature which is predominantly focused on clinical studies to support evidence-based interventions. The few previous studies have predominantly heard from women who have seen specialist counselors connected to domestic violence services; however, most psychologists do not have this specialist experience.

**Purposes and/or Questions/Hypotheses:** The purpose of this study was to explore women survivors’ hopes and experiences of seeing psychologists after IPV and thus inform practitioner education and response.

**Study Design:** The study was a qualitative study.

**Sample:** We interviewed 20 women survivors who had seen psychologists after IPV.

**Data Collection Approach:** Semi-structured interviews.

**Analysis:** We thematically analyzed the transcribed data from the interviews.

**Results:** Themes related to women’s experiences were (a) negative experiences where psychologists mirrored abuse, (b) the effects that these negative experiences had on the survivors, and (c) positive experiences which counteracted the abuse. Themes developed from women’s hopes were (a) to have their expertise recognized and respected (b) that the psychologist will look beyond the symptoms and see the person and (c) not to have agendas imposed on them by the psychologist.

**Implications:** This study has implications for improving the education and response of psychologists. It is important that psychologists are trained to employ violence- and trauma-informed practice to prevent mirroring abuse. This includes respecting survivors’ expertise and working collaboratively rather than imposing default interventions based only on survivors’ symptoms.

## Understanding Survivor Reactions and Behaviors in the Aftermath of Sexual Assault: Evaluation of an Online Curriculum

### Robin Mason^1^, Janice Du Mont^1^, Stephanie Lanthier^1^, and Sheila Macdonald^1,2^

^1^Women’s College Hospital, Toronto, Ontario, Canada

^2^Ontario Network of Sexual Assault/Domestic Violence Treatment Centres, Toronto, Ontario, Canada

Sometimes the behaviors and reactions of sexual assault survivors can challenge the understanding of family, friends, and professionals. As a supportive response to disclosure has been shown to be integral to the healing of survivors, the failure to understand the full range of potential behaviors in the aftermath of sexual assault can result in non-supportive responses to the survivor’s disclosure, including questions, doubt, or disbelief expressed through verbal or nonverbal behavior. It is particularly critical that those working in the helping professions be familiar with and understand reactions to sexual assault that may appear to be counter-intuitive. Failure to do so can seriously undermine the survivor’s confidence and negatively affect any future help-seeking, while also perpetuating a problematic social discourse about how “real” survivors of sexual assault behave. We developed an evidence-informed, competency-based, online curriculum to educate health and social services providers to the basis for these commonly misunderstood reactions. In so doing, we hope to also challenge social discourses about the so-called acceptable reactions to sexual assault, thereby improving the experience of disclosure for survivors. With financial support from the province of Ontario, the curriculum was made available in 2019 without charge to health and social service providers across the province. Drawing upon lists of women’s studies programs, campus sexual assault centers, lists of participants in our other online courses, and with the support of some of the health professions colleges, we emailed information about the curriculum to approximately 2,000 individuals or organizations. By August 2019, approximately 800 individuals had registered for the online curriculum. In this session, we present participant demographics to understand where there has been the greatest uptake of the curriculum and results of the pre- and post-tests designed to evaluate changes in understanding about the common but frequently misunderstood reactions to sexual assault.

## “Is There Something I Could Have Done?” Design and Implementation of Physician Education to Support Colleagues Experiencing Domestic Violence

### R. Mason^1^, M. Kanee^2^, J. Sloggett^3^, and S. Laredo^1^

^1^Women’s College Hospital, Toronto, Ontario, Canada

^2^Independent Advisor and Trainer on Human Rights and Health Equity, Toronto, Ontario, Canada

^3^University Health Network, Toronto, Ontario, Canada

It is well-known that those who work in hospital systems face challenges in educating physician colleagues to the realities of domestic violence in our patient populations. Time constraints, “fear of opening Pandora’s Box,” feelings of inadequacy, anxiety about having to report to child welfare authorities, and just plain fear have all worked against educators’ and advocates’ best efforts to help physicians do a better job of recognizing and responding to patients who may be experiencing intimate partner violence. The untimely and tragic murder of a physician by her physician spouse, an event that significantly affected their families, patients, and colleagues, also inspired the first gathering of a group of medical chiefs of staff from three hospitals to discuss ways of responding to the news. Their first concern was providing emotional support to their colleagues, but they also wanted to undertake some significant action. They considered an education campaign but quickly realized they didn’t know enough about the issue themselves and so convened a larger group including those with some expertise in issues of intimate partner violence and abuse as well as individuals from the hospitals’ legal, communications, and human resources departments. The result? A short online course about domestic violence that was specifically developed for physician colleagues. Through action and advocacy, course completion is now required across the 12 University of Toronto–affiliated teaching hospitals. In this session, we describe (a) specific considerations in developing a course on domestic violence relevant to physicians and (b) the processes that resulted in the teaching hospitals requiring course completion as a condition of physicians maintaining their hospital privileges.

## A Scoping Review That Demonstrates the Health Inequity of Cervical Cancer From Intimate Partner Violence

### Amanda St Ivany^1^, Mercedes McCoy^2^, Julia Mead^1^, and Ivy Wilkinson-Ryan^1^

^1^Dartmouth-Hitchcock Medical Center, Lebanon, New Hampshire, USA

^2^University of New Hampshire, Durham, New Hampshire, USA

Intimate partner violence (IPV) is defined as behaviors intended to exert power and control over another individual, including physical, sexual, verbal, emotional, and financial abuse and or/stalking. Women with a history of IPV may suffer chronic health problems related to their physical and psychological well-being: chronic pain, insomnia, hypertension, substance use disorders, acute physical injuries (e.g., concussions, broken bones), gastrointestinal disease, and increased risk of sexually transmitted diseases and infections. Scoping review aims were to synthesize IPV screening methods and barriers for gynecological settings, explore associations between cancer type and IPV, and highlight gaps in the evidence to guide trauma- and violence-informed models of care (TVIC) to promote health equity.**Method:** Scholarly articles were retrieved from PubMed, Web of Science, and Google Scholar. Keywords included domestic violence, dating violence, spousal abuse, marital rape, sexual assault, sexual violence, IPV, cancer, oncology, metastases, colposcopy, Papanicolaou test, and neoplasms. Articles were included if they were available in English and published between the years of 2009 and 2019. In total, 423 articles were retrieved across all databases and 30 articles were included for final review.

**Results:** Our main findings are related to (a) screening methods—need for comprehensive staff training on IPV screening protocols and integration of IPV screening tools into medical records; (b) barriers to cancer screening and follow-up—IPV impedes women’s ability to comply with screening and follow-up, providers express discomfort with IPV screening, women with IPV history experience fear of re-traumatization during pelvic exams; (c) health inequities—increased incidence of cervical cancer exists among survivors of IPV.

**Conclusion:** Women who have experienced IPV are less likely to be up-to-date with preventive cancer screenings and are also more likely to be diagnosed and treated for cervical cancer when compared with women who have not reported experiencing IPV. TVIC guidelines for pelvic exams need to be addressed in future research. All staff in gynecological settings need to be trained with protocols for IPV screening to appropriately address the correlation of cervical cancer associated with IPV. IPV screening must be addressed from a TVIC perspective to disrupt health inequities by preventing health disparities.

## The FAST-PTSD APP for Predicting Clinical PTSD 7 Years Following First Contact for Abuse Services: A New Tool for Rapid Triage

### Judith McFarlane^1^

^1^Texas Woman’s University, Houston, Texas, USA

**Problem:** Post-Traumatic Stress Disorder (PTSD) is among the most common mental health sequelae of intimate partner violence (IPV) for women. PTSD influences both the day-to-day functioning and long-term health of women and the emotional and behavioral adjustment of their children. The mental health of women who experience IPV often improves over time, but many women still suffer long after they first seek help for IPV. Unfortunately, long-term prospective studies on the mental health of women who received support services for IPV are lacking, as are tools for predicting the likelihood of future PTSD.

**Purpose:** To evaluate the predictive validity of the First Assessment Screening Tool (FAST-PTSD) for clinically significant symptoms of Post-Traumatic Stress Disorder 7 years after women first sought help following IPV.

**Study Design:** Longitudinal study of 300 women who sought help for IPV for the first time.

**Method:** Women who sought intimate partner violence support services for the first time in 2011–2012 (*N* = 300) completed the FAST-PTSD to determine risk for chronic PTSD. Seven years later, in 2018, 271 (90%) women completed a seven-item screen for presence or absence of clinically significant PTSD symptoms. A two-step binary logistic regression was conducted to determine the 7-year predictive validity of the First Assessment Screening Tool for clinically significant symptoms of Post-Traumatic Stress while controlling for baseline PTSD symptoms.

**Results:** Over 25% of the women reported clinically significant PTSD at 7 years. Baseline moderate- and high-risk scores on the FAST-PTSD predicted clinically significant levels of PTSD. Moderate risk was associated with nearly two and one-half times (odds ratio [OR] = 2.4) the risk of clinically significant symptoms of PTSD, and high risk with nearly eight times (OR = 7.8) the risk of PTSD at 7 years.

**Conclusion:** PTSD is commonly associated with IPV and if untreated can compromise functioning of women and their children. The FAST-PTSD is a valid predictor of significant clinical PTSD symptoms 7 years following first contact with IPV support services. Using the FAST-PTSD to triage women at risk for sustained PTSD to early, preventive intervention may improve outcomes for women and their children.

## Expressive Art Therapies to Facilitate Attunement Between Mother and Child

### Dorcas McLaughlin^1^

^1^Webster University, St. Louis, Missouri, USA

Teen mothers are a vulnerable group of parents for reasons other than their youth. They experience high levels of psychological distress due to the social disadvantage and adverse childhood experiences (ACEs) which precede many of these pregnancies. ACEs include traumas associated with family violence, neglect, mental illness, alcoholism, substance abuse, or criminal activity. Teen mothers may also face additional traumas, first as children and later as adults, from residing in dangerous neighborhoods with high rates of crime and unemployment. The high prevalence of psychological distress is particularly troubling in light of the evidence that trauma-related symptoms compromise maternal functioning, mothers’ physical and mental health, family relationships, and children’s development. The purpose of this presentation is to describe Mothers Growing Together (MGT), a resilience model, strength-based group intervention, tailored to low-income teen mothers. MGT integrates expressive arts therapies to improve emotional regulation, facilitate attunement, build self-esteem, cultivate positive peer relationships, and develop resilience and coping skills among teen mothers. Quantitative and qualitative data from a pilot study that evaluated the effectiveness of MGT will be discussed. Attunement with one’s self and one’s child can be strengthened in teen mothers using expressive art therapies in the delivery of services. These approaches are consistent with advances in the neurobiology of chronic stress and are therefore effective in bridging the mind–body disconnections associated with exposure to trauma and early adversities. They provide valuable tools for modifying psychological distress and improving responsive parenting. Expressive art therapies are teen friendly, easily adapted for home or group settings, and support the teen’s capacities and aspirations to be good parents. They are also easily modified for parents of varied ages and background.

Learning Objectives

- Describe the neurobiology of chronic stress and its implications for the long-term physical and mental health of teen mothers and their children
- Discuss the role of attunement in the management of stress and the maternal–child relationship
- Explain the relationship between self-soothing and the development of empathy
- Use specified expressive art therapy approaches to deliver parent teaching experiences related to strengthening maternal attunement

## Survivor Health Professionals: Is Personal History of Gender-Based Violence Associated With Clinical Care of Survivor Patients and What About the Role of the Health Care Workplace?

### Elizabeth McLindon^1^, Cathy Humphreys^1^, and Kelsey Hegarty^1,2^

^1^The University of Melbourne, Melbourne, Victoria, Australia

^2^The Royal Women’s Hospital, Melbourne, Victoria, Australia

**Problem Statement:** Health professionals are at the frontline of responding to the health and well-being impacts of gender-based violence (GBV). Research (Nursing Network on Violence Against Women International [NNVAWI] 2016) with Australian health professional women about the prevalence of GBV against them found GBV was common; 11.5% had experienced intimate partner violence in the last 12 months, while 45.2% had experienced GBV across their lifespan. Little is known about whether GBV affects health professionals’ clinical care of survivor patients and what role the health care workplace should have in better supporting survivor staff.

**Research Questions:** (a) Is personal experience of GBV associated with a health professional’s clinical care of GBV survivor patients? (b) What needs do survivor health professionals have of their hospital workplace? (c) What are the views of hospital managers about their role in responding to staff survivors?

**Study Design:** Descriptive, cross-sectional survey with health professionals at an Australian hospital followed by individual and group interviews with hospital managers.

**Sample:** In total, 471 female health professionals (45.0% response rate) and 18 hospital managers.

**Data Collection Approach:** Health professionals participated in an online or paper-based survey and hospital managers were interviewed.

**Analysis:** Using logistic and linear regression, we examined whether health professionals’ exposure to lifetime GBV was associated with clinical care on specific measures of training, attitudes, identification, and intervention. We thematically analyzed open-ended survey and interview data.

**Results:** Survivor health professionals reported greater preparedness to intervene with survivor patients in a way that is consistent with ideal clinical care. These survivors wanted understanding in their workplace about the effect of trauma. Survivors and managers believed that formal resources and support were essential, including trained managers and cultural change. Challenges to creating an environment where staff felt emotionally safe to disclose GBV and seek support were identified.

**Implications:** Personal GBV experience in the lives of health professional women is not a barrier, and may be a facilitator, to good clinical care of survivor patients. Using a trauma-informed organizational framework, hospitals could establish systems which simultaneously support both staff survivors and patients in their recovery from GBV.

## The Health, Well-Being, and Relationships Project: How Is Nurses Health, Well-Being, Work, and Community Service Use Linked to Their Experience of Gender-Based Violence?

### Elizabeth McLindon^1^, Kristin Diemer^1^, and Kelsey Hegarty^1,2^

^1^The University of Melbourne, Melbourne, Victoria, Australia

^2^The Royal Women’s Hospital, Melbourne, Victoria, Australia

**Problem Statement:** Previous research has identified that the prevalence of all forms of gender-based violence (GBV) may be higher for nurses, midwives, and carers (hereafter referred to as “nurses”) than the general Australian population. However, little is known about how GBV affects nurses’ health and well-being, and employment in health care, and whether existing health, community, and specialist services meet their needs.

**Research Questions:**

1. What is the prevalence of GBV against Australian nurses including the perpetration of such violence?

2. What are the physical, emotional, and social health associations of such violence?

3. How have nurses experienced existing health, community, and GBV services for these issues?

4. What support needs do survivor nurses have of their union and their health care workplace?

**Study Design:** Online survey of all female and male members of a large nursing Union called the “Australian Nursing and Midwifery Federation (Vic Branch).”

**Sample:** We are currently collecting data from *N* = 79,000 Victorian nurses (90% female, 10% male). The expected response rate (based on current data collection and previous surveys) is 12% to 15%, giving an expected sample size of 9,500 to 1,200 participants.

**Data Collection Approach:** An online survey has been sent via email to eligible nurses. Data collection will cease in September 2019; analysis will be complete by May 2020.

**Analysis:** Given the expected sample size, we will have enough power (>80% power, 5% significance level) to detect differences in health and well-being of participants who have experienced GBV and those who have not. We will present descriptive and inferential statistics and tests of probability. Factor analysis and multiple linear regression will be employed to analyze the relationship between selected characteristics and dependent variables of interest and to identify latent variables that may group together.

**Results:** Our findings will report on associations between GBV (intimate partner violence, sexual assault, reproductive coercion, digital abuse, child abuse, and use of abusive behaviors), health, and well-being measures and health, community, and GBV service measures.

**Implications:** It is anticipated that this project could have far-reaching implications for GBV workplace support programs in any health setting that nurses work.

## Incidence of Cesarean Delivery Among Women With a History of Gender-Based Violence: What Do We Know?

### Julia Mead^1^, Amanda St. Ivany^1,2^, and Robyn Puleo^1^

^1^Dartmouth-Hitchcock Medical Center, Lebanon, New Hampshire, USA

^2^Geisel School of Medicine, Lebanon, New Hampshire, USA

**Problem Statement:** Gender-based violence (GBV; including rape, sexual violence, intimate partner violence, childhood sexual abuse, and/or sex trafficking) is estimated to affect one in three women in the United States, with similar prevalence worldwide. During pregnancy, a history of GBV may lead to decreased likelihood of presenting for prenatal care, poor maternal weight gain, as well as an association with certain birth outcomes, such as pre-term birth and low birthweight infants. Several studies in Norway and Iceland have explored the association between exposure to sexual violence and incidence of cesarean section (CS) or operative vaginal delivery (OVD). One study found a 13-fold increase in rates of CS among women with a history of sexual assault and a 10-fold increase in rates of OVD. Another study noted an increased rate of elective CS among women with a similar history, as well as increased rates of OVD. There is a dearth of knowledge regarding the impact of GBV on delivery method in the United States.

**Question:** Our research will explore the relationship between maternal history of GBV and obstetrical delivery outcome, such as cesarean section, in the United States’s obstetrical cohort.

**Hypothesis:** We hypothesize that experiencing GBV increases a woman’s likelihood of delivery via cesarean.

**Study Design:** Using a large centralized data set, the Pregnancy Risk Assessment Monitoring System (PRAMS), a retrospective data review will be performed using variables such as demographics, medical history, labor course, and birth outcome. Although history of GBV is widely under-reported, a large dataset will allow for suitable analysis. A similar retrospective data analysis will take place using patient data drawn from an obstetrical cohort delivering at a rural, tertiary care center in New Hampshire.

**Implications:** Impacts of this research will include increased knowledge regarding previously unexamined risk factors for cesarean delivery. In addition, this analysis will allow for improved ability for prenatal care providers such as midwives, nurses, and Ob-Gyns to adequately and appropriately engage in pre-labor counseling, as well as to provide improved prenatal, intrapartum, and post-partum care for women with a history of gender-based violence.

## Understanding How Pornography Can Drive Intimate Partner Violence, and What Can Be Done About It

### Darryl Mead^1^, Cindy Pierce^2^, and Mary Sharpe^1^

^1^The Reward Foundation, Edinburgh, Scotland

^2^Independent Author, Educator, and Speaker, Etna, New Hampshire, USA

**Description of the problem to be addressed:** In this session, we will explore the role pornography consumption plays in creating and sustaining intimate partner violence. Before the internet, relatively few people consumed enough pornography to create widespread harms visible at the population level. With the arrival of free, streaming, and increasingly violent, video pornography in the mid-2000s, pornography became popular entertainment for a large part of the population. More than half of adult men in many societies today are regular consumers. Porn’s impact is reflected in the World Health Organization’s adoption of Compulsive Sexual Behavior Disorder (CSBD) in June 2018. International studies suggest more than 80% of people diagnosed with CSBD have a problem with pornography use.

**Aims of the Symposium:** As educators who work with health and social care professionals outside the regular Nursing Network on Violence Against Women International (NNVAWI) context, we bring new perspectives that can contribute to the development of strategic policies and practices to reduce the growing power of pornography as a factor driving gender-based violence.

**Symposium Format:** As the literature has established clear links between pornography consumption and gender-based violence, our group will examine three broad aspects of this landscape to help identify good ways forward.

Darryl Mead will discuss how porn has become a serious problem for an increasing number of boys and men, and what can be done. Porn creates sexual expectations for consumers. Women are portrayed in a way that reinforces the false idea that they are always sexually available and enjoy rough sex. Porn consumers often imitate what they see online, which reduces inhibitions and boosts feelings of male entitlement in a way that ignores consent or intimate pair-bonding behavior. At the same time, porn encourages extreme acts, coercion, and acceptance of sexual scripts, giving men the dominant role. How can a boy, man, or father make the journey from porn enthusiast to successfully quitting porn? How can schools, men’s groups, and online recovery communities support this journey?

Mary Sharpe will lead on two areas of educational work. The first is schools: provide lesson plans for different age groups to discuss pornography’s impact and train-the-trainer sessions to help teachers address sensitive issues and the science behind these matters. The second area is support for health care professionals. The Reward Foundation runs workshops for Continuing Professional Education accredited by the Royal College of General Practitioners. A diverse range of professionals attend: psychotherapists; sex therapists, doctors, nurses, psychiatrists, pastors, social workers, lawyers, and so on, all of whom engage with pornography consumers. Our curriculum focuses on how the brain is affected by chronic overuse of hardcore pornography and the associated addiction-related brain changes. In a radical departure from previous teachings in sexology where porn was encouraged, we will look at research from the behavioral addiction field which indicates that for some users, pornography should be recognized as an emerging addiction with the potential for harmful sexual behavior. We also discuss social prescribing as a first line of treatment.

Cindy Pierce’s focus is on educating college and high school students, as well as parents, coaches, and educators about the impact of porn on their sexual and social choices. Students need informed adults to help them navigate the cultural messaging they consume through porn, media, and social media. She explores the realities of the porn industry, specifically how women performers are purposefully dehumanized and coerced to perform acts beyond their boundaries.

**Focus of the discussion:** If pornography use is a driver for gender-based violence, what can we do to improve policy, health care, and social responses in our communities?

## Addressing Gender-Based Violence With a Viral Video

### Darryl Mead^1^, Cindy Pierce^2^, and Mary Sharpe^1^

^1^The Reward Foundation, Edinburgh, Scotland

^2^Independent Author, Educator, and Speaker, Etna, New Hampshire, USA

We are a part of a worldwide collective of sex and relationship educators who explore innovative ways to influence public policy and public behavior around pornography consumption and gender-based violence. Since early 2019, we have collaborated to create elements of a new toolkit to engage all genders in ways that engage and motivate young people. As a proof of concept, we have created a short (2 minutes 13 second) animation, which covers many facets of problematic pornography consumption. Based on a real person, its central focus is on the way pornography induces erectile dysfunction in many boys and men as well as normalizes sexual violence against women. Two major factors influenced the video-making process: the introduction of age verification legislation by the UK government, which is being seen as a test case for the whole world, and the content had to be acceptable and credible to the international self-help movement for people with excessive or compulsive pornography consumption. The video is designed for social media use and is available under a CC BY-NC-ND 4.0 license to maximize its reach through free transmission. We hope this video will become viral and develop a life of its own. It aims to reach into communities to help prevent gender-based violence. The video ends with a list of free helpful resources from several independent sources. The video has already been integrated into different sets of lesson plans for use in schools and is used for training health care professionals. It is also featured on a number of pornography self-help sites. We have used a wide range of techniques to encourage viral transmission. We are now undertaking research to quantify its impact. The session would end with a showing of the video. Preview it at https://www.youtube.com/watch?v=Ehsh77hmgPA

## Prevalence and Risk Factors for Intimate Partner Violence in Arab Countries: A Systematic Review

### Amera Mojahed^1^, Nada Alaidarous^2^, Susan Garthus-Niegel^3^, and Janice Hegewald^1^

^1^Institute and Policlinic of Occupational and Social Medicine, Dresden, Germany

^2^Western University, London, Ontario, Canada

^3^Clinic and Policlinic of Psychotherapy and Psychosomatic, Dresden, Germany

**Problem:** Intimate partner violence (IPV) profoundly damages the physical, sexual, reproductive, and psychological health, as well as social well-being of female individuals and families. IPV is estimated to be very high in the Middle East and North Africa. However, data specific to the Arab countries have not yet been systematically analyzed.

**Purpose:** This systematic review presented evidence from studies on IPV prevalence among women living in the Arab states. It also examined the risk factors according to the integrative ecological theoretical framework for IPV and the potential cross-cultural aspects that could be related to it for women living in Arab countries.

**Method:** Using PRISMA guidelines, searches in four databases (Embase via Ovid, PubMed, PsycINFO via ProQuest, and SCOPUS) were conducted, supplemented by hand searching of reference lists from retrieved studies and previous reviews. Studies in English, French, and Arabic containing primary empirical research data involving women who have experienced IPV (aged ≥ 13 years) living in the 22 Arab countries were included. Studies that primarily investigated IPV only among pregnant women or refugee populations were excluded. We conducted a meta-analysis of prevalence data and a narrative synthesis of the risk factors data. The methodological quality of all eligible studies was assessed using a checklist developed using the Methodological Evaluation of Observational Research (MORE) and (MEVORECH) tools.

**Results:** The lifetime prevalence of overall IPV ranged from 18% to 58.6%, and the 12-month prevalence of overall IPV from 11.9% to 87%. Psychological violence was the most common type of IPV in both lifetime and 12-month prevalences, ranging from 15.7% to 73.4% and 30% to 91%, respectively. Risk factors at the individual level were either related to victims or perpetrators of IPV. Factors relating to marriage, conflict within the family, family’s living conditions, and so on were explored and included within the family level, whereas factors relating to the extended family and the nature of marriage were included in the community level. Finally, risk factors relating to the cultural context that are influenced by the political and religious backgrounds were included in the societal level of the integrative ecological model.

## Living With Family Violence: Teen’s Perspectives

### Nora Montalvo-Liendo^1^, Andrew Grogan-Kaylor^2^, Eliza D. Alvarado^3^, Angeles Nava^4^, and Catherine Pepper^5^

^1^Texas A&M University, McAllen, Texas, USA

^2^University of Michigan, Ann Arbor, Michigan, USA

^3^Region One Education Services, Edinburg, Texas, USA

^4^Texas Woman’s University, Houston, Texas, USA

^5^Texas A&M University, Round Rock, Texas, USA

**Problem Statement:** Intimate partner violence (IPV) is a common and tragic event in the lives of women and children worldwide. Immigrant Latina women are generally considered to be at the highest risk of all ethnic or racial groups, with 50% experiencing lifetime IPV. Millions of children live in homes where intimate violence occurs.

**Purpose:** The development of the Latinx Teen Club (LTC) was conducted by a multidisciplinary team from Nursing, Psychology, and Social Work. This study sought to understand the experiences of Latinx teens’ perspectives on their experience living in violent homes.

**Study Design:** The study used focus group settings. Two separate groups were conducted.

**Sample:** Twenty-five Latinx teens participated. The LTC, community-based and group-based intervention for teenagers from Latino families, was conducted in a border community.

**Data Collection Approach:** All women who visited a local domestic violence shelter were provided with information on the pilot program. Their teen sons/daughters were invited to participate. Two distinct pilot focus groups met for 10 weeks, for 1 hour a week. Each week, the teens were presented with a group dialogue topic (e.g., safety, fears, and worries, etc.) relevant to IPV.

**Analysis:** Two measures were used, the Strengths and Difficulties Questionnaire (SDQ) and the Attitudes and Beliefs About Violence (ABAV). The SDQ is a 25-item self-report measure of adolescents’ symptomatology symptoms.Strengths scores may be calculated by summing response to the five items on the Prosocial scale. The ABAV scale is a 10-item self-report measure of teens’ thoughts and feelings toward fighting in the family.

**Results:** The participants were 13 to 17 years with a mean age of 14.3 years. Teens identified the need for speaking out when violence is occurring in their homes. Teens perceived benefits from sharing their personal experiences. The outcome of this multidisciplinary innovative project was the development of two distinct brochures that included teen participants’ perspectives on experiences relevant to family violence.

**Implications:** The study findings suggest it is feasible to work with teens particularly those affected by family violence. Future research to understand the implementation and outcomes of a culturally appropriate program are warranted.

## Evaluating Nurse-Led Long-Term Support Groups for Women Survivors of Intimate Partner Violence

### Nora Montalvo-Liendo^1^, Robin Page^2^, Jenifer Chilton^3^, and Angeles Nava^4^

^1^Texas A&M, McAllen, Texas, USA

^2^Texas A&M, Bryan, Texas, USA

^3^The University of Texas at Tyler, Tyler, Texas, USA

^4^Texas Woman’s University, Houston, Texas, USA

**Problem Statement:** Support groups are essential to ensure the mental health and well-being of women survivors of intimate partner violence (IPV). Many support groups available to women survivors are short term (10–12 weeks) and facilitated by paid counselors or paid advocates.

**Purpose:** To explore the outcome of nurse-led long-term support group sessions for Latina women survivors of IPV.

**Study Design:** A qualitative descriptive approach was used to conduct face-to-face interviews.

**Sample:** Women survivors of IPV who had attended bi-weekly support group sessions between 2009 and 2018 at a non-profit agency that provides services to victims of domestic violence and sexual assault were eligible to participate in the study. The agency is located in South Texas adjacent to the United States–Mexico border, where 93% of the population is Hispanic.

**Data Collection Approach:** Interviews were conducted by research team members from October 2018 to December 2018. An open-ended approach was used implementing a semi-structured interview guide. The interviews were recorded with participants’ permission.

**Analysis:** The tapes were transcribed by a professional transcriber and a graduate research assistant. The Spanish interviews were translated to English and back to Spanish for accuracy by a professional transcriber. Each transcript went through a two-phase coding process, which included the initial line-by-line and focused coding.

**Results:** Forty-nine women agreed to participate in the study. The majority of the women (*n* = 47) self-reported income US$14,999 or less. Participants in the study had a total of 146 children. The women described the lack of courage, increased fear, and anxiety they had prior to attending the support sessions. Many of the women shared stories on how the groups helped them “find their identity” and how the groups “increased their self-esteem.” In addition, they talked about the positive impact the groups had on their children and how they felt safe discussing the challenges they were now facing post the abuse in a group setting that was available when they needed to “vent.”

**Implications:** Nurses are seen as service agents for many victims of violence. However, nurses can also be agents of hope for many survivors of violence by facilitating peer group sessions.

## Alert Signs of Dating Violence Among College Students: From the Perspective of Campus Services

### Derby Munoz-Rojas^1^ and Cristóbal Ching-Álvarez^1^

^1^University of Costa Rica, San Jose, Costa Rica

College students represent a vulnerable group for dating violence (DV), as many of them might have few experiences on dating relationships; therefore, they might have limited resolution conflict skills. Researchers have consistently found that both victimization and perpetration of DV have negative consequences on health and well-being, including anxiety, physical injury, and low academic performance. DV in college campuses is a prevalent and complex issue that requires a public health approach to be addressed. Thus, providers of most of the student-oriented services should be engaged in detecting and responding to DV. Although screening for DV in different services (e.g., counseling and health care centers) have been worldwide implemented for many universities, in Costa Rica, it is not a common practice, leading to an inadequate recognition of this problem and therefore failing to address it. Indeed, little is known about the knowledge and ability of professionals offering health and academic services to students to recognize signs of DV. Therefore, this descriptive qualitative study aims to address this gap by identifying the perception of these professionals about alert signs of DV among college students in Costa Rica. Thirty-five professionals from the five public universities in the country were recruited for this study, including dentists, nurses, counselors, physicians, psychologists, and social workers (age = 44 + 8.5 years old, experience = 6 + 6.3 years working at the university). From a conventional content analysis of the transcription of the interviews, two themes emerged that map the complex interaction among social, sexual, physical, academic, and psychological signs of DV. Results also stressed that warning signs patterns are different between victims and perpetrators, thus screening criteria should consider this difference. Social, psychological, and cybernetic alert signs are the most difficult to recognize, as victims tend to normalize them. Perpetrators are more likely to exhibit strong personalities, while victims are more likely to report low academic performance. These findings might inform to researchers about the constructs that should be included in DV screening instruments for the country. Results might also be used for training academic and health care professionals working on campus services, so they might develop the skills to identify students going through DV experiences.

## Adapting and Evaluating a Sexual Violence Prevention Program From the United States to Ghana: Camp-Life

### Abdul-Aziz Seidu^1^, Eugene K. M. Darteh^1^, Amanda Odoi^1^, and Sarah D. Rominski^2^

^1^University of Cape Coast, Cape Coast, Ghana

^2^University of Michigan, Ann Arbor, Michigan, USA

**Purpose:** Sexual violence (SV) is a significant challenge for universities worldwide, affecting the health, well-being, and academic success of students. Although SV prevention programs are common at universities in the United States, they are limited in Ghana. The purpose of this study was to systematically adapt and then evaluate a SV prevention program among students at the University of Cape Coast (UCC) in Ghana.

**Method:** The ADAPT-ITT framework was used to guide the adaptation, and the research team worked with the UCC gender center, students, and faculty throughout the process. Adaptation methods included (a) four focus groups with students (*n* = 26) and 20 cognitive interviews to validate measures (February 2016), (b) beta testing of the adapted program with UCC students on two occasions (*n* = 76 in April 2016 and *n* = 57 in September 2016), (c) review by topical experts on two occasions (July and October 2016), (d) integration of feedback into the final adapted program (November 2016), and (e) training of peer facilitators (*n* = 10; March 2017). In August 2019, all incoming students were recruited from their residence halls for scale-up testing. The adapted program was delivered by the trained peer facilitators and participants completed a pre- and post-program survey to capture demographics, knowledge of resources at UCC, attitudes toward SV and gender equity, and behavioral items on victimization and perpetration from the World Health Organization Violence Against Women study (2005).

**Results:** During the beta testing phase, this program demonstrated promise in changing attitudes related to rape myth acceptance and gender equity, which are known to be associated with SV perpetration. A total of 3,500 students participated in the scale-up testing. Data analysis is currently underway.

**Implications:** A primary prevention intervention initiated in a college-age population has the potential to disrupt SV that may precipitate chronic physical, psychological, and reproductive health consequences and disrupt women’s access to a university education. Utilizing a systematic adaptation and scale-up process has the potential to provide a framework for other universities in sub-Saharan Africa to adapt and scale-up primary prevention programs for their student populations.

## Co-Development of Intervention Recommendations to Promote Screening for Traumatic Brain Injuries at Women’s Shelters Using Behavioral Science

### Blake Nicol^1^, Paul van Donkelaar^1^, Karen Mason^2^, Kennedy Louis^1^, and Heather L. Gainforth^1^

^1^University of British Columbia–Okanagan, Kelowna, British Columbia, Canada

^2^Kelowna Women’s Shelter, Kelowna, British Columbia, Canada

**Introduction:** Women who experience intimate partner violence are at a high risk of traumatic brain injuries (TBIs). Screening for TBIs could improve the TBI support they receive. Women’s shelters may be an ideal location to screen women who have experienced intimate partner violence–caused TBIs.

**Purpose:** To use the Behavior Change Wheel and the Theoretical Domains Framework (TDF) to co-develop intervention recommendations to promote screening of TBIs at women’s shelters.

**Study Design:** Mixed-methods study conducted in three phases.

**Data Collection and Sample:** Phase I was a Canada-wide survey study using the TDF to understand the barriers staff experience toward screening their clients for TBIs. Phase II consisted of interviews with staff members in Kelowna, British Columbia, to further understand barriers within a local context using a semi-structured TDF interview guide. Phase III comprised the co-development of the intervention recommendations to promote screening of TBIs by the research team and the executive director of the Kelowna Women’s Shelter.

**Analysis:** The survey and interview data were deductively coded using the TDF and inductively using thematic analysis. Recommendations were co-developed through discussion.

**Results:** Phase I participants (*n* = 150) were likely not screening for TBIs due to a lack of skills, lack of knowledge, nervousness about screening, lack of resources, and social influences from their clients. Phase 2 participants (*n* = 10) were likely not screening for TBIs due to a lack of knowledge, beliefs about their capabilities, and a lack of resources. Based on the results from Phases I and II, five intervention recommendations were co-developed in Phase III to promote screening of TBIs in women’s shelters: (a) establish formal policies and procedures requiring clients to be assessed for TBIs, (b) provide training to staff who work in women’s shelters, (c) assess for TBIs in a conversational style and not at intake, (d) educate clients about TBIs, and (e) develop a referral system for clients at risk for a TBI.

**Implications:** This study is the first to use behavioral science to co-develop intervention recommendations promoting screening of TBIs at women’s shelters. The recommendations may improve the brain injury support women receive, and ultimately could improve their quality of life.

## Methodological and Ethical Considerations in Research With Immigrant and Refugee Survivors of Violence

### Veronica P. S. Njie-Carr^1^, Bushra Sabri^2^, Jill Theresa Messing^3^, Allison Ward-Lasher^3^, Crista Johnson-Agbakwu^4^, Catherine McKinley (formerly Burnette)^4^, Nicole Campion Dialo^1^, Saltanat Childress^5^, Joyell Arscott^2^, and Jacquelyn C. Campbell^2^

^1^University of Maryland, Baltimore, Maryland, USA

^2^Johns Hopkins University School of Nursing, Baltimore, Maryland, USA

^3^Arizona State University, Phoenix, Arizona, USA

^4^Maricopa Integrated Health System, Phoenix, Arizona, USA

^5^University of Texas at Arlington, Austin, Texas, USA

Promoting the health of immigrants and refugees globally continues to be a priority for the World Health Organization. In 2017, 258 million people were international migrants, a 49% increase over 17 years. Of this total, more than 43 million immigrants and refugees are living in the United States substantiating the need for quality and culturally appropriate evidence-based interventions to better understand their unique health needs across geographic borders. Immigrant and refugee women experience intimate partner violence at a higher rate with more serious negative health-related disparities than women in the host country. The purpose of this paper is to discuss the strategies employed in enrolling immigrant and refugee women participants for the weWomen Study (https://wewomen.nursing.jhu.edu/home) as the context for analyzing the methodological and ethical challenges encountered. Numerous challenges were encountered in the recruitment and retention of these “hidden” populations, including women survivors’ fear of deportation, linguistic barriers, and mistrust of providers and researchers. We utilized a multi-faceted approach informed by best practices to maximize participant enrollment, which can be replicated in similar studies. Effective strategies that include allocating adequate budget and actively engaging community collaborators through community-based participatory approaches to maximize recruitment and retention efforts are discussed. While the challenges the team experiences might have seemed insurmountable, their commitment to ensuring the integration of the unique needs of immigrant and refugee women survivors resulted in higher enrollment numbers. Immigrant and refugee survivors can contribute valuable information to inform culturally appropriate targeted evidence-based interventions to promote positive health outcomes.

## Health Professionals’ Perceptions of How Gender-Sensitive Care Is Enacted Across Acute Psychiatric Inpatient Units for Women Who Are Survivors of Sexual Violence

### Carol O’Dwyer^1^, Laura Tarzia^1,3^, Sabin Fernbacher^3^, and Kelsey Hegarty^1,3^

^1^The University of Melbourne, Melbourne, Victoria, Australia

^2^Consultant, Melbourne, Australia

^3^The Royal Women’s Hospital, Melbourne, Victoria, Australia

**Problem Statement (describe problem/issues addressed and its significance):** Sexual violence is a global public health issue. Women with mental illness frequently experience sexual violence. The biomedical model has been the dominant model of care in psychiatric inpatient units. Recent years have seen a global movement toward gender-sensitive and trauma-informed models of care. To date, only a small amount of research has focused on evaluating these models of care and health professionals’ experiences of providing this care.

**Purposes and/or Questions/Hypotheses:** The aim of this study is to gain in-depth understanding of health care professionals’ perceptions of how gender-sensitive care (GSC) is enacted across psychiatric inpatient units for women who are survivors of sexual violence.

**Study Design:** This study used case study methodology and the Normalization Process Theory (NPT) conceptual framework.

**Sample:** Participants included 40 health professionals of different occupational groups, including medical, nursing, and allied health staff of both genders.

**Data Collection Approach:** It included semi-structured interviews, document and policy reviews, and observations from four psychiatric inpatient units within a large Australian public mental health organization. This presentation will report on the findings from the interviews.

**Analysis:** Thematic and content analysis were used to analyze the data.

**Results:** The themes were developed under the four NPT core constructs: (a) Understanding GSC in psychiatric inpatient units: “Without the corridors, there’s not a lot we can do”; (b) Engagement and commitment to GSC: “There are a few of us who have that gender-sensitive lens”; (c) Organizing, relating, and involvement in GSC: “Its band-aid stuff”; and (d) Monitoring and evaluation of GSC: “We are not perfect, we have to receive that feedback.” Health professionals in this study enacted GSC to varying levels and showed avoidance of responsibility to implement it. In addition, the competing demands of the biomedical model and a minimal appraisal have resulted in an inconsistent enactment of GSC.

**Implications or Lessons:** Our findings suggest the need to address each NPT construct comprehensively to adequately implement GSC. These findings contribute to improving the provision of high-quality, gender-sensitive care for female consumers during their admissions to psychiatric inpatient units.

## Nurses Involvement in the Management of Family Violence Victims in Ondo State, Nigeria

### Oluwasayo Bolarinwa Ogunlade^1^, Adekemi Eunice Olowokere^1^, Ojo Melvin Agunbiade^1^, and Omolola Irinoye^1^

^1^Obafemi Awolowo University, Ile-Ife, Nigeria

Family violence is a public health issue with its effects on every member of the family. Recognizing primary health care settings as ideal settings to respond to victims of family violence with nurses is strategic in Nigeria. Nevertheless, there is limited information on the involvement of nurses in the detection and response strategies for the management of family violence. Hence, the study assessed primary health care nurses’ processes to identify and respond to family violence in a state in Nigeria. A descriptive qualitative design was employed with a face-to-face interview conducted with six nurses in purposely selected primary health centers. Thematic analysis of the interviews was used to define key issues and areas of interest as identified by participants. Nurses mean age was 33.67 ± 5.849 years and mean years of experience 10 ± 3.45 years. Results showed that victims of physical violence were the only victims identified but without any identification or response guidelines. Nurses were not involved in safety planning and risk assessment. Nurses respond by treating the sustained injuries, counsel the victim, and mediate with the couple. The findings gave implication for further education and training of nurses and advocacy for collaborative policies and utilizing community resources to support women and children victims of family violence.

## Intimate Partner Homicides Perpetrated in West Sweden 2000–2016—A Process Perspective: The Stop Study

### Karin Örmon^1,2^, Gunilla Krantz^1,3^, Henrik Lysell^1,4^, and Viveka Enander^1,3^

^1^The Västra Götaland Competence Centre on Intimate Partner Violence, Gothenburg, Sweden

^2^University of Malmö, Malmö, Sweden

^3^University of Gothenburg, Gothenburg, Sweden

^4^Karolinska Institute, Stockholm, Sweden

**Introduction:** The rationale for focusing on intimate partner homicides (IPHs) a process perspective was that many risk factors for IPH have been described in previous research, but it may be difficult put these into context. In many cases, several risk factors can be found, but which are most pertinent of the time of the crime? A process perspective is valuable in understanding IPHs, from the building up to the aftermath of the killing.

**Method:** The present study is part of the STOP study, which is a qualitative study describing intimate partner homicides perpetrated in West Sweden 2000–2016. The result of this study is based on 50 court files, 40 male and 10 female perpetrators. The research question are as follows: What does the building up for the killing entail? When and why does the killer decide to kill? and finally, What does the aftermath of the deadly violence look like? The court files are analyzed with thematic analysis.

**Results:** We discerned a process in which the borders between the buildup, the killing, and the aftermath are blurred. Even so, are some distinctive features of the process explicit. Possessiveness and mental ill health are, for example, identified throughout the process, ending up with the murder of an intimate partner. There is also a distinct difference between male and female perpetrators of intimate partner homicide.

**Conclusion:** A process perspective is valuable in understanding IPHs.

## Risk of Vicarious Trauma for Graduate Student-Researchers Exploring Sensitive Topics: A Scoping Review of Canadian Dissertations and Theses

### Elizabeth Orr^1^, Pamela Durepos^1^, Vikki Jones^2^, and Susan Jack^1^

^1^McMaster University, Hamilton, Ontario, Canada

^2^York University, Toronto, Ontario, Canada

**Problem Statement:** Qualitative research concerning sensitive topics (i.e., violence, trauma, abuse, etc.) is emotionally demanding for all parties involved. While it is common for ethics protocols to protect research participants from emotional distress, the personal impact of emotional work on the researcher can often go unaddressed. Qualitative researchers, in particular, graduate student-researchers studying sensitive topics, are at risk for vicarious trauma or emotional impact resulting from engagement in this type of work. It is unclear, however, how this researcher impact is discussed in graduate student work and/or the steps taken to address this risk.

**Purpose:** The purpose of this study is to provide an overview of how researcher impact or vicarious trauma is considered in Canadian graduate student research.

**Study Design:** A comprehensive scoping review of dissertations and theses was conducted. The scoping review framework outlined by Arksey and O’Malley (2005) was used to guide this review.

**Sample:** Canadian dissertations and theses published to their university repository with the last 10 years (dates: January 2009—May 2019) and meeting pre-determined inclusion criteria formed the sample for this review.

**Data Collection:** The ProQuest Dissertations & Theses database was used to search for student research work relevant to the study purpose. A hand search of the dissertation repositories within the top 10 Canadian universities was also performed.

**Analysis:** More than 50,000 titles were reviewed with 562 retained for data extraction. Data extraction items included institution, department, publication year, degree, sensitive topic considered, and evidence of methods to address the emotional impact of sensitive research on the student-researcher.

**Results:** Final analysis to be completed by December 2019. Interim findings show that 81.5% of dissertations do not discuss methods to address the emotional impact of sensitive research on the student-researcher; 15.2% of dissertations describe some strategy (predominantly journaling) for documenting the impact of the research and only 3.3% of dissertations had a defined protocol or plan to address the risk of emotional distress or vicarious trauma on the student-researcher.

**Implications:** Findings suggest the need for further guidance on minimizing the risk of emotional distress among student-researchers using qualitative methods within sensitive topic areas.

## Can Trauma- and Violence-Informed Care Promote Change? Early Insights From a Critical Ethnography of Nurses Working With Women Who Have Experienced Intimate Partner Violence

### Noël Patten Lu^1^, Marilyn Ford-Gilboe^1^, Lorie Donelle^1^, Victoria Smye^1^, and Kimberley Jackson^1^

^1^Western University, London, Ontario, Canada

**Background and Purpose:** As the first, and possibly the only, point of contact in the health care system for women who have experienced intimate partner violence (IPV), nurses can have profound effects (positive and negative) on women’s health, safety, and how they engage with health services. However, numerous studies demonstrate that health care providers, including nurses, are often not well equipped to deal with IPV and lack appropriate training, which can contribute to further harms and re-traumatization. One possible path to better care is to support nurses to take up Trauma- and Violence-Informed Care (TVIC), an emerging approach that has not yet been extensively studied or widely implemented. Consequently, the potential of TVIC for nursing practice with women who have experienced interpersonal and structural violence is not well understood. The purpose of this study was to explore how nurses experience, come to understand, and take up the concept of TVIC over time in the context of practice with women who have experienced IPV.

**Methodology:** In this critical ethnography, key informants are 12 nurses hired and trained to offer the Intervention for Health Enhancement and Living (iHEAL), a community-based health promotion intervention for women in the transition of separating from an abusive partner. As part of their role, these nurses received standardized education about TVIC and how to integrate it into practice, along with support from a clinical supervisor. Repeat, in-depth qualitative interviews are being conducted with these participants at three points in a 16-month period to capture their experiences over time. Transcribed interviews are being analyzed using thematic analysis.

**Findings and Implications:** Preliminary findings based on 12 first and second interviews suggest that, in spite of the promise of TVIC, nurses experience multiple tensions and challenges in adopting TVIC in the context of practice norms that emphasize efficiency in treatment of individualized problems and overlook structural factors that shape women’s health. Analysis will be completed in spring 2020. Findings of this research will offer insights into the complexity of TVIC and strategies that could support the successful adoption of this promising approach to care.

## ENGAGE—Roadmap for Frontline Professionals Interacting With Male Perpetrators of Domestic Violence and Abuse to Ensure a Coordinated Multiagency Response to Perpetrators

### Alessandra Pauncz^1^ and Geldschläger Heinrich^2^

^1^Psychologist, WWP EN

^2^Clinical Psychologist, Connexus, Spain

Domestic violence and abuse against intimate partners have a devastating impact on the health and well-being of the victims and the perpetrator, with long-term negative consequences for all involved. Adequate measures to protect victims are essential, yet a comprehensive policy to tackle this kind of violence must also address the perpetrators. The Council of Europe Convention on preventing and combating violence against women and domestic violence (Istanbul Convention) requires European Union Member States to invest in programs for domestic violence perpetrators and for sex offenders.

Within the framework of addressing perpetrators and increasing their referral to perpetrator programs a consortium of six European organizations and institutions (WWP EN, Connexus, CAM, Terres des Hommes, Psytel and the City Council of Florence) developed the project ENGAGE, within the frame of the European project REV-VAW-2016, to assist through a roadmap and a training package frontline professionals in health care or social services, child protection services, police, and others, coming into contact with male service users who are violent or abusive to their female partners. One of the most common requests from victims is for someone to work with their partner, to help him change, and to keep them and their children safe from violence. Working with these men to change their behavior is a key step toward preventing domestic violence. Responses of frontline health care professional to any disclosure, however indirect, could be significant for encouraging responsibility and motivating men toward change. It is paramount to keep in mind that the primary goal of all work with male perpetrators (including identification and referral) is to ensure the safety of women and children. The presentation will share the tools and experiences of the development of the project. Participants will be guided through the theoretical background and the practical tools that were developed in the course of the project.

## Effectiveness of a Trauma-Informed IPV Training Intervention for Health Providers in Improving IPV Discussions With Patients

### Nancy Perrin^1^, Liz Miller^2^, Lisa James^3^, Surabhi Kukke^3^, Amber Clough^1^, Karen Grace^1^, Annie Lewis O’Connor^4^, and Nancy Glass^1^

^1^Johns Hopkins University School of Nursing, Baltimore, Maryland, USA

^2^University of Pittsburgh, Pittsburgh, Pennsylvania, USA

^3^Futures Without Violence, San Francisco, California, USA

^4^Brigham and Women’s Hospital, Boston, MA, USA

Intimate partner violence (IPV) is seldom addressed in primary health care settings. Both screening and patient education for IPV occur at low rates due to a lack of training for health care providers and referral pathways for women who experience IPV. The PATHS study evaluated the effectiveness of a trauma-informed IPV training intervention for providers.

**Method:** Fifteen clinics were randomly assigned to the intervention or delayed control group. Women with an appointment at the clinic were invited to participate in the study which included completing a survey prior to seeing their provider and a survey after their visit about their interaction with the provider. The pre-intervention surveys were completed by 1,591 women in the control and 1,782 in the intervention group. In the post-intervention period, 1,034 control and 1,865 intervention women completed the survey.

**Findings:** The percent of women who reported that her health care provider discussed healthy and unhealthy relationships with her increased from the pre- to post-period (27.1% to 35.9%) in the intervention group while there was little change (34.4% to 35.1%) in the control group (*p* = .001). A similar pattern was found for discussing the impact of violence on health (intervention group: 16.9% to 25.3%, control group: 22.9% to 24.3%, *p* < .001). Approximately 16% of the women screened positive for IPV on the pre-visit survey. The percent of women disclosing IPV to her provider increased significantly in the intervention group (19.6% to 25.7%) compared with the control group (28.5% to 30.4%, *p* = .012). However, the rate of referrals for women who disclosed IPV did not change after the intervention in either the control group (44.6% to 45.2%) or the intervention group (44.4% to 42.5%).

**Conclusion:** Trauma-informed IPV training for providers increases the likelihood that providers will discuss IPV and its impact on health with their patients. These discussions appear to increase the number of women who disclose their experiences of IPV to the health care provider. However, additional approaches are needed to strengthen the referral pathway for women experiencing IPV.

## The Intersection of Culturally Competent Primary Care and Domestic Violence: An Interpretive Synthesis

### Bijaya Pokharel^1^, Jane Yelland^2,3^, Ann Wilson^1^, Sandesh Pantha^1^, and Angela Taft^1^

^1^La Trobe University, Melbourne, Victoria, Australia

^2^Murdoch Children’s Research Institute, Parkville, Victoria, Australia

^3^The University of Melbourne, Melbourne, Victoria, Australia

**Problem Statement:** Primary care providers in Australia are encouraged to recognize and provide appropriate care to women who experience domestic violence. Almost half the Australian population were either born overseas or had at least one of their parents born overseas. As a result, primary care needs to be responsive to the needs and expectations of culturally and linguistically diverse populations. However, little is known about the intersection of cultural competency, primary care, and domestic violence. Although Australia has an increasing emphasis on culturally competent health care, there is a paucity of understanding on what that might look like in the primary care context of domestic violence.

**Purpose of the Study:** This review aims to understand the meaning of cultural competency in the primary care context of women of immigrant and refugee backgrounds who experience domestic violence.

**Design and Method:** We will use critical interpretive synthesis for this review. To date, we have searched eight databases and the websites of 15 Australian governmental and non-governmental organizations, but we will present final results. We will use citation tracking and expert consultation as additional search strategies. We will include studies that focus either on woman above 16 years who experience domestic violence or on primary care providers. The studies will focus on interaction between women and primary care providers. Two reviewers will independently screen title and abstracts of the studies retrieved from search results. Two reviewers will independently assess the quality using the Crowe Critical Appraisal Tool (CCAT; quantitative and mixed-methods studies), Quality Framework (qualitative studies), and Authority, Accuracy, Coverage, Objectivity, Date, and Significance (AACODS; gray literature). We will use data extraction tables to extract and summarize data on study context, components of cultural competency, and their process of delivery to women of immigrant and refugee backgrounds who experience domestic violence.

**Implications:** This study will propose an evidence-informed model of culturally competent primary care to women of immigrant and refugee backgrounds who experience domestic violence. The model can inform primary care providers on essential components of culturally competent care and how to effectively deliver these to women.

## Differences in Help-Seeking Behaviors and Perceived Helpfulness of Services Between Abused and Non-Abused Women

### Leesa Hooker^1^, Leonie Versteegh^2^, Helena Lindgren^2^, and Angela Taft^1^

^1^La Trobe University, Melbourne, Victoria, Australia

^2^Karolinska Institutet, Stockholm, Sweden

**Background:** New mothers may face substantial health challenges during the postpartum period and are at greater risk of intimate partner violence. Health care services provide support; however, acknowledging a problem and seeking help for it can be difficult. Research on where postpartum women tend to seek help and how helpful they perceive it is limited. In addition, little is known of how these help-seeking behaviors differ between abused and non-abused postpartum women.

**Objective:** To examine the help-seeking behavior and perceived helpfulness of services in abused and non-abused postpartum women.

**Method:** Secondary analysis of data collected during the MOVE (Improving Maternal and Child Health Care for Vulnerable Mothers) trial of nurse intimate partner violence screening and supportive care. The MOVE study was a cluster randomized controlled trial that included a survey of *N* = 2,621 postpartum Australian women who had given birth within the previous 8 months. Data were analyzed using descriptive and inferential statistics.

**Results:** Abused women sought informal family support less frequently (*p* < .001) and were more frequent users of emergency departments (*p* = .03), home visiting programs (*p* = .02), and breastfeeding services (*p* = .001), compared with non-abused women. These women were also more frequent users of psychiatrists (*p* ≤ .001); early parenting centers, both day-stay (*p* = .006) and residential (*p* = .008); and child welfare services (*p* < .001), and they were generally less satisfied with the help received.

**Conclusion:** Postpartum women experiencing partner violence seek help from certain formal services more frequently and are less satisfied with the care received, compared with non-abused women. Access to potential protective supports from family and friends is limited. To improve health care services, further qualitative research is needed to enhance our understanding of abused postpartum women’s experiences and help-seeking behaviors.

## Trauma- and Violence-Informed Care in the K-12 Classroom: Teacher Education

### Susan Rodger^1^ and Nadine Wathen^1^

^1^Western University, London, Ontario, Canada

Schools play a key role in providing both healthy environments where students can learn and develop and receive universal and targeted mental health support; indeed, the research evidence converges to suggest that academic engagement, learning, and belonging requires mental, physical, and relational health. This paper will describe the results of bringing a trauma- and violence-informed care approach to K-12 education, providing a framework of practice that enables schools to be safe, inclusive places for some of the most vulnerable students, including those exposed to interpersonal and structural violence. Students exposed to trauma and violence can struggle to attend, engage, and achieve at school. It is critical that students affected by trauma and violence receive appropriate and equitable support, so that they can thrive in school. National research has demonstrated that the majority of teachers feel ill-equipped to meet the mental health and inclusion needs of their students. Providing teacher education students with education about trauma, TVIC, and the ways the trauma can influence academic engagement and success may allow problematic classroom behaviors to be viewed through a health equity and inclusive education lens, promoting both the creation of a safer and more equitable learning environment for students directly affected by trauma and violence and supporting the ethic of care that is central to the helping professions, including teaching. Initial teacher education may provide the opportunity to prepare teachers with the knowledge, strategies, and self-efficacy in TVIC necessary to create classrooms and learning experiences that are safe, equitable, and meet the needs of all students. A mandatory, completely online mental health course for second-year students in a Bachelor of Education program (*n* = 235) at a large Canadian university introduced TVIC concepts and used a case study approach to articulate challenges for students and strategies, tools, and knowledge for teachers. Results indicate significant changes in participants’ attitudes toward TVIC and their self-efficacy in using inclusive teaching practices, as well as their expressions of intentions to create safe and welcoming classrooms and schools aligning with TVIC principles, providing support for the inclusion of these important topics for all teacher education students.

## Impact of Sources of Strengths on Coping and Safety of Immigrant Survivors of Intimate Partner Violence

### Bushra Sabri^1^, Veronica Njie-Carr^2^, Sarah Murray^3^, Joyell Arscott^1^, and Jacquelyn C. Campbell^1^

^1^Johns Hopkins University School of Nursing, Baltimore, Maryland, USA

^2^University of Maryland, Baltimore, Maryland, USA

^3^Johns Hopkins Bloomberg School of Public Health, Baltimore, Maryland, USA

**Background and Purpose:** Immigrant women are disproportionately affected by intimate partner violence (IPV), severe IPV, and homicides. Immigrant women face “triple jeopardy” related to their gender, minority race/ethnicity, and immigrant status. Despite experiences of IPV, internal and external sources of strengths can promote resilience or influence immigrant women’s ability to deal with their abusive relationships. These sources of strengths can be found at cultural, community, relationship, and individual/survivor levels. While prior research has focused on risk factors only or resilience factors in only some groups of survivors, studies have not examined sources of strengths and their variations among diverse groups of immigrant survivors. Drawing from resilience/strength’s perspective, this study explored external and individual sources of strengths among diverse groups of immigrant survivors and examined how these sources influenced their safety and coping with abuse.

**Method:** Data for this qualitative study were collected from immigrant women residing in Massachusetts, Arizona, Virginia, Washington, D.C., New York, Minnesota, and California using purposive and snowball sampling techniques. Seventy-nine in-depth interviews were conducted with adult immigrant survivors of IPV, who identified as Asian (*n* = 29), Latina (*n* = 27), and African (*n* = 23). Data were analyzed using thematic analysis procedure.

**Results:** Women identified both external (e.g., community support, formal sources of help) and internal (e.g., optimism, faith, beliefs) sources of strengths. Some survivors had mixed experiences with some sources (e.g., religious institutions). However, given the importance of these sources in assisting survivors, the study highlights areas of improvement and how these sources can adequately address needs of survivors.

**Implications:** The findings are informative for practitioners serving immigrant survivors of IPV in legal, social service, and medical and mental health settings. Practitioners can reflect on protective actions that the immigrant survivor has taken and how to capitalize on their strengths. With growing immigrant populations in the United States, practitioners are more likely to encounter survivors from diverse countries of origin and would need to provide culturally informed safety planning services. Identifying sources of strengths and their roles in women’s safety or coping can be useful for the development of strengths-based intervention strategies for immigrant survivors of IPV in the United States.

## Contextual Factors Related to Violence by Partners and In-Laws and Needs for Interventions at Multiple Levels: A Study of Women in India

### Bushra Sabri^1^

^1^Johns Hopkins University School of Nursing, Baltimore, Maryland, USA

**Background and Purpose:** India suffers a high burden of violence against women. In addition to abuse perpetuated by an intimate partner, abuse perpetuated by in-laws is a pervasive phenomenon among Indian families. Using a socioecological framework, this study identified women’s perceptions of contextual factors related to abuse by intimate partners and in-laws and areas of interventions at multiple levels.

**Method:** Twenty-seven in-depth interviews were conducted with participants in two cities in North India. Participants were recruited utilizing purposive and snowball sampling techniques. The strategies included flyers, word of mouth, and assistance from a women’s health clinic providing mother and child health care services to women in the community. Data were collected utilizing in-depth, semi-structured interview guides, with questions focusing on perceptions and experiences of abuse and strategies and resources used for coping, safety concerns, and health. The quantitative measures were used to collect information such as age, income, marital status, children, level of education, and health. Data analysis utilized a theoretical thematic analysis procedure underpinned by the socioecological framework aimed at identifying context of abuse, sources of help, and recommendations for services.

**Results:** Analysis of qualitative data revealed contextual factors related to abuse by husband and in-laws and needs for services at the societal, community, and relationship levels. Reasons for exposures to violence at the societal/cultural level included patriarchal cultural norms, non-arranged marriages, dowry expectations, stigma of being remarried, gender role expectations, and preference for a male child. The community-level reasons for violence exposure included instigation of violence by outsiders in the community and limited access to services. Reasons for abuse at the relationship level included disclosing abuse to parental family, instigation of abuse by in-laws, and characteristics of both the in-laws and the partner (e.g., alcohol problems, anger issues, suspicious behaviors, jealousy, and financial issues/unemployment). Needs for interventions include areas such as government support, knowledge and awareness about resources, and programs for women’s education and economic independence. The findings can be used for creating prevention and intervention strategies that target contextual factors at multiple levels that support violence perpetrated by intimate partners and in-laws.

## Community Stakeholders’ Acceptability of Life Skill Building Intervention for Women Empowerment in Pakistan

### Tazeen Saeed Ali^1**^, Rozina Karmaliani^1^, Judith McFarlane^2^, Hussain Maqbool Ahmed Khuwaja^1^, Shireen Shehzad Bhamani^1^, Nasim Zahid Shah^1^, Zahid Hyder Wadani^1^, and Asli Kulane^3^

^1^Aga Khan University, Karachi, Pakistan

^2^Texas Woman’s University, Houston, Texas, USA

^3^Karolinska Institutet, Stockholm, Sweden

**Background:** Violence against women (VAW) is the major determinant of women’s mental health in Pakistan. Many interventions to reduce VAW have been tested but the documentation of men’s role toward women’s empowerment is lacking at local context. A qualitative approach was undertaken in all four provinces of Pakistan to describe the community residents’, men’s and women’s, perceptions regarding the acceptability of a proposed Life Skills Building (LSB) intervention that involves men’s engagement toward women’s empowerment.

**Method:** Qualitative inquiry using 18 Focus Group Discussions, nine with community males (*n* = 88) and nine with community females (*n* = 79), with an average of six to 10 participants for a duration of 60 to 90 minutes and 14 Key Informant Interviews consisting of 10 males and four females for a duration of 30 to 45 minutes were completed. All the interviews were conducted in four pre-dominant provincial languages of Pakistan. The data analysis was done using the thematic analysis approach.

**Result:** The study identified three major themes, which are Family Life issues, Importance of LSB, and feasibility of LSB implementation. The proposed LSB intervention was well accepted with strong urge to engage men in receiving the training. Participants suggested that these sessions should be held 2 hours per week, within community public spaces. In addition, these sessions should be based on a participatory and interactive approach, where the participants are motivated to attend all the proposed sessions with a special focus on positive relationship and economic skills building.

**Conclusion:** Women, men, and community stakeholders affirmed that the community members would accept the LSB intervention. Involvement of men is important and this was recognized as an important element which could facilitate in women empowerment and support to reduce violence, and improve mental health of women in a patriarchal country like Pakistan.

**Keywords**

mental well-being, domestic violence, LSB, women empowerment

## Community Stakeholders’ Views on Strategies for Reducing Violence Against Women in Pakistan

### Tazeen Saeed Ali^1^, Rozina Karmaliani^1^, Hussain Maqbool Ahmed Khuwaja^1^, Zahid Hyder Wadani^1^, Nasim Zahid Shah^1^, Saher Aijaz^1^, and Asli Kulane^2^

^1^Aga Khan University, Karachi, Pakistan

^2^Karolinska Institutet, Stockholm, Sweden

**Background:** Nearly half of the women experience violence across their lifespan in all the provinces of Pakistan. Despite knowing the prevalence, there has been meager progress in developing strategies to combat violence at individual, family, or community level. Many interventions suggested in other countries have been pilot tested, but the effects of those interventions had been limited. Therefore, the aim of this study is to understand the voices of stakeholders to reduce violence against women and to explore the possible community-based strategies that could be implemented in Pakistan.

**Method:** A total of 14 key informant interviews and 18 focus group discussions were held across all four provinces of Pakistan. Participants were purposefully recruited and all the interviews were audio-recorded. Transcriptions were open coded and thematic analysis was done to extract themes with similar meanings.

**Results:** Community members shared five key strategies for reducing violence against women which included the need of raising voice against the status quo, advocating the role of education in women empowerment, promoting women rights campaigns across Pakistan, bringing innovations in existing interventions across all life stages, and strengthening laws that could promote women empowerment.

**Conclusion:** Violence against women is known to be linked with the cultural norms, so community stakeholders’ involvement and participation is essential to bring change. The responsibility to bring about a substantial change in behavior and attitudes of men must begin with engaging them in all the interventions that aim to reduce violence. Therefore, there is a dire need to implement the interventions that are locally planned and scalable within the existing system in Pakistan.

**Keywords**

violence against women (VAW), violence reduction strategies, men engagement, stakeholders’ voices for VAW

## Understanding Common Themes in International Research: How Narrative Interviewing Helps Us Understand Key Trauma Recovery Processes

### Denise Saint Arnault^1^, Laura Sinko^2^, Maddalena Rodelli^3^, Ines Testoni^3^, Francesca Alemanno^3^, and Sachiko Kita^4^

^1^University of Michigan, Ann Arbor, Michigan, USA

^2^University of Pennsylvania, Philadelphia, Pennsylvania, USA

^3^Università degli studi di Padova, Padova, Italy

^4^University of Tokyo, Tokyo, Japan

International research has shown that trauma recovery is often blocked by cultural norms that blame survivors, shame them for leaving, minimize the significance of violence, or normalize violence. The Multicultural Study of Trauma Recovery (MiStory) is an international collaboration network that is using narrative interviewing to understand the ways that internalized cultural beliefs and social processes shape the ways that gender-based violence (GBV) survivors make meaning of their experiences and heal from them. This symposium will bring together four papers that explore interacting themes that we have found in our research. We will save ample time for discussion and reflection from the audience.

**Paper 1:** The Clinical Ethnographic Narrative Interview as a Cross Cultural Tool to Safely Examine Trauma Recovery (Denise Saint Arnault, University of Michigan, Ann Arbor, USA). Research has shown that narrative methods can be helpful in providing trauma survivors a vehicle to promote their recovery. However, trauma-informed practice has taught us that re-traumatization is a real risk in research. The MiStory project aims to document culturally universal and culturally specific processes of trauma recovery to inform practice and policy, and we are using the Clinical Ethnographic Narrative Interview (CENI) method as a way to provide women from widely different cultural backgrounds a safe way to explore their recovery journey. This presentation explains trauma and narrative theory and describes the CENI interview.

**Paper 2:** Normalization of Sexual Violence on an American College Campus (Laura Sinko, University of Pennsylvania, Philadelphia, Pennsylvania, USA). While literature documents how sexual violence (SV) normalization relates to SV perpetration in the American undergraduate university setting, little is known about how SV normalization (SVN) is perceived by SV survivors. The purpose of this study was to describe how survivors recognize SVN on college campuses and to understand how SVN affects meaning-making after unwanted sexual encounters. Twenty-three survivors of Unwanted Sexual Experiences participated in the CENI, and grounded theory analysis found that survivors recognized SVN through responses to SV disclosure, witnessing or experiencing repeated SV, observing campus values surrounding gender and hook up norms, and retrospectively noticing difficulties processing their own experiences. SVN seemed to exacerbate survivors’ symptom burden and negative self-concept, affecting meaning-making through labeling difficulties, personal responsibility confusion, and social disconnection. Results highlight the impact of sociocultural norms on SV recovery.

**Paper 3:** After the Violence: Empowerment Processes in Women Who Experienced Gender-Based Violence (Maddalena Rodelli, Ines Testoni, & Francesca Alemanno, Università degli studi di Padova, Padova, Italy). Research has identified empowerment to be a central goal in the trauma recovery, going beyond the intra-psychic dimension of suffering, addressing the feelings of capacity to address the power dynamics and patriarchal aspects of gender relationships. We conducted the CENI with 15 women of Latin America origin who migrated in Italy and Spain. Our intersectional qualitative analytic frame examined how women understood power dynamics, how they defined their empowerment processes, and their emerging ability to address power dynamics during their recovery. Results suggest that there is a need to consider women as active, tenacious agents of personal and social change, instead of passive and vulnerable victims. Our findings illuminate the re-victimization processes are often part of the welfare approach in social and mental health services. We conclude that women are seeking services that promote empowerment, active involvement, and self-determination.

**Paper 4:** Journey to Trauma Integration: Re-Trusting, Re-Building, and Re-Embracing Selfhood, Life and the World (Sachiko Kita, University of Tokyo, Tokyo, Japan). For domestic violence survivors, integrating trauma into their selfhood is a long and challenging journey. We conducted the CENI with 23 Japanese survivors to identify the phases of trauma integration and factors promoting it. We then identified a subset of 11 women who described that they had achieved integration, and used grounded theory to discover the processes that characterized their integration journey. The results revealed six phases: “confusion,” “overwhelmed,” “awareness,” “fighting,” “overlooking,” and “integration.” Other critical recovery tasks included “rebuilding the boundary between myself and others” and “trusting others and seeking help again.” These recovery tasks decreased symptoms, promoted autonomy, and enhanced understanding and acceptance of trauma. Implications for practice include understanding the complexity of the trauma integration processes, the skills necessary to achieve it, and possible cultural differences.

## Effect of a Psychosocial Intervention on Mental Health, Social Support, and Help-Seeking Behaviors Among Abused Pregnant Women: A Pilot Randomized Controlled Trial in Nepal

### Diksha Sapkota^1,2,3^, Kathleen Baird^1,4^, Amornrat Saito^1,3^, and Debra Anderson^1,3^

^1^Griffith University, Gold Coast, Queensland, Australia

^2^Kathmandu University, Dhulikhel, Nepal

^3^Menzies Health Institute Queensland, Brisbane, Queensland, Australia

^4^Gold Coast University Hospital, Gold Coast, Queensland, Australia

**Methods and Analysis:** A two-arm parallel-randomized controlled trial (RCT) was conducted among pregnant women attending antenatal clinic of a tertiary hospital of Nepal. In total, 140 women were randomly allocated to either an intervention group or a control group (70 in each group). Intervention participants received a counseling session, an information booklet, and contact details of the counselor, while control group participants received referral list of local support organizations working against domestic and family violence (DFV). Outcome measures, such as mental health, quality of life, social support, and help-seeking behaviors were measured at baseline, 4 weeks post-intervention, and 6 weeks postpartum. The generalized estimating equation (GEE) model using intention-to-treat (ITT) approach was used to examine change in outcome variables over the time in two groups using IBM SPSS Statistics software v 25.0. Data were presented in terms of regression coefficients and/or odds ratio, and statistical significance was set at *p* < .05.

**Results:** Both groups were comparable at baseline on demographic characteristics and outcome values. A statistically significant effect of the intervention was found in the mental health at both post-intervention (β = −6.40, 95% confidence interval [CI] [–8.89, –3.91], *p* < .001) and follow-up assessments (β = −7.14, 95% CI [–9.89, –4.39], *p* < .001). Similarly, significant improvements on quality of life were seen at both post-intervention (β = 2.98, 95% CI [2.13, 3.83], *p* < .001) and follow-up (β = 2.45, 95% CI [–1.51, 3.39], *p* < .001) among intervention participants compared with control group. Significant improvements were seen in the perceived social support and help-seeking behaviors among intervention attendees compared with those in the control group (*p* < .05).

**Conclusion:** To our knowledge, this is the first RCT to assess the effectiveness of a counseling intervention on mental health among pregnant women having a history of abuse in Nepal. Findings from this study support the feasibility and applicability of a brief counseling intervention to improve the emotional well-being of abused women in limited-resource settings.

## “We Don’t See Because We Don’t Ask”—Qualitative Exploration of Service Users’ and Health Professionals’ Views Regarding a Psychosocial Intervention Targeting Pregnant Women Experiencing Domestic and Family Violence

### Diksha Sapkota^1,2,3^, Kathleen Baird^1,4^, Amornrat Saito^1,3^, Pappu Rijal^5^, Rita Pokharel^5^, and Debra Anderson^1,3^

^1^Griffith University, Brisbane, Queensland, Australia

^2^Kathmandu University, Dhulikhel, Nepal

^3^Menzies Health Institute Queensland, Brisbane, Queensland, Australia

^4^Gold Coast University Hospital, Gold Coast, Queensland, Australia

^5^B.P. Koirala Institute of Health Sciences, Dharan, Nepal

**Introduction:** Emerging literature from developed countries supports the use of counseling and safety planning for addressing the emotional needs of women who have been the victims of domestic and family violence (DFV). Given the relative recency of DFV management as a field of endeavor, it is not surprising that interventions for addressing DFV is still in its infancy in developing countries. This paper describes the feasibility and efficacy of conducting a psychosocial intervention targeting pregnant women with a history of DFV in Nepal.

**Method:** A total of 63 pregnant women who participated in the intervention and seven health care providers (HCPs) involved in care of pregnant women were interviewed using semi-structured interviews. Thematic analysis was used to analyze the data. Final codes and themes were identified using an iterative review process among the research team.

**Results:** Three domains reflecting the research questions were identified: DFV and its response mechanisms, impact on women’s lives, and improving accessibility and sustainability of the program. DFV was recognized by all HCPs as a significant problem requiring urgent attention for its prevention and control. Intervention participants expressed the counseling session as a safe haven that allowed them to share their feelings while learning new skills to cope with DFV. The majority of the participants recommended multiple sessions during the counseling and a continued provision of service to ensure its accessibility by a large number of women.

**Discussion:** This is the first study to document the feasibility of conducting an antenatal-based intervention for addressing the negative consequences of DFV in Nepal. There was a clear consensus around the need to engage, support, and empower victims of abuse and the intervention was well received by the participants. Ensuring good mental health and well-being among victims of abuse requires work across individual, organizational, and community levels.

## What Is Non-State Torture of Women and the Girl Child? What Is NST Victimization–Traumatization Informed Care?

### Jeanne Sarson^1^ and Linda MacDonald^1^

^1^Persons Against Non-State Torture

**The Problem:** Non-State torture (NST) victimization is rarely named as a form of violence inflicted against women and the girl child. We promote its visibilization and explain its global occurrence.

**The Significance:** Although NST is a human right violation, its significance in practice has remained globally invisibilized; thus, NST victimization–traumatization informed care has not developed. In law countries may take the stance that UN recommendations for its inclusion in country law are “soft law” recommendations thus ignored further delaying sociopolitical contribution to the healing process.

**Purpose:** We began our NST support (https://everywoman.org/) of women in 1993 and have never left this focus. The term non-State torture originates from “non-state actors” when Amnesty International published its booklet in 2000 addressing State responsibility for abuses perpetrated against women by non-state actors. Although the first women who disclosed to us was Canadian, born into a NST human trafficking family system, women in the United States, the United Kingdom, Western Europe, Australia, and New Zealand also contact us, as do counselors supporting woman who have survived NST. Our prime focus has been women who were born into NST human trafficking family systems which generally have like-minded connections to other individuals or groups. In 1993, there was no written literature drawn on for the delivery of care; therefore, we will share the innovative practice we developed that explains what NST is and discuss our model of NST victimization–traumatization informed care which addresses lessons learned, the implications that NST integrative recovery demands, and share educational information into the global categories of NST. Included are results of our NST participatory qualitative research including on conditioned suicidal-femicide. We would appreciate placing visual models in a PowerPoint presentation to also share women’s drawings they used to “show” the NST human trafficked victimizations they suffered. Overcoming this human rights discrimination in law and practice, we advocate supporting the global initiative for the legally binding treaty on violence against women and girls of Every Woman Treaty (http://nonstatetorture.org/).

## Expanding the Role of the Registered Nurse to Address the Health Effects of Violence and Injury in Rural Older Adults Using U.S. Medicare Annual Wellness Visits

### Erika Metzler Sawin^1^, Donna Schminkey^1^, Sandra Annan^1^, and Deborah Elkins^1^

^1^James Madison University, Harrisonburg, Virginia, USA

**Describe the Problem or Issue Addressed:** In this pilot study, registered nurses (RNs) who conduct Medicare Annual Wellness Visits (AWVs) in the United States for adults age 65 and older received focused education on power-based personal violence (PBPV), unintentional injury (UI), and trauma- and violence-informed care (TVIC). AWVs are used as a screening, intervention, and referral vehicle. This comprehensive visit includes medical and family history review, physical assessment, functional abilities, home safety, biometrics, and screenings for substance use, depression, and cognitive impairment. Screening for and management of PBPV and/or UI and their sequelae are not specifically addressed in routine preventive care and disease management of older adults.

**Significance:** UI and PBPV rates are higher in rural areas. Older adults often do not disclose PBPV. Nurses working in an expanded role in the primary care setting are able to incorporate assessment data into their chronic care management of these patients. These interventions allow nurses to identify people who would benefit from ongoing care management and rehabilitation services.

**Purpose of the Session:** Rural primary care RNs were interviewed regarding their experiences and abilities to identify, intervene, and refer people who have experienced often previously undisclosed PBPV and/or UI. Findings and recommendations from this pilot study will be discussed, including the ability to integrate assessment and interventions.

**Approach or Innovation:** The researchers performed qualitative interviews of RNs who work in federally designated primary care rural health clinics. Questions included ways to identify specific strategies for intervention and patterns of health sequelae that stem from injury, and how nurses can leverage the AWV to address PBPV and UI.

**Lessons:** RNs who have received specific education on PBPV, UI, and TVIC can individualize visits as they identify and address these issues in older adults using education and referrals.

**Implications:** Nurses can address PBPV, UI, and their sequelae in older adults in the primary care setting, through tailoring the existing AWV structure using principles of TVIC. Chronic illness frameworks and related nursing roles (e.g., chronic care management, preventive care, health coaching, and transitional care management) are useful to improve nursing’s response to PBPV and related issues.

## Testing the Relationships Between Intimate Partner Violence, Mastery, Social Support, and Mental Health in Canadian Women

### Alice Pearl Sedziafa^1^, Marilyn Ford-Gilboe^1^, Kelly Scott-Storey^2^, and Michael Kerr^1^

^1^Western University, London, Ontario, Canada

^2^University of New Brunswick, Fredericton, New Brunswick, Canada

Extensive cross-sectional studies suggest personal and social resources mitigate the adverse consequences of intimate partner violence (IPV). But the literature is specifically limited in longitudinal research on the causal mechanisms linking IPV to mental health. Addressing this limitation will advance our theoretical understanding of the mechanisms shaping mental health in women who have experienced IPV. The proposed research will examine the relationship between IPV, social support, mastery, and mental health in women in varying social contexts. A multivariate structural equation technique will be used to analyze secondary longitudinal quantitative survey data collected from a sample of 462 women who have experienced IPV, recruited online through the iCAN plan 4 safety (https://icanplan4safety.ca/) trial. The proposed study has important implications. It will illuminate the personal strengths of women overcoming the effects of IPV, promote appreciation of the social support required by women with diverse social characteristics, and advance the theoretical understanding that perceptions of resources may be mutable over time and interventions can heighten these perceptions to improve mental health.

## Institutional Challenges to Delivering Domestic Violence Services in Ghana: A Case of Structural Violence?

### Alice Pearl Sedziafa^1^, Eric Y. Tenkorang^2^, and Emmanuel Banchani^2^

^1^Western University, London, Ontario, Canada

^2^Memorial University of Newfoundland, St. John’s, Newfoundland, Canada

**Background:** To eradicate or reduce domestic violence, it is important to understand the processes of help-seeking among those who experience interpersonal violence. Advocates for holistic channels for addressing domestic violence argue this is a matter of structural justice. In Ghana, the Domestic Violence and Victim Support Unit (DOVVSU) was established in 1998 as a specialized unit of the Ghana Police Service. As a national institution, DOVVSU is tasked with providing legal assistance to people who experience domestic violence. However, inequitable social, cultural, economic, and legal challenges limit the power of DOVVSU to deliver equitable and dignifying assistance to its clients and hold perpetrators of violence accountable. In this paper, we document institutional challenges to delivering domestic violence services in Ghana.

**Method:** Qualitative in-depth interviews were collected from 30 female survivors of intimate partner violence (IPV) and 15 staff at DOVVSU in the Greater Accra, Ashanti, and Northern regions during fieldwork conducted from May to August 2019. The regions were randomly selected to represent the three main ecological zones (Coastal, Middle, and Northern) in the country. Data were analyzed using the qualitative software NVivo. The data were subjected to thematic analysis where codes were generated and themes developed based on the codes.

**Results:** Female survivors of IPV rarely accessed services at DOVVSU as majority claimed they had not heard about them. However, those who knew DOVVSU and had accessed their services mentioned fear, loss of rights to privacy, and financial problems as major barriers to accessing their services. From an institutional perspective, staff at DOVVSU identified noise pollution, lack of privacy for clients, inadequate logistics, and limited training for DOVVSU staff as among various reasons for their ineffective delivery of services to victims of violence.

**Conclusion:** Policy responses to dealing with access to domestic violence services should move beyond individual-level approaches, to consider structural factors that militate against delivering effective service to survivors of IPV.

## Stakeholders Roles and Views of Care of Women With FGM Experiencing the Postpartum Period

### Rebecca Seymour^1^, Elizabeth Bailey^1^, Katherine Brown^1^, and Hazel Barrett^1^

^1^Coventry University, Coventry, United Kingdom

**Objective:** To critically examine the roles and views of stakeholders who affect care during the postpartum period for women with female genital mutilation (FGM).

**Background:** The eradication of FGM is supported by the World Health Organization (WHO), UNICEF, and the UK government. The WHO estimates approximately 200 million women are living with FGM today, with 3 million at risk of having the procedure completed annually. Maternity services are often the first point of contact women with FGM have with the UK health services. While previous research has focused on antenatal care, there remains a lack of research focusing on postpartum care.

**Design:** A focused ethnography was conducted using a cultural context and a feminist lens. Semi-structured interviews took place with stakeholders who were directly and indirectly responsible for providing care during the postpartum period for women with FGM. Interviews were transcribed verbatim and analyzed using thematic analysis. Stakeholders were contacted if follow-up questions arose.

**Findings:** Stakeholders were divided into two categories, impact through direct care and impact through policy. Preliminary analysis shows policy stakeholders report positive care impacts, meeting the needs of the community, and positive feelings about their job. Direct care stakeholders report negative care impacts, not meeting the needs of the community, and viewing their job as a vocation that occasionally has negative impacts on their mental health. A tension was evident between the two groups.

**Conclusion:** A tension exists between policy makers and direct care providers for women with FGM, specifically during the postpartum period; further research and communication is needed to understand this tension. The voices of women with FGM are needed to understand the experience of receiving care during the postpartum period. The implications for this study are broad and touch on policy development for post-partum guidelines (currently unavailable for women with FGM), education for health care providers and public health workers, and the development and distribution of resources dedicated to women with FGM. Continued research is needed in this area.

## Taking the Control Out of Birth Control: Reproductive Coercion and the Nurses Role

### Trisha Sheridan^1^, Jacqueline Callari Robinson^2^, and Jenanne Luse^1^

^1^Emory University, Atlanta, Georgia, USA

^2^University of Wisconsin–Milwaukee, Milwaukee, Wisconsin, USA

Reproductive coercion (RC) is a form of violence including birth control sabotage, pregnancy pressure, and control of pregnancy outcomes. As many as 15% to 25% of people seeking care at family planning clinics report experiencing RC, with adolescents and those experiencing intimate partner violence (IPV) reporting an even higher prevalence. While all health care providers play an important role in identifying, assessing, and responding to control of reproductive outcomes, nurses and those working with victims of violence are especially likely to encounter RC. Victims of RC are hesitant to come forward and self-identify as this form of IPV is often omitted from screening questionnaires or masked by more well-known physical or emotional violence symptoms. As a result, RC is often overlooked or neglected as a valid and impactful form of IPV. Taking this into consideration and in combination with significant under reporting, health care provider knowledge of and familiarity with this form of IPV is often limited, even by experienced nurses who are trained to recognize signs of IPV. Given the often silent nature of RC, its prevalence, and the lack of awareness among nurses, the need for increased educational opportunities is paramount. As patient advocates and clinical educators, nurses are uniquely poised to strengthen RC screening and response guidelines in the clinical setting, as well as to develop and promote educational tools for health care providers in a variety of primary and acute care settings. This session will allow participants to define and discuss RC including how it presents in a variety of clinical settings. The aim is for participants to understand their role in assessment, intervention, and referral for victims of reproductive coercion.

## Creating an Instrument to Better Measure Healing in Female Survivors of Gender-Based Violence

### Laura Sinko^1^, Andreea Beatrix Taran^2^, and Denise Saint Arnault^2^

^1^University of Pennsylvania, Philadelphia, Pennsylvania, USA

^2^University of Michigan, Ann Arbor, Michigan, USA

**Problem:** Current literature to date has primarily equated gender-based violence (GBV) recovery with an improvement of mental illness symptoms, causing a gap in our understanding of the impact of interventions beyond the amelioration of adverse symptomology.

**Purpose:** To create an instrument to holistically measure GBV recovery based on the healing goals and desires indicated by female survivors in the community.

**Study Design/Sample:** (a) Qualitative, ethnographic interviews were conducted to develop instrument items (*n* = 56), (b) focus groups of community experts were used to provide initial measure feedback (*n* = 8), and (c) cognitive interviews with GBV survivors in the community were used to provide additional feedback (*n* = 9).

**Data Collection Approach/Analysis:** Qualitative, ethnographic interviews were conducted in three separate samples of female GBV survivors (ages 18–76) recruited through a health system research portal as well as through email listservs and flyers targeting community GBV organizations. Healing themes were extracted from a larger grounded theory analysis based on these interviews, and items were developed based on healing subthemes using survivor language. Focus groups and cognitive interviews were then conducted with both experts and GBV survivors to revise the measure prior to psychometric testing.

**Results:** A 31-item instrument was created to measure both impact and healing progress on a 5-point Likert-type scale.

**Implications:** The Healing After Gender-Based Violence measure has the potential to highlight survivor strength and growth while more accurately measuring recovery based on survivor indicated goals and desires. This measure can also provide survivor insight on the progress they have made thus far and what their healing needs may be going forward.

## Violence Appraisals, Disclosure, and Service Utilization: How Culture and the Normalization of Violence Against Women Intersect in Irish and American Survivors of Gender-Based Violence

### Courtney Julia Burns^1^, Laura Sinko^2*^, and Denise Saint Arnault^1^

^1^University of Michigan, Ann Arbor, Michigan, USA

^2^University of Pennsylvania, Philadelphia, Pennsylvania, USA

*Presenting Author

**Purpose:** While literature alludes to the effect of normalization of violence against women (NOVAW) on perpetration of gender-based violence (GBV), little is known about how it affects survivor disclosure, service utilization, and violence appraisals. In addition, there is a need to understand how normalization manifests in different cultural spheres. Therefore, the purpose of this study was to examine how normalization manifests in both Irish and American societies and how this affects subsequent meaning-making in survivors of gender-based violence.

**Study Design:** The Clinical Ethnographic Narrative Interview was used to understand the meaning-making process after experiencing gender-based violence in 19 American and 12 Irish survivors. Cross-cultural comparison was achieved through the comparative ethnographic narrative analysis method.

**Results:** Among survivors in both samples, witnessing and experiencing repeated violence, as well as the responses of others upon disclosure, proved particularly influential in their ability to recognize violence normalization. Their resulting meaning-making processes were further influenced by negative self-appraisals, significant emotional burdens, and difficulty labeling violent experiences. Cultural nuances within each of these themes will be discussed.

## Innovations to Prevent or Respond to Gender-Based Violence

### Laura Sinko^1,2^

^1^University of Pennsylvania, Philadelphia, Pennsylvania, USA

^2^University of Michigan, Ann Arbor, Michigan, USA

As research on gender-based violence (GBV) grows in popularity, how do we ensure that we are translating this research appropriately and are continuing to innovate in this space? The purpose of this presentation is to discuss promising new innovations to dismantle violence normalization, situate culture in our conversation about perpetration and survivorship, and highlight the importance of expanding the science of recovery in future studies of GBV. Using research from our international GBV consortium MiStory, we will highlight current research translation efforts to bridge the gap between science and practice as well as current interventions under evaluation to promote healing in this population. We hope this will inspire attendees to consider non-traditional research dissemination in their practice as well as will highlight the potential of visual interventions in capturing sociocultural elements of recovery.

## Women’s Experience of Seeking Health and Social Services Following Intimate Partner Violence: Lesbian, Gay, Bisexual, Transgender, and Queer Relationships in Rural Communities

### Emily Soares^1^, Kim Jackson^1^, and Tara Mantler^1^

^1^Western University, London, Ontario, Canada

**Problem Statement:** Intimate partner violence (IPV) is a global public health issue and a violation of human rights. Although the body of literature around IPV continues to grow, there is a considerable gap in the literature focusing on lesbian, gay, bisexual, transgender, and queer (LGBTQ) individuals. Historically, IPV research has been largely based on heterosexual, cis-gendered women, with very little mention of those who do not identify as such. The exclusion of these women poses a barrier to understanding how best to support those experiencing IPV. Furthermore, the intersection of rurality creates additional challenges for a group that is already marginalized through the lack of understanding and access to resources.

**Purpose:** The purpose of this study is to explore the experience of LGBTQ-identifying women, living in rural communities, and with histories of IPV as they seek health and social services.

**Design and Sample:** This study is a secondary analysis of baseline data of the “Sharing Personal Experiences of Accessibility and Knowledge of Violence” (SPEAK) study. The SPEAK study included 20 rural women living in Southwestern Ontario who experienced IPV and had used health or social services within the past 6 months. The current study sample will be comprised of a subset of four women who are in an LGBTQ relationship.

**Data Collection Approach:** The study will use an illustrative, case study methodology for data abstraction through the use of focus groups and photovoice, a method of data abstraction that enables for the gain of enriched understanding of participant experience.

**Implications:** The study plans to illustrate both the barriers and facilitators present for LGBTQ women who experience IPV in a rural context and seek health and social services. The goal is to influence policy and practice as they pertain to these contexts.

## Establishing the One Stop Crisis Center in Numphong Hospital, Thailand

### Wichai Ussavaphark^1^, Somjit Somwang^1^, Patthayason Sukree^1^, and Tipparat Udmuangpia^2^

^1^Namphong Hospital, Khon Kaen, Thailand

^2^Boromarajonani College of Nursing, Khon Kaen, Thailand

**Background:** Domestic violence (DV) against women and children has been linked with physical, mental, and spiritual health of people in Thailand. The survivors in Thailand find it difficult to access services. Based on that problem, in 1999, the One Stop Crisis Center (OSCC) was established in Thailand to prevent and help DV survivors to connect with multi-professionals networking. At that time, the systems of helping and recording survivors were not prepared, and health care providers had not been trained in knowledge and skills. Therefore, Numphong hospital was established the OSCC at the hospital and started to develop the OSCC to be an inclusive center to prevent and help women affected by DV. The conceptual frameworks of developing the OSCC are holistic care, empower counseling, case manager, and community engagement.

**Method:** The process of developing the OSCC consisted of (a) establishing the multi-professionals advisory board to set the goals and strategic plans regarding prevention and provision to survivors, (b) implementing the plans, (c) improve the services at the OSCC by being available 24 hours with effective referral systems, (d) training the OSCC teams with multi-professionals training, and (e) making connections and resources with multi-professionals and meet together every month.

**Results:** The effectiveness of establishing this innovation has been measured by (a) having more women and children access services and facilities that OSCC provided, (b) creating several campaigns to stop DV in women and girls and having Marathon every year on International Domestic Violence Day (November 25), (c) providing the community health volunteers to observe and monitor the DV in communities that could establish a community model for preventing DV, (d) improving the skills and changing attitudes of health care providers to DV, and (e) creating the effective referral systems by integrating with communities and multi-professionals.

**Conclusion:** There are a lot of key success factors for establishing the OSCC in Numphong hospital, Thailand. One of them is people (leaders, providers, and communities) are aware of the DV problem in Thai society, and they want to change it. The concepts of establishing the OSCC should be disseminated to other settings and countries.

## Helping Women With Trauma Histories Require Specific Approaches in Help-Seeking: Introducing the Model of Help-Seeking Encounters

### Minna Sorsa^1^

^1^Tampere University and Pirkanmaa Hospital District, Tampere, Finland

Women are worldwide disproportionately affected by depression, anxiety, and such mental disorders that go unrecognized. Women’s experience of gender-based violence (GBV) has been found to relate to post-traumatic stress disorder (PTSD), depression, suicides, and substance abuse. It has been shown that women with a trauma history may not have words to express their needs, and thus specific woman-centered approaches within interventions are needed. There are elements in the women’s vulnerable background and experiential level that cause problems in connecting to new persons within care encounters. The aim of this presentation is to introduce the Model of Help-Seeking Encounters, which is directed toward meeting with women with a complex history of trauma as they seek help. The Model was developed in a multimethod study with the approach of help-seeking. The data were collected in Finland and the methods used include client interviews, an ethnographic field study, and interviews with staff within service encounters within primary care, social work, mental health care, and substance services. The opportunity for connections is created in the different interfaces where a micro-level of moments can prove valuable in the process of involvement. Engagement is the co-creation of possibilities between workplace staff and the client. It is not a single act, emotion, or verbal communication, but a complex intertwined system. The sensitivity of the worker is one tool for engaging the client in manifold ways: Even the smallest events are viewed as valuable. The work entails complexity in the negotiations over vulnerability. Engagement involves the intentional client in the process: The client needs to participate and become an acting and sensing part of the change, which occurs on an experiential level. The staff need sufficient time and resources to be available with an approach of perseverance. The goal of the meetings between clients and staff is to grow the grasp of life and the interfaces when clients can connect. The study questions whether current role expectations and operating within care structures are exclusive rather than inclusive by nature. Addressing the issue is important as half of women with GBV do not use formal services because they regard violence as normal.

## A Realist Analysis of Mechanisms Underpinning Intimate Partner Violence Screening and Decisions to Disclose Abuse in Antenatal Care

### Jo Spangaro^1^, Jeannette Walsh^2^, Kim Spurway^3^, Kelsey Hegarty^4^, and Jane Koziol-McLain^5^

^1^University of Wollongong, Wollongong, New South Wales, Australia

^2^University of New South Wales, Sydney, New South Wales, Australia

^3^Western Sydney University, Sydney, New South Wales, Australia

^4^The University of Melbourne and Royal Hospital for Women, Melbourne, Victoria, Australia

^5^Auckland University of Technology, Auckland, New Zealand

**Problem Statement:** Routine screening for IPV has been introduced in many antenatal settings globally; however, screening rates are often low, as are disclosure rates relative to known levels of current IPV among pregnant women.

**Purposes and/or Questions/Hypotheses:** What contextual factors and mechanisms underpin high rates of screening and disclosure of intimate partner violence in the context of antenatal care?

**Study Design:** We applied a case study approach employing realist analysis to explore mechanisms underpinning screening and disclosure rates and contributing contextual factors. We drew on three data sets comprising (a) clinic screening and disclosure rates, (b) patient interviews, and (c) focus groups with midwives and social workers at each site.

**Sample:** Three demographically diverse antenatal clinics in the state of New South Wales Australia were studied. Participants comprised antenatal patients who had experienced past 12 months IPV, as well as midwives and social workers at each site.

**Data Collection Approach:** Antenatal patients were recruited through health worker referral and flyers for participation in telephone interviews. Midwives and social workers were invited to participate in separate focus groups at each site. Past 12-month data on screening and disclosure rates were also examined for each site.

**Analysis of Results:** Procedurally driven approaches to screening, including policies to exclude partners from the first antenatal visit, were a mechanism which underpinned high screening. An alternate mechanism also evident was prioritizing woman-centered care, which seemed to result in lower initial screening, in response to deferring screening due to the presence of partners. A relationship was identified between this mechanism and higher disclosure rates, which was the second outcome of interest. A second mechanism for decisions to disclose abuse was “judging it safe to tell,” triggered in contexts where midwives appeared to engage wholistically and worked with a sense of having a team behind them.

**Implications:** Clear policies are required for effective implementation of IPV responses; however, the relational aspect of asking and responding must be emphasized not only to ensure high screening rates but also to support women to safely disclose abuse they experience.

## Non-Fatal Strangulation: What Do We Know and What Are We Missing to Prevent Sexual Violence?

### Amanda St Ivany^1^, Donna L. Schminkey^2^, Michelle L. Munro-Kramer^3^, Lindsay M. Cannon^3^, and Cindy Pierce^4^

^1^Dartmouth-Hitchcock Medical Center, Lebanon, New Hampshire, USA

^2^James Madison University, Harrisonburg, Virginia, USA

^3^University of Michigan, Ann Arbor, Michigan, USA

^4^Independent Author, Social Sexuality Educator and Speaker

**Description of the Problem to Be Addressed:** As nurses and midwives, we care for women who have experienced non-fatal strangulation (NFS) whether or not we screen or it is disclosed. As violence researchers, we assess lethality risks associated with strangulation, yet there is limited knowledge around accounting for and including NFS non-related to violence, or NFS as it doesn’t pertain to sexual violence, through the lens of future violence prevention. How do we measure progress in NFS prevention? Greater assessment of NFS means higher numbers recorded, improved medical interventions mean potentially decreased lethality, but what are we missing by not considering other mechanisms of NFS? It is necessary to begin to understand how the neurobiological changes that may occur after NFS (whether or not it is violence related) could make someone more prone to become a victim or perpetrator of violence in the future.

**Aims of the Symposium:** Provide overview of neurobiological changes after NFS, present trends in reported NFS from forensic nursing data, explore findings from interviews on increasing trends in NFS, and discussion with a community-based social sexuality educator describing the increase of NFS in consensual (and beyond boundaries of consent) sex.

**Symposium Format:** The symposium will be presented in four parts with approximately 10 minutes for each part (~40 minutes total for presented material). First, an overview of the neurobiology of repeating brain injuries/strangulation and why it is important to understand not just the relationship to lethality but also the ways people’s brains could be changing (specifically adolescent/young adult women). Second, NFS data will be presented from female patients receiving sexual assault nurse examiner (SANE) care in a hospital in the United States, including showing the increase in NFS over time and discussing changes in SANE questions assessing strangulation. Third, many states in the United States are making strangulation a felony, and with more police departments and first responders implementing the Danger Assessment or other forms of questions about strangulation, higher numbers of NFS are being recorded. Preliminary data from a grounded theory study will be presented to ask the questions “is strangulation really increasing, or is it how we talk about and measure it?” Finally, a brief overview of community-based work as social sexuality educator, with focus on what we are missing from a research perspective: porn, social media, hook up culture, and cultural messaging that have created the expectation that aggressive sex, including strangulation, is an expected option during sex, even at a young age of sexual debut.

**Focus of the Discussion** (~40 minutes): What can we do to prevent strangulation and sexual violence? Bringing it all together and opening up for audience discussion and participation: questions around what people are seeing around the world related to NFS, what are interventions to decrease NFS, and how can we build relationships between researchers and community-based organizations to begin to unpack the complex relationships between NFS and violence victimization and perpetration.

## Sexual Harassment in Tanzania—An Exploratory Study

### Heidi Stöckl^1^, Joyce Wamoyi^2^, and Meghna Ranganathan^2^

^1^London School of Hygiene & Tropical Medicine, London, United Kingdom

^2^National Institute for Medical Research, Mwanza, Tanzania

The ground-breaking anti-sexual assault and harassment movements, Time’s Up and #MeToo, elevated global awareness of the offending actions that women encounter in their daily personal and working lives. Their ambitious aims are crucial for meeting the world’s sustainable development goals. However, there is no clear understanding of women’s range of experiences of sexual harassment globally. The few studies conducted on sexual harassment come primarily from Europe and North America with little attention to harassment in developing countries. Our study offers a first step toward understanding how these abuses occur and how they are perceived by and affect women in the Global South. For this study, we have conducted 90 in-depth interviews and nine focus group discussions with women and men, girls and boys, and policy makers in Mwanza, Tanzania. Interviews were conducted on sexual harassment in educational settings, public spaces, and in the workplace. The findings of our study suggest that sexual harassment is a normative occurrence across the discourse in the different settings, with both men and women and boys and girls being able to relate to experiences of it. For example, in educational settings, stories of sexual harassment focused on sexual comments by teachers toward female students. These were both in the classroom and in other places at the schools and indicated existing pressures of entering into sexual relationships. What became apparent in all the interviews was that sexual harassment was not only confined to the three settings investigated but went beyond, including sexual harassment by family members and neighbors. Women related to several strategies they engaged in to prevent and reduce sexual harassment that showed the impact it had on their daily activities, as well as their emotional well-being. Sexual harassment remains a concept that still warrants a clear definition to make it measurable across different settings. From the narratives obtained in our study, we know that it is highly prevalent in Mwanza, Tanzania, and that acknowledging and preventing it can have a positive impact on women’s lives.

## The Multiple Facets of Violence—A Qualitative Study From Tanzania

### Heidi Stöckl^1^, Joyce Wamoyi^2^, and Meghan Ranganathan^2^

^1^London School of Hygiene & Tropical Medicine, London, United Kingdom

^2^National Institute for Medical Research, Mwanza, Tanzania

Intimate partner violence is still often only perceived to be physical or sexual, with a fair level of international agreement on its definition and measurement. While emotional abuse and controlling behavior have received increased attention, there is a lack of clarity on what acts constitute it and whether women perceive them as abusive. Even with sexual violence, in cultures where husbands are believed to have a right to have sex, there is a lack of clarity what constitutes sexual violence. Our study aimed to address this gap by interviewing 18 women in depth about their relationship and their experiences and perceptions about intimate partner violence. The 18 women were purposively sampled based on their participation in the MAISHA longitudinal study and because they have indicated a change in their experience of sexual intimate partner violence between the first two rounds of their quantitative surveys. Interviewers specifically probed about what kind of behavior women found acceptable in their relationship, what kind of behavior made them uncomfortable, and what they clearly considered violence and abuse. While specific acts were queried, the focus of the interviews was to encourage women to speak about the experiences of violence and abuse that were most important and pressing for them. Women spoke about a multiple set of violence experiences from their partner, with a strong focus on economic abuse that were linked to economic hardships they and their families suffered. Beyond acts that are commonly captured under economic abuse, such as taking earnings and not allowing a woman to make decisions over her own income, women also perceived the inability of men to provide for the household as a form of abuse. Regarding sexual violence, women also raised being asked about anal and oral sex, sex taking too long, or being denied sexual pleasure as experiences they would consider violence and abuse. The findings of this study showed that women consider a broad range of experiences as violence and that they have a wider sense of what is abusive than what is currently captured in existing screening tools that offer a range of acts considered to be violence but which are not necessarily holistic and consider the context sufficiently.

## The Feminism Welcoming Women With Disability in Women’s Shelters and the First Italian Observatory on Multiple Discrimination

### Rosalba Taddeini^1^

^1^Differenza Donna NGO, Roma, Italy

Our paper will introduce the innovative practice to welcome and host women with disabilities (WWD) victims of violence at the Women’s Shelters and the actions we have designed, the obstacles we found and overcame, in order that it could be a valuable contribution and a moment of reflection for all the women of the Nursing Network on Violence Against Women International. Differenza Donna has been the first nongovernmental organization (NGO) in Italy overcoming that gap which had never included WWD into feminism, creating and launching a National Observatory on Gender-Based Violence on WWD. Through this Observatory, we report inefficient services, unable to decode situations of violence and to intervene in public policy, which too often does not take into account such discrimination, not including, for example, indicators capable of bringing to light the gender and disability perspectives in an organic way. The way in which gender-based violence is perpetrated on women with disabilities is similar to that on women without disabilities, but it starts from different roots. The assumption is that the former are not represented as a sexual object by patriarchal culture but they are seen as eternal girls or angels, unlike women with cognitive/intellectual disabilities who are perceived as hypersexualized in the collective imagination. This topic also emerged from a research we conducted with the University of Kent in 2016. Thanks to Focus Groups with WWD on issues of women’s rights, on sexuality, and gender-based violence, it turned out that 97% of them had suffered violence and sexual abuse at least once in their lifetime. We believe that the subject of the inaccessibility of Shelters by WWD is not tied exclusively to the physical place, but it starts from a culture of body objectification, which women without disabilities always have fought, not including WWD. The awareness of the violence suffered and of access to Shelters is not a foregone conclusion to WWD. Since 2014, we have met 120 WWD and we found different strategies to respond to the needs of women who overcome violence: We provide nursing assistance service inside the Shelter for WWD; we work as experts called by the Criminal Court to ensure the reliability of the violence reported by WWD.

## Shedding Light on the Role of Gender Attitudes and Alcohol Abuse in Preventing and Reducing the Cycle of Intimate Partner Violence: A Multi-Country Analysis of Male Perpetration

### Angela Taft^1^, Anne-Marie Laslett^1^, Sandra Kuntsche^1^, Emma Fulu^2^, Ingrid Wilson^1,3,4^, and Kathryn Graham^5,6,7,8,9^

^1^La Trobe University, Melbourne, Victoria, Australia

^2^The Equality Institute, Melbourne, Victoria, Australia

^3^Singapore Institute of Technology, Singapore

^4^University of Liverpool in Singapore, Singapore

^5^Centre for Addiction and Mental Health, Toronto, Ontario, Canada

^6^Dalla Lana School of Public Health, Toronto, Ontario, Canada

^7^Deakin University, Melbourne, Victoria, Australia

^8^Curtin University, Perth, Western Australia, Australia

^9^Western University, London, Ontario, Canada

**Problem Statement:** Although the exact role of alcohol in violence against women is complex and contested, there is clear evidence that harmful alcohol use is associated with frequency and severity of violence against women, and that the alcohol and violence-related behaviors are significantly gendered. However, while our previous studies have shed light on the lived experience of women of alcohol-related partner violence, we have also illustrated that there is little evidence from either violence or alcohol research about effective strategies for preventing or reducing alcohol-related intimate partner violence.

**Purposes:** This presentation will discuss results of several studies we have conducted to provide a stronger evidence base on which interventions to reduce alcohol-related partner violence can be designed. We will highlight results from our current analysis of the United National Multi-Country Study of Men and Violence to assess the relationship of lower gender equitable attitudes and heavy episodic drinking (HED) with the perpetration of intimate partner violence.

**Study Designs and Sample:** We analyzed data from 8,083 married or ever-partnered male respondents in six countries (Bangladesh, Cambodia, China, Indonesia, Papua New Guinea [PNG], and Sri Lanka) who participated in the United Nations Multi-Country Study on Men and Violence.

**Data Collection and Analysis:** We categorized the major exposure variable of alcohol use as heavy episodic drinkers, drinkers but not more than 6+ monthly, and abstainers. Physical or sexual intimate partner violence (IPV) in the previous year was the outcome. We also included the Gender Equality Score: low (1) versus moderate or high equality (0) as an independent variable. We used logistic regression for each country and the combined sample.

**Results:** We found a significant relationship with perpetration of partner abuse for both gender inequitable attitudes (adjusted odds ratio [OR] = 1.50, confidence interval [CI] [1.27, 1.78]) and HED (adjusted OR = 3.05, CI [2.48, 3.75]), but no significant interaction. We will present results of further analyses currently being undertaken.

**Implications:** We argue an urgent need to bridge the gap between scholars of alcohol and partner abuse, to highlight the role of gender in both fields, to develop and test effective interventions to reduce alcohol-related partner violence.

## An Interpretive Descriptive Study About the Mental Health Impact of Cumulative Lifetime Violence in Men: Interviews From a Sample in the Men’s Violence, Gender, and Health Study, New Brunswick, Canada

### Petrea Taylor^1^, Sue O’Donnell^1^, Judith Wuest^1^, Jeannie Malcolm^1^, and Charlene Vincent^1^

^1^University of New Brunswick, Fredericton, New Brunswick, Canada

Men experience mental health (MH) problems differently than women. Men are three times more likely to die by suicide and are less likely to seek help for their MH; however, gender is often neglected within MH research and service delivery. While a biomedical approach has not accounted for these differences, social determinants of health (SDOHs), including gender, are important in the understanding of men’s MH. Another SDOH, violence, intersects with gender and contributes to poor MH; however, its impact is misunderstood within research. Violence is misrepresented as associating with health based on singular “types” of violence that occur in isolation, failing to capture the effect of cumulative lifetime violence (CLV). For these reasons, we sought to understand how gender and having experienced CLV as a victim and/or perpetrator affect men’s MH. We interviewed 32 men from a larger sample of 586 men in New Brunswick, Canada, for The Men’s Violence, Gender, and Health Study (MVGHS) in 2016–2017. Interview transcripts were analyzed using an interpretive descriptive approach with a grounded theory lens. Data were coded for themes and categories, while relationships were established between the concepts. Meaning was derived from men’s descriptions using an interpretative process situated in a gendered perspective of “what it means to be a man” and the ongoing effects of CLV on MH. Findings revealed that the crux of men’s MH problems were realized through their relationships. Relational Distortion emerged as the basic social problem and is managed by Rectifying My Stance With Others, the basic social process. Rectifying occurs while Disengaging and Inserting My Will, subprocesses that are guided by differing levels of personal and relational understanding. Trauma- and violence-informed practices that draw awareness of gender role expectations and relational power dynamics will support capacity to manage their difficulties with day-to-day functioning in men with CLV histories.

## Disparities in Health Care Experiences and Outcomes for African American Survivors of Violence

### Elizabeth Tomlinson^1^

^1^North Carolina Central University, Durham, North Carolina, USA

**Problem Statement:** Disparities in health care and health outcomes experienced by communities of color and by survivors of violence are well-documented in research. However, the “cumulative burden of lifetime adversities” on women of color who have survived violence has been under-investigated.

**Purpose:** This study seeks to address this gap by reporting on a sample of trauma-exposed African American women:

- Whether and to what extent they show elevated inflammatory biomarkers (i.e., C-reactive protein; interleukin-6);
- Their trauma exposure, psychological symptoms, and discriminatory experiences;
- Descriptions of health care discrimination they have experienced; and
- Strategies they use to navigate difficult and/or discriminatory health care experiences.

**Design:** This mixed-methods pilot study (*N* = 60) uses bioanalysis, survey methods, and focus group discussions. Bioanalysis is used to measure the physiological consequences of stress for participants, and survey analysis is used to assess trauma exposure, psychological well-being, and discrimination. Analysis of focus group transcripts provides narrative context for experiences of trauma and discrimination and illustrates connections between trauma, discrimination, and health care experiences.

**Results and Implications:** Data collection is ongoing. We anticipate that findings from this study will provide continued evidence for the disparities in health outcomes experienced by African American women and survivors of violence; more data on the cumulative nature of these health disparities; and novel data on connections between health outcomes and health care experiences, and health care strategies for this vulnerable population of trauma survivors.

## Female Genital Mutilation Survivors in Media—Pictures That Empower

### Elisabeth Ubbe^1^

^1^eubbe press, Stockholm, Sweden

**Description:** In 2017, Swedish work on female genital mutilation (FGM) accelerated with the Amel project, which raises awareness and knowledge about FGM and educates thousands of employees in health care and various authorities. Photographer and registered nurse midwife Elisabeth Ubbe, whose work focuses on pictures of women, collaborated with the Amel project to collect images and narratives to highlight the situation for girls and women who live with the consequences of FGM in Sweden. In the seminar, Elisabeth Ubbe will discuss how images of FGM survivors in media often look and how more empowering images could be a change factor. In addition, she will highlight how prejudices risks preventing equal health care.

**The topic:** A total of 200 million females are estimated to have undergone FGM and 3 million girls are at risk, every year. Growing migration has increased the number of FGM survivors outside their country of origin, and they need to be included as equal citizens. With pictures that are empowering and making space for these girls and women in media, the seminar provides examples of how we can promote women’s agenda and empower our body rights.

**Description:** Elisabeth Ubbe highlights how media can be an active part in stopping FGM by using alternative pictures and telling other stories than the common, where FGM survivors often are presented as victims. Through the women’s experiences and stories Ubbe shares, the seminar will raise knowledge and awareness as well as create an impulse to act: to strengthen women, professionals, organizations, and nongovernmental organizations (NGOs) to work to change the structures in societies where FGM is practiced as well as to influence governments to criminalize FGM in all countries. Elisabeth Ubbe’s work highlights how images of FGM survivors as well as the common image of women in media maintain unequal structures and also shows examples of the opposite. Along with the seminar can be added a photo exhibition accompanied with an audiostory. An exhibition that has been shown both at the UN Women’s Conference in New York (2018) and at Vassar College, as well as in several places in Sweden.

## Using a Delphi Method for Adapting the Contents of the Women Abuse Screening Tool for Thai Women

### Tipparat Udmuangpia^1,2^, Supawadee Thaewpia^1^, Wacharee Amornrojawutthi^1^, Prapatsri Shawong^1^, Yaowaret Kamanat^3^, Pilin Nisatea^4^, and Tina Bloom^2^

^1^Boromarajonani College of Nursing, Khon Kaen, Thailand

^2^University of Missouri, Columbia, Missouri, USA

^3^Khon Kaen Hospital, Khon Kaen, Thailand

^4^Sakon Nakhon Hospital, Sakon Nakhon, Thailand

**Background:** The Woman Abuse Screening Tool (WAST) with eight questions is a screening tool for intimate partner violence (IPV) for women, which is widely used, well-validated, and used in clinical settings. Neither this tool has a Thai-language version nor has it been used among the Thai population before. Therefore, the purpose of this study is to translate and develop its contents to be culturally appropriate before examining psychometric properties.

**Method:** A three-round Delphi method was undertaken with Thai IPV experts. The WAST was translated and back-translated by three bilingual IPV experts to ensure the language was appropriate with Thai culture. The experts recruited were volunteers who work with abused women at least 2 years. After they agreed to participate in this study, the online survey was sent to them by social media (Line app, email, and Facebook). Responding of experts in each round was anonymous. Each question demonstrating ≥70% agreement on a 4-point Likert-type scale was determined to have reached consensus. Descriptive statistics were used by measuring of mean and standard deviation. Qualitative feedback from experts in each round was also analyzed and discussed.

**Results:** A total of 27 experts responded in this study and most of them are nurses (*n* = 11) and social workers (*n* = 8). No more questions have been added. All eight questions got average score at least 3.5. Experts suggested adding more behaviors of perpetrators that would be appropriate to Thai cultures. However, a question related to sexual violence has been suggested that it is important but it is sensitive to ask. Before asking this question, provider has to make sure that the woman could accept and not be offended.

**Conclusion:** This study is a first study to develop the screening tool for IPV in Thailand. Next step is doing the psychometric properties of this tool. The effectiveness of the WAST in Thai version will be examined then.

## A Mixed-Methods Research Predicting Intentions and Perceptions About Intimate Partner Violence Screening Among Nursing Students and Educators in Thailand

### Tipparat Udmuangpia^1^ and Tina Bloom^2^

^1^Boromarajonani College of Nursing, Khon Kaen, Thailand

^2^University of Missouri, Columbia, Missouri, USA

**Background:** The aims of this study were to examine how Thai nursing students’ beliefs (attitudes, subjective norms, and perceived behavioral controls) are associated with intention of intimate partner violence (IPV) screening, and explore perceptions of IPV screening among nursing students and nurse educators in Thailand.

**Method:** This study was conducted as a mixed-methods study, with primary data collection involving online surveys, focus groups, and individual interviews. Purposive sampling was employed. Inclusion criteria included nursing students who were in the fourth year of their nursing bachelor’s degree; nurse educators who have experience in education of at least 10 years. This study’s instruments were based on Theory of Planned Behavior. Logistic regression and mediation were utilized. Content analysis was used for qualitative method.

**Results:** The results from 639 online surveys and six focus groups with nursing students, and nine in-depth interviews with nurse educators showed that 88% reported intention to screen. The attitude and subjective norm are mediators of providing a screening tool at clinical site. Attitude and subjective norm significantly predicted intention by 31% of variance, but perceived behavioral control did not. Participants perceived that IPV screening is required, but it is difficult to screen because of the cultural consideration and not confidence. Participants do not feel well-prepared and confident by school in terms of knowledge and training experience.

**Conclusion:** This study is the first study to specifically explore the perceptions of IPV screening in nursing education in Thailand. The findings contribute to improving the nursing curriculum regarding IPV. More research related to prepare nursing students to deal with IPV issue is required.

## Implementing Trauma- and Violence-Informed Care in Diverse Contexts

### Colleen Varcoe^1^, Victoria (Vicky) Bungay^1^, and Nadine Wathen^2^

^1^University of British Columbia, Vancouver, British Columbia, Canada

^2^University of Western Ontario, London, Ontario, Canada

Trauma- and violence-informed care (TVIC), a universal approach, aims to provide safe care mindful of the circumstances of peoples’ lives. Rather than “screening” for violence, TVIC assumes that anyone coming to services may be experiencing, or have experienced, violence. Our approach is relational and multilevel: Intra-personally, providers need to consider how their own experiences of privilege and oppression shape their biases and assumptions; interpersonally, we need to be mindful of how each individual (providers and recipients) influences the other; contextually, we all operate in diverse policy, historical, and economic environments. TVIC requires significant shifts away from individualist approaches. This symposium invites participants to consider how to achieve such shifts. We open with an overview of TVIC as an equity-oriented concept, and consider how tools we’ve developed might be useful or adapted to participants’ own contexts (opening activity). Three papers then detail implementation of TVIC approaches in diverse contexts. We share the change process in which we are engaged in Emergency Departments, and in a woman-led model of outreach with women experiencing systemic inequities and severe interpersonal violence. Finally, presenting findings from an evaluation of TVIC workshops, we examine whether the TVIC approach is resonating with diverse service providers and leaders. Participants will be asked to consider how they might take up this equity-oriented approach, and how they could contribute to local and global TVIC efforts (closing activity).

Paper 1: TVIC in Emergency Departments (EDs).

*Colleen Varcoe, Annette Browne, Vicky Bungay, Nadine Wathen, Erin Wilson*

EQUIP is an evidence-informed, complex intervention integrating equity-oriented health care (EOHC) in diverse service contexts. Based on promising outcomes in primary health care, we have refined and are testing the intervention in EDs. A key dimension of EOHC is TVIC. Given the relationships among racism, colonialism, violence, and trauma in Canada, particularly against Indigenous people, a second key dimension is Cultural Safety; reflecting the relationship between these and substance use, harm reduction is the third dimension. We share our approach and preliminary results integrating TVIC in EDs, including use of “equity readiness” and “Front Line Ownership.”

Paper 2: Women-Led or Women Centered? Re-Imagining TVIC in an Outreach Context

*Vicky Bungay, Linda Dewar, Adrian Guta*

This paper considers “outreach” as a strategy to engage with women affected by violence, especially those regularly missing from services/programs. Women’s “absence” is associated with myriad circumstances including experiences of discrimination in care settings, siloed and disconnected services, competing demands necessary for survival (e.g., income generation, securing shelter), control by violent partners, and knowledge gaps concerning service navigation. TVIC approaches to outreach are critical to building trust and establishing sustained relationships that enhance women’s capacity to engage with and receive life-sustaining services. We share our TVIC approach fostering a women-led outreach initiative illustrating TVIC implementation in this context, and the associated benefits for women’s agency and engagement with services.

Paper 3: Uptake and Impact of TVIC Education in Diverse Service Contexts

*Nadine Wathen, Jennifer MacGregor, Sandy Beyrem, Western University*

We’ve delivered TVIC educational workshops in diverse contexts, including public health, primary care, and domestic violence services, since 2016. Pre–post evaluations indicate knowledge changes; however, uptake into practice remains unknown. We will report on follow-up evaluation of workshop impact. To date, 47 participants from 12 different sessions have responded to an online survey asking about impacts on their own practice, and on their organization. Based on responses, eight to 12 participants are being interviewed to explore these impacts, or lack of impacts. We will report on all data available at the time of presentation, to begin answering the question: Does TVIC education make a difference?

## Hiding in Plain Sight: A Discourse Analysis of Registered Nurses Capacity to Care for Female Intimate Partner Violence Presentations to the Emergency Department

### Vijeta Venkataraman^1,2^, Jane Currie^3^, and Trudy Rudge^3^

^1^Women’s Health Service, Canberra, Australian Capital Territory, Australia

^2^Canberra Sexual Health Canberra, Australian Capital Territory, Australia

^3^University of Sydney, Sydney, New South Wales, Australia

**Introduction:** The incidence of intimate partner violence (IPV) in Australia is rising. Women experiencing IPV seek assistance through Emergency Departments (ED) and are more likely to be exhibiting help-seeking behaviors toward nursing staff than toward other health professionals. The response of nurses is particularly important as ED nurses spend more time, and have greater opportunity to build trust and rapport with their patients when compared with their medical and allied health colleagues. Therefore, ED nurses’ capacity to recognize the need to care for women experiencing IPV is essential.

**Aim:** To explore nurses’ capacity to care for female victims of IPV through outlining the underlying inhibiting factors that limit nurses’ capacity to care, and create a discourse that may contribute to addressing these factors.

**Method:** Pre (*n* = 10) and post (*n* = 6) focus groups were undertaken with ED nurses studying a postgraduate qualification in ED nursing at a metropolitan University. In between the two focus groups, an intervention was applied to prompt change to caring practices. The discourse generated from the focus groups was subjected to a Foucauldian discourse analysis from a poststructural feminist perspective.

**Results:** Participants’ capacity to care for female victims of IPV was found to be based on the values they formed of IPV, as shaped by their training. The formation of boundaries was fundamental in inhibiting the participant nurses’ capacity to care for female victims of IPV.

**Conclusion:** Findings suggest that challenging of boundaries through educational inquiry into nursing values can be effective in achieving shifts in perspective of IPV. The raising of awareness of IPV in our communities will serve as an important and vital tool in eliciting cultural behavior change within our EDs and within our nursing culture.

## “We Need to Reach Them”: Child and Family Health Nurses Working With Women Experiencing Intimate Partner Violence

### Jeannette Walsh^1^ and Jo Spangaro^2^

^1^University of New South Wales, Sydney, New South Wales, Australia

^2^University of Wollongong, Wollongong, New South Wales, Australia

**Problem Statement:** Child and Family Health nursing services provide for all babies and parents in the state of New South Wales, Australia, with every family offered a home visit within 2 weeks of birth. How does identification of intimate partner violence (IPV) antenatally shape women’s access to Child and Family Health Nursing services and affect nurses’ safety?

**Purpose:** To understand Child and Family Health nurses’ approaches, understanding, and attitudes to providing services to women experiencing IPV.

**Study Design:** This cross-sectional online survey of Child and Family Health nurses was the second component of a mixed-methods explanatory sequential study. The survey aimed to develop an understanding from nurses working with women experiencing IPV: experiences, perceived facilitators, and barriers; safety; understanding and attitudes toward IPV; and professional supports.

**Sample:** A purposive sample of Child and Family Health nurses providing universal Child and Family Health services in three metropolitan health districts in Sydney, Australia.

**Data Collection Approach:** Nurses were recruited over a 4-week period via email from managers, with a 54% response rate. The survey was developed in consultation with Child and Family Health nurses, experts in IPV and child protection, with subsequent testing and piloting. Quantitative analysis provided descriptive analysis and bivariate and multivariate comparisons for perceived self-efficacy and fear of offending clients, with thematic analysis of free field responses.

**Analysis of Results:** Screening and responding to IPV were well accepted with participants showing high-level understanding of violence against women, high perceived self-efficacy, and low fear of offending clients. One quarter of participants had experienced IPV. Working alongside experienced colleagues and peer discussion assisted them to feel more effective. The majority of nurses agreed with occupational risk decisions, a minority questioned whether decisions were adequate or based on women’s current situation. IPV was not the only aspect of home visiting, which made participants feel unsafe.

**Implications:** These results highlight that Child and Family Health nurses are well placed to engage with women experiencing IPV; however, greater clarity about how IPV creates occupational risk is needed, given the likely impact IPV has on access to these services. This study has potential to inform policy for further development of risk assessment in the context of IPV.

## Gender-Based Violence: Myths, Misrepresentations, and What to Do About Them

### C. Nadine Wathen^1,2^, Eugenia Canas^1^, and Najibullah Naeemzadah^1^

^1^Western University, London, Ontario, Canada

^2^Centre for Research & Education on Violence Against Women & Children, London, Ontario, Canada

Gender-based violence (GBV) is a “wicked” social problem: prevalent, costly, harmful, and defying easy solution. Its causes are as much, or more, about what we believe and tolerate as a society, as they are about individual actions. We argue that two types of problematic beliefs about GBV exist. The first are myths and misunderstandings—beliefs based on outdated or mis-information, and/or ignorance of the scope and impact of GBV. The second stem from intentional messages to devalue and demean women and gender diverse people and are a feature of so-called men’s rights advocacy. We describe two inter-related studies designed to start shifting our shared narratives. News media play a significant role in the public’s understanding of GBV, with framing impacting support for funding, safe houses, legal sanctions for perpetrators, protection for victims, and so on. A gendered understanding of how the news media frames these issues is crucial. In Project 1, and with federal government partners, we use a nationally representative sample of online Canadian newspapers published over the last 30 years to examine one form of GBV: intimate partner violence (IPV). We conduct (a) a qualitative inductive framing analysis to capture emerging framings of IPV and (b) a quantitative deductive framing analysis to compare the differences in news media’s portrayal of IPV across genders. Project 2 describes how the results of Project 1, and related sources or knowledge, are being used in a deliberative dialogue process with Federal government policy partners to prioritize analysis of data collected in a new Canadian population–based survey using the Composite Abuse Scale (Revised)–Short Form. These priorities are intended to inform the staging and framing of forthcoming analyses to best address those narratives (of either or both types) found to be most problematic and in need of immediate attention. We will provide findings to date from the media analysis, and learnings from the formative part of the deliberative dialogue process. These new Canadian data provide a unique opportunity to create compelling evidence-based narratives to dispel existing myths, and, importantly, to push back against malicious and hateful messages designed to sow confusion and division.

## What Are Australian Women’s Experience of Reproductive Coercion and Abuse?

### Molly Wellington^1^, Laura Tarzia^1^, and Kelsey Hegarty^1,2^

^1^The University of Melbourne, Melbourne, Victoria, Australia

^2^The Royal Women’s Hospital, Melbourne, Victoria, Australia

Reproductive coercion (RC) and abuse are terms used to describe a set of controlling behaviors that take away women’s choices over their reproductive health. The behaviors that encompass RC include contraceptive sabotage (where contraceptives can be tampered with, or destroyed against a woman’s wishes), pregnancy pressure or coercion (where a woman is manipulated to become pregnant through coercion or threats), and controlling the outcome of a pregnancy (where a woman is forced to continue a pregnancy or terminate a pregnancy against her wishes). RC is associated with many negative health impacts, including poor mental health and sexually transmitted infections. Little is known about women’s experiences of RC and abuse and the impacts on their lives as well as their interactions with health professionals. To address this gap, this project aimed to answer the following research questions: “What are women’s experiences of RC and abuse?” “What do women experiencing RC and abuse want from their clinicians?” This qualitative research project involved recruiting women who had ever experienced someone trying to make them pregnant when they didn’t want to be, tried to force them to have an abortion or continue a pregnancy when they didn’t want to. Recruitment was done through social media and university student portals. Interviews were conducted either face to face or over the phone and were transcribed verbatim and de-identified. Interview transcripts were analyzed thematically to identify key themes. Transcripts were cross-coded by members of the research team to ensure rigor. The findings of this study highlighted the complexity of the issue of RC. Different to previous literature, this study captured experiences that included both behaviors of direct threats and abuse as well as more subtle coercion. Women interviewed were from diverse cultural backgrounds, with many experiencing RC in a country other than Australia and highlighted the need to understand this form of abuse with a cultural lens. Women believed that clinicians were well placed to have conversations and respond. This research has identified and explored a broader experience of RC than has been investigated before and could inform how we identify and measure it in the future.

## WHO’s Curriculum Improves Timor-Leste Nursing and Midwifery Students’ Knowledge, Attitudes, and Confidence in Responding to Domestic and Sexual Violence

### Kayli Wild^1^, Leesa Hooker^1^, Lidia Gomes^2^, Angelina Fernandes^3^, Luisa Marcal^4^, Guilhermina de Araujo^1^, and Angela Taft^1^

^1^La Trobe University, Melbourne, Victoria, Australia

^2^Universidade Nasional Timor Lorosa’e, Dili, Timor-Leste

^3^Instituto Superior Cristal, Dili, Timor-Leste

^4^Psychosocial Recovery and Development in East Timor (PRADET), Dili, Timor-Leste

All health providers are required to provide first-line support to survivors of violence, and these skills need to be embedded in their pre-service University training. This is of critical importance in countries such as Timor-Leste, where 47% of women have experienced physical or sexual violence from their partner in the past 12 months. The World Health Organization is developing a global curriculum for health providers responding to violence against women, and Timor-Leste is one of the first low-income countries to pilot it as pre-service training. This research examines what content and teaching methods work in low-resource settings, and what creates ownership and widespread uptake of the curriculum by Universities. The curriculum was adapted for the local context by a working group from one Australian University, two Timorese Universities, and a domestic violence advocacy service. Video-based learning materials were developed in the local language, based on formative research with 46 midwives and 28 survivors of violence. The curriculum was piloted three times with a total of 55 students. Pre- and post-training questionnaires assessed changes in students’ knowledge, attitudes, and self-confidence in responding to domestic and sexual violence. The evaluation also included class observation and qualitative interviews with students and lecturers. After each pilot, the proportional changes for each question were analyzed, and the working group improved the content in areas showing limited change. Overall mean score changes were assessed using a Mann–Whitney *U* test, with significance at *p* < .05. The results showed significant improvement in total knowledge scores, which increased progressively with each pilot. The largest change was observed in attitudes which tolerate violence. The qualitative findings highlight the need for videos, guest speakers, and role-plays to enhance student learning. This research demonstrates that significant change in students’ knowledge, attitudes, and confidence in responding to violence is possible, but requires careful attention to refining content based on evaluation results over successive pilots, and ongoing mentoring of lecturers. The widespread uptake of the curriculum by Universities in Timor-Leste illustrates the importance of ownership established through collaborative partnerships and building in time and resources to tailor global training packages to the local context.

## Modeling the Pathways Between Interpersonal Trauma and Opioid Use

### Jessica R. Williams^1^, Veronica Cole^2^, Susan Girdler^1^, and Martha Cromeens^1^

^1^University of North Carolina at Chapel Hill, Chapel Hill, North Carolina, USA

^2^Wake Forest University, Winston-Salem, North Carolina, USA

**Problem Statement:** Individuals who have experienced interpersonal trauma (e.g., intimate partner violence, sexual assault, adverse childhood experiences) are at increased risk of developing chronic pain conditions, being prescribed opioids, and developing an opioid use disorder. Although these relationships are well established, the mechanisms that lead to this increased risk are complex and poorly understood.

**Purpose:** This study aimed to better understand these mechanisms by testing theoretical links between interpersonal trauma, stress, cognitive functioning, depressive symptoms, pain symptoms, and opioid use patterns.

**Study Design:** A cross-sectional, observational design was used to meet the aims of this study.

**Sample:** The sample consisted of 235 individuals recruited from the central North Carolina region using community-based and online recruitment strategies (e.g., study flyers, listservs, message boards). Eligibility criteria were (a) ≥18 years old, (b) able to complete a survey in English, and (c) self-reported history of at least one type of interpersonal trauma (i.e., intimate partner violence, sexual assault, and/or adverse childhood experiences).

**Data Collection Approach:** Participants completed a confidential online survey. Validated measures were used to assess constructs of interest including demographics (sex, age, race/ethnicity, socioeconomic status), interpersonal trauma (intimate partner violence, sexual assault, adverse childhood experiences), perceived stress, cognitive functioning, depressive symptoms, pain symptoms (interference, intensity), and opioid use/misuse.

**Analyses:** A series of regression analyses were conducted to examine relationships between variables and inform variable selection for a structural equation model. Structural equation modeling was used to examine the role of the proposed intervening variables in the association between interpersonal trauma pain symptoms and opioid use/misuse.

**Results:** Preliminary results indicate that perceived stress and depressive symptoms are important mechanisms of the relationship between interpersonal trauma, pain, and opioid use/misuse. These relationships are moderated by sex and age.

**Implications:** This study provides a deeper understanding of the pathways between interpersonal trauma and opioid use, which will help advance prevention and treatment strategies aimed at reducing the deleterious effects of opioid use disorder in at-risk populations.

## Building Sustainable Partnerships Between Community Health Centers and Domestic Violence Programs: Recommendations From a Community Engagement Forum

### Jessica R. Williams^1^, Cassandra Rowe^2^, and Susannah Matthai^1^

^1^University of North Carolina at Chapel Hill, Chapel Hill, North Carolina, USA

^2^North Carolina Coalition Against Domestic Violence, Durham, North Carolina, USA

**Problem Statement:** Individuals who experience domestic violence (DV) are more likely to experience adverse health outcomes, including acute injury and long-term physical and emotional health consequences. Coordinating services between community health centers and DV agencies is a critical strategy for ensuring the health care needs of survivors are met. Barriers often exist, however, in establishing and sustaining these partnerships.

**Purpose:** A community forum was conducted to bring together key stakeholders to discuss priority health needs of survivors and identify ways community health centers and DV agencies can work together to address these needs. The forum results will inform a state-wide initiative to improve and integrate health care responses to DV.

**Study Design:** Participatory action and community engagement approaches were used to meet the aims of this qualitative project.

**Sample:** Twenty-six stakeholders from the North Carolina triangle region participated in the event, including health providers, DV advocates and providers, administrators, care managers, social workers, researchers, students, and community members.

**Data Collection Approach:** Stakeholders participated in a group priority sort activity to determine priority health care needs and populations in need of services. Participants also conducted a SWOT (strengths, weaknesses, opportunities, and threats) analysis, brainstorming strategies to capitalize on new partnerships and opportunities to improve services.

**Results:** Participants identified two priority health need categories: (a) health care service needs, focused on increasing access to certain health services (e.g., trauma-informed health care services, bilingual services) and (b) community needs, focused on improving social determinants of health (e.g., housing, transportation). Vulnerability and resource availability were the most important considerations when determining priority populations. SWOT analysis findings showed consensus regarding the need for improved DV training for health care providers and led to resounding support for state-wide roll out of trauma-informed care training. Participants explained this should also encompass increased awareness of culturally competent care, cross-organizational training, and an established referral and follow-up process.

**Implications:** The forum cultivated coordination and communication between those working to improve the health of DV survivors and by identifying service gaps and developing recommendations for next steps. This methodology used can inform other local efforts aimed at establishing partnerships and priorities between health and DV agencies.

## Promoting Safety and Health for Women and Youth Exposed to Violence: Advancing Health Practice, Education, Policy, and Research

### Lindsay Williams^1^

^1^UCLA School of Nursing, Los Angeles, California, USA

Women Veterans are the largest Veteran population, yet have significant mental health disparities, greater than both civilian women and Veteran men. This is notably related to pervasive trauma before, during, and after military service, prompting the need for consistent mental health care in the outpatient setting. This study used constructivist Grounded Theory methods to explore women Veterans’ decision making to enter mental health outpatient services, and identify aspects of mental health outpatient services that are important to women Veterans. Twelve women Veterans revealed meaningful stories on their experiences of trauma and their use of mental health services. A significant precipitating factor to entry to mental health services is the “tipping point,” after which women Veterans use their peer networks to select a provider. “Trust and Time” can be used to characterize these relationships, because the clinicians’ expertise, consistency, and efforts to establish a partnership will result in positive relationships. Exploration into women Veterans’ perceptions of mental health outpatient care is critical for the creation of gender-specific mental health services. Women’s mental health must be understood within the context of their psychosocial, cultural, and biological circumstances to design interventions that address their unique needs.

## “I Can See the Change in Him . . . ”: A Qualitative Study of Women’s Experience of Alcohol-Related Intimate Partner Violence

### Ingrid M. Wilson^1,2,3^, Kathryn Graham^4,5,6,7,8^, and Angela Taft^1^

^1^La Trobe University, Melbourne, Victoria, Australia

^2^Singapore Institute of Technology, Singapore

^3^University of Liverpool in Singapore, Singapore

^4^Centre for Addiction and Mental Health, Toronto, Ontario, Canada

^5^Dalla Lana School of Public Health, Toronto, Ontario, Canada

^6^Deakin University, Melbourne, Victoria, Australia

^7^Curtin University, Perth, Western Australia, Australia

^8^Western University, London, Ontario, Canada

**Problem:** Alcohol misuse is recognized globally as a risk factor in male perpetration of violence against their intimate partner. Heavy and binge drinking patterns are associated with heightened fear and more severe violence. While these associations have been demonstrated quantitatively, few qualitative studies exist which offer insight into the complexity of the lived experience of survivors of alcohol-related intimate partner violence (IPV) to inform the design and development of appropriate interventions.

**Purpose:** The central question of this study is, How do female survivors of alcohol-related IPV account for the role of alcohol in their experience of abuse and violence?

**Method:** This qualitative study drew on in-depth interviews with 18 women from Victoria, Australia, aged 20 to 50 years. Participants were recruited through the community who reported feeling afraid when their current/former male partner drank alcohol. Interviews explored drinking behaviors and contexts, male partner’s drinking patterns and abusive behaviors, and women’s responses and safety strategies. Interviews were analyzed using a constructivist grounded theory approach.

**Results:** The construct of the “changed man” was central to the women’s understanding of their partner’s alcohol-related aggression. The “changed man” took several different forms, and alcohol was viewed as a culpable factor in their partner’s abuse, contributing to a stark dichotomy where they feared the “changed” (alcohol-affected) man, and loved the “good” (sober) man. However, the women also presented a more complex and nuanced picture of alcohol and violence, disinhibition, and control. Importantly, alcohol was not viewed by participants as an excuse, as women still held their partner accountable for his drinking and violence. The findings showed that partner alcohol use was closely linked with women’s fear and hence became a key focus for how women made sense of their experience of the abusive relationship.

**Implications:** This study invites discussion on what has historically been an uncomfortable topic within family violence research and practice—alcohol’s involvement in IPV perpetration. It highlights the gendered nature of both alcohol use and violence and raises questions for how services can engage with and support women for whom partner alcohol use is intertwined with their experience of abuse, without disempowering women further by silencing their experience.

## Indigenous Māori Women Keeping Safe in Unsafe Relationships Amid Structural Entrapment

### Denise Wilson^1^, Alayne Hall^1^, Karina Cootes^1^, Juanita Sherwood^2^, and Debra Jackson^3^

^1^Auckland University of Technology, Auckland, New Zealand

^2^University of Sydney, Sydney, New South Wales, Australia

^3^University of Technology Sydney, Sydney, New South Wales, Australia

**Problem:** Research with Indigenous peoples is often framed within dominant worldviews. Such approaches generally produce deficit-focused findings that do little to respond in relevant and meaningful ways.

**Significance:** Māori (Indigenous peoples of Aotearoa New Zealand) disproportionately bear the burden of partner violence and the associated prevalence, serious harm, and mortality. This persistent problem is resistant to amelioration, affecting the well-being of those whānau (extended family networks) affected. The complexities affecting whānau is the added layers of the ongoing effects of colonialism; dispossessions of land, language, and cultural practices; intergenerational effects of historical trauma; systemic racism; and socioeconomic and political disenfranchisement.

**Purpose:** E Tū Wāhine, E Tū Whānau aimed to understand how Māori women keep safe in unsafe relationships, focusing on their strengths and strategies to keep themselves, their children, and others safe.

**Study Design:** E Tū Wāhine, E Tū Whānau is research funded by the New Zealand Royal Society Marsden Fund to produce “new” knowledge about how Māori (Indigenous people of Aotearoa New Zealand) women keep safe in unsafe relationships. Using a qualitative research design informed by an Indigenous (Kaupapa Māori and Mana Wāhine) methodology that has a decolonizing component.

**Sample:** *E Tū Wāhine, E Tū Whānau* included a total of 69 Māori participants: 30 women, eight men, 16 elders and stakeholders, 16 young Māori women.

**Data Collection and Analysis:** The researchers undertook semi-structured audio-recorded interviews. Transcripts were analyzed with Charmaz’s grounded theory to recover culturally informed contemporary knowledge about how Māori women keep safe in unsafe partner relationships.

**Results:** Māori women utilize a range of strategies to keep themselves and their children safe. Māori women viewed their partners as having potential, and their children provided a lifelong connection. When they exhaust strategies for keeping safe and needed to leave their partner, they encounter systemic entrapment—people who are judgmental and racist, having their children taken, and often denied the support and resources they are entitled to receive.

**Implications:** This study highlights Māori women’s extensive strategies for keeping them and their children safe amid significant partner and systemic entrapment.

