# Corrigendum to “NNVAWI Abstracts”

**DOI:** 10.1177/2333393620967636

**Published:** 2020-10-08

NNVAWI Abstracts. (2020). *Global Qualitative Nursing Research*. https://doi.org/10.1177/2333393620932494

In the above-referenced article, for the abstract titled “*An Interpretive Descriptive Study About the Mental Health Impact of Cumulative Lifetime Violence in Men: Interviews From a Sample in the Men’s Violence, Gender, and Health Study, New Brunswick, Canada*”, one of the author was missed.

The correct authorship is as given below:

Petrea Taylor^1^, Kelly Scott-Storey^1^, Sue O’Donnell^1^, Judith Wuest^1^, Jeannie Malcolm^1^, and Charlene Vincent^1^

^1^University of New Brunswick, Fredericton, New Brunswick, Canada

